# Gas‐Phase Electronic Structure of Phthalocyanine Ions: A Study of Symmetry and Solvation Effects

**DOI:** 10.1002/advs.202307816

**Published:** 2024-01-15

**Authors:** Stefan Bergmeister, Lisa Ganner, Milan Ončák, Elisabeth Gruber

**Affiliations:** ^1^ Institute for Ion and Applied Physics University of Innsbruck Technikerstraße 25 Innsbruck 6020 Austria

**Keywords:** electronic transitions, helium nanodroplets, messenger spectroscopy, phthalocyanine, UV–vis gas‐phase spectroscopy

## Abstract

Research into and applications of phthalocyanines (Pc) are mostly connected to their intriguing electronic properties. Here, messenger‐type UV–vis spectroscopy of two metal‐free ions from the phthalocyanine family, cationic H_2_Pc^+^ and H_2_PcD^+^, along with their hydrates is performed. They show that the electronic properties of both ions can be traced to those in the conjugate base, Pc^2–^, however, they are affected by state splitting due to the reduced symmetry; in the H_2_Pc^+^ radical cation, a new band appears due to excitations into the singly‐occupied molecular orbital. Quantum chemical spectra modeling reproduces all important features of the measured spectra and provides insight into the nature of electronic transitions. Hydration of the ions has only a mild effect on the electronic spectra, showing the stability of the electronic structure with respect to solvation effects.

## Graphical abstract

Through systematic spectroscopic studies and quantum chemical spectra modeling, the electronic structure of phthalocyanine cations, including bare and deuterated phthalocyanine, along with their hydrated forms is elucidated. They observe and rationalize the splitting of electronic states due to reduced symmetry, as well as the emergence of a new spectral band by moving from a closed‐shell configuration to radicals.

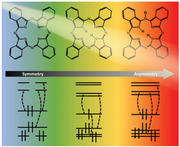

## 1 Introduction

Phthalocyanine (H_2_Pc) and its metallic derivatives (MPc) belong to the large family of aromatic organic compounds and received considerable attention within the last years due to their exceptional electronic, optical, and magnetic properties.^[^ ^1^ ^]^ These molecules find widespread use in various technical applications, including optical data storage, gas sensors, and dyes in infrared laser systems. Of particular interest is their potential application as nonlinear optical materials,^[^ ^2^ ^]^ sensitizers in photochemical reactions or photovoltaic cells,^[^ ^3^ ^]^ photodynamic reagents in cancer therapy,^[^ ^4^ ^]^ liquid crystals,^[^ ^5^ ^]^ enzyme‐like catalysts,^[^ ^6^ ^]^ pigments and dyes.^[^ ^7^ ^]^ Additionally, MPc can serve as model systems for studying biologically relevant, albeit more delicate, molecules such as heme and chlorophyll, owing to their structural similarities.

Spectroscopic investigations of H_2_Pc and its derivatives have been carried out in various environments, including surfaces,^[^ ^8^ ^]^ solutions^[^ ^9^ ^]^ or molecular matrices.^[^ ^10^ ^]^ A range of techniques has been employed, including Raman spectroscopy,^[^ ^9a^ ^]^ UV–vis absorption spectroscopy,^[^ ^10^, ^11^ ^]^ (FT)IR spectroscopy,^[^ ^11^, ^12^ ^]^ and fluorescence spectroscopy.^[^ ^10^, ^13^ ^]^ Gas‐phase measurements, in comparison to studies conducted in solution or on surfaces, offer the advantage of revealing the intrinsic photophysical properties of the molecular systems without interference from the surrounding environment and allow for the controlled study of such interactions.^[^ ^14^ ^]^

Another crucial step in improving the quality and reliability of spectroscopic data is cooling the molecules to reduce the distribution of populated quantum states. In the past, various rare‐gas matrices were utilized to create a cold, minimally interacting environment for investigating pure H_2_Pc^10^, ^15^ ^]^ with helium being identified as the most suitable rare gas due to its weak interaction with other atoms or molecules.^[^ ^15d^ ^]^

Another well‐established method to study molecules in the gas phase at extremely low temperatures involves embedding them in helium nanodroplets (HNDs).^[^ ^16^ ^]^ These droplets, composed of weakly bound helium atoms, serve as a cold matrix that captures any molecule it collides with.^[^ ^17^ ^]^ The fundamental issue of micro‐solvation, which treats how the helium matrix affects spectroscopic features, has been extensively examined for H_2_Pc, primarily using fluorescence excitation and emission spectroscopy.^[^ ^2^, ^10^, ^18^ ^]^ The doubling of emission lines, absent in the gas phase, has been attributed to interactions with the surrounding helium layer.^[^ ^15b^ ^]^ Additionally, van der Waals clusters of H_2_Pc with Ar*_n_* (*n* = 1–4)^[^ ^13^, ^19^ ^]^ and with H_2_O^[^ ^10c,d^ ^]^ have been formed through sequential pickup within the helium nanodroplets and subjected to spectroscopic analysis. In the case of the H_2_Pc‐H_2_O clusters, three isomeric complexes displaying different electronic excitation energies have been discussed.^[^ ^10c^ ^]^ Recently, high‐resolution 2D electronic spectroscopy in the gas phase has been applied to H_2_Pc to investigate the nonlinear response of isolated chromophores in various nanosystems with femtosecond time resolution.^[^ ^15d^ ^]^ The spectroscopic width of H_2_Pc has been observed to be temperature and energy‐dependent when performing 2D spectroscopy on H_2_Pc in helium clusters (≈0.37 K) and in neon clusters (≈10 K).

In this contribution, our focus lies on the electronic spectroscopy of H_2_Pc cations, which are formed in multiply‐charged HNDs.^[^ ^20^ ^]^ We examine how the spectrum changes as we alter the environment, such as by forming deuterated H_2_Pc species or by attaching single (1 up to 15) water molecules. To eliminate an influence arising from solvation within HNDs, we employ a newly developed method.^[^ ^21^ ^]^ This method utilizes HNDs to create and cool molecular ions to sub‐Kelvin temperatures before gently extracting them from the helium matrix through collisions with additional helium gas at room temperature. This approach enables the efficient formation of weakly bound helium‐tagged molecular ions, which are suitable for gas‐phase action spectroscopy by detecting the loss of the messenger helium following photoabsorption.^[^ ^22^ ^]^ While the UV–vis spectrum of H_2_Pc^+^ significantly differs from that of H_2_PcD^+^, the formation of hydrogen bonds with water molecules results in a slight blueshift of the broad absorption bands observed in both the visible and ultraviolet regions. Through quantum chemical analysis, we rationalize the dissociation patterns and track the character of spectral features to symmetry breaking, starting from the Pc^2–^ conjugate base of very high symmetry (D_4h_) through H_2_Pc and open‐shell H_2_Pc^+^ (D_2h_) to the deuterated H_2_PcD^+^ ion (C_s_).

## 2 Results and Discussion

In this section, we delve into the spectroscopic properties of H_2_Pc^+^(H_2_O)*_n_* and H_2_PcD^+^(H_2_O)*_n_*, examining their absorption spectra and structural characteristics.

### 2.1 H_2_Pc^+^(H_2_O)*_n_*

**Figure 1:**
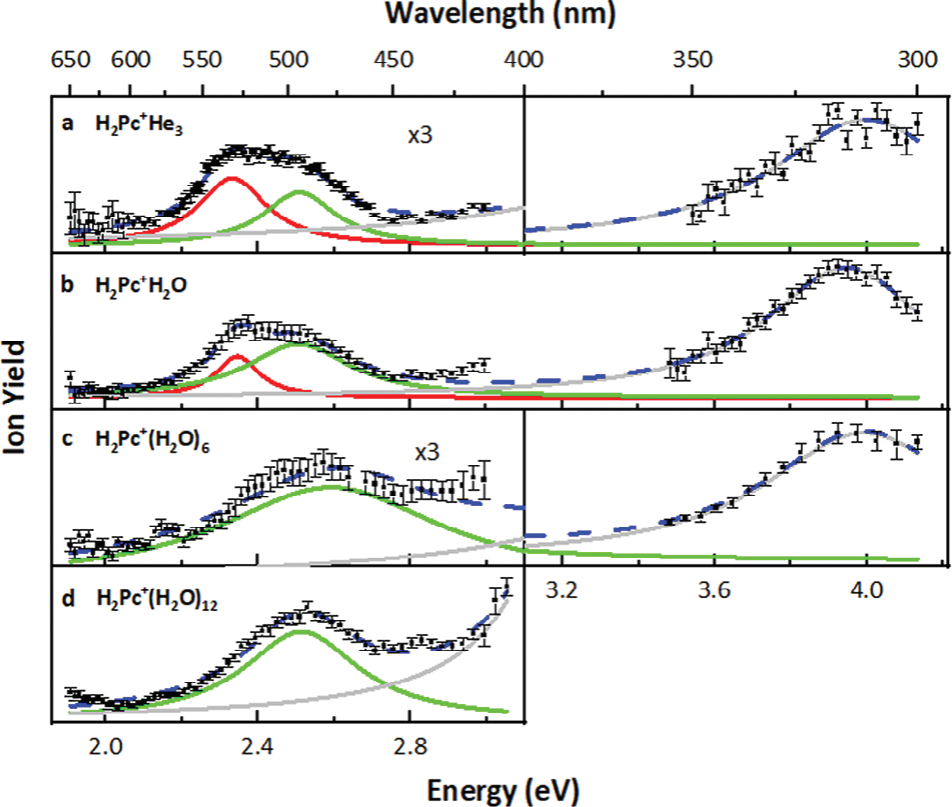
Wavelength scan of different precursor ions. Subfigure a) shows a wavelength scan for H_2_Pc^+^He_3_, subfigure b–d) for the precursor H_2_Pc^+^(H_2_O)*_n_* with *n* = 1, 6, 12. In all subfigures, multi‐Lorentzian fits were performed, in subfigures a) and b), three peaks were fitted (red, green, and grey), together with a cumulative fit (blue). In subfigures c) and d), data were each fitted with a multi‐Lorentzian fit (blue) and with only two Lorentzian (grey and green). Due to visibility reasons, the region between 1.86 and 3.1 eV in subfigure a) and c) was multiplied by a factor of three. In the case of helium‐tagged H_2_Pc^+^, we obtained a count rate of ≈250 cps for the parent peak H_2_Pc^+^He_3_ and a count rate of ≈40 cps for the photofragment H_2_Pc^+^ (at 316 nm). In the case of the hydrated H_2_Pc^+^, we obtained ≈1760 cps for the selected parent peak H_2_Pc^+^(H_2_O)_6_, and ≈160 cps for the photofragment H_2_Pc^+^ (at 413 nm).

We start our discussion with the spectroscopy of pure H_2_Pc^+^ tagged with three He atoms. In **Figure** **1**a, the Y_Norm_ yield (normalized photofragment yield) of H_2_Pc^+^ is plotted as a function of the laser photon energy. Two prominent and broad absorption bands, one in the visible wavelength range and one in the UV, are evident. A multi‐Lorentzian fit reveals three maxima at ≈2.3, 2.5, and 4.0 eV, respectively. Figure 1b–d presents wavelength scans of mass‐selected H_2_Pc^+^(H_2_O)*_n_* cluster ions, where *n* equals 1, 6 and 12. Additional absorption spectra of H_2_Pc^+^‐water clusters can be found in the SI, Figure S2 (Supporting Information). While these spectra share similarities, there is a noticeable trend: a slight narrowing and a blueshift of the absorption band in the visible range become apparent as more water molecules are attached to the clusters. Such effects have been reported for other gas‐phase water‐biomolecule clusters, for example for uracil‐water clusters.^[^ ^23^ ^]^

**Figure 2:**
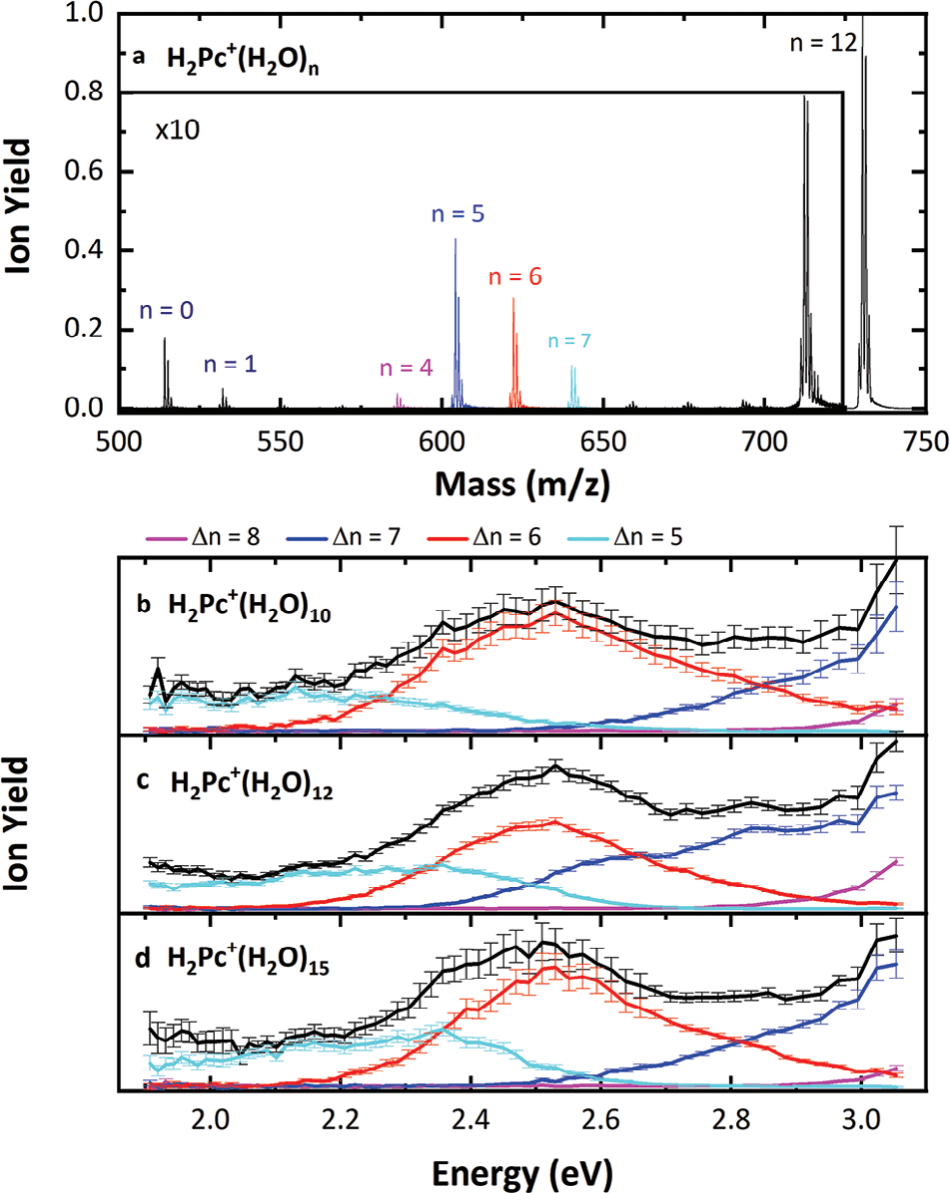
a) Mass spectrum of the precursor H_2_Pc^+^(H_2_O)_12_ when photofragmentation occurs. The fragments are plotted in different colors and are multiplied by a factor of ten due to visibility reasons. Section b–d) shows the wavelength dependence of each photofragment for the precursor H_2_Pc^+^(H_2_O)*_n_*, *n* = 10,12,15. The black curve is the sum of all photofragments after 1‐photon absorption.

In Figure 1a, helium atoms serve as messengers for photoabsorption, whereas in Figure 1b–d, the loss of water molecules is used to extract the absorption spectra. For example, the mass spectrum associated with the wavelength scan in Figure 1d is shown in **Figure** **2**a. Here, the quadrupole mass filter only allows ions with a mass‐to‐charge ratio of 730 (corresponding to H_2_Pc^+^(H_2_O)_12_) to pass. The detected peak at a mass‐per‐charge ratio of 18 below the parent peak represents the loss of one water molecule due to collisions with residual gas after passing through the mass filter. Upon photoabsorption, two photofragment distributions emerge: one corresponding to the loss of 11 or 12 water molecules and the other to the loss of five to eight water molecules. The high abundance of H_2_Pc^+^(H_2_O)_5_ indicates a particularly stable configuration. We disregard photofragments that lose more than eight water molecules, as they stem from multiphoton absorption processes, as confirmed by laser power dependence measurements (see Figure S1, Supporting Information).

Photofragments that lose less than eight water molecules show unique wavelength‐dependent absorption features, independent of the selected parent ion. The corresponding partial ion yields for different precursor ions, H_2_Pc^+^(H_2_O)*_n_* with *n* = 10, 12, 15 are displayed in Figure 2b–d. A clear trend of sequential water molecule loss with increasing absorbed energy is evident. Assuming the entire photon energy is converted into thermal energy upon excitation and equally distributed to the attached water molecules, we deduce a binding energy of 0.42(4) eV per H_2_O molecule from the experimental spectra (for more details see Figure S4, Supporting Information).

**Figure 3:**
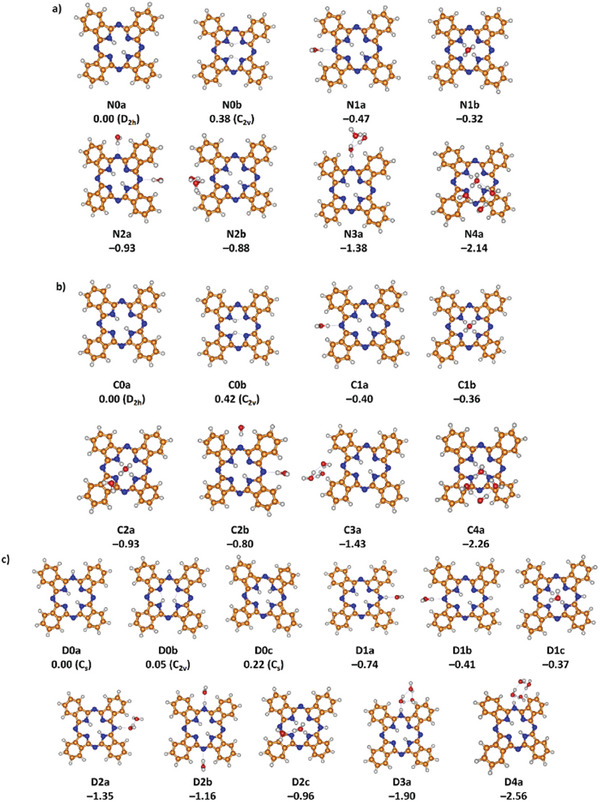
Selected isomers of a) H_2_Pc(H_2_O)*_n_*, b) H_2_Pc^+^(H_2_O)*_n_*, c) H_2_PcD^+^(H_2_O)*_n_*, *n* = 0–4, along with the respective symmetry group and the relative stability for *n* = 0 and hydration energy for *n* = 1–4 corresponding to the M + *n* H_2_O → M(H_2_O)*_n_* reaction, M = H_2_Pc, H_2_Pc^+^, H_2_PcD^+^ (in eV). Calculated at the ωB97XD/cc‐pVDZ level of theory. See Figures S6–S9 (Supporting Information) for further structures.

In **Figure** **3**a,b, we show the calculated isomers of H_2_Pc and H_2_Pc^+^. In the most stable structure of both molecules, N0a/C0a, opposite inner nitrogen atoms are protonated, resulting in high D_2h_ symmetry. In the second most stable isomer of C_2v_ symmetry, N0b/C0b, the neighboring inner nitrogen atoms are protonated. Charging H_2_Pc almost does not influence the relative stability as the least bound electron occupies a delocalized π orbital (see below). As expected, structures with protonated outside nitrogen atoms are considerably less stable, see Figures S6 and S7 (Supporting Information).

As the positive charge is markedly delocalized in H_2_Pc^+^, hydration patterns in H_2_Pc and H_2_Pc^+^ are very similar. When a first water molecule is attached, two possible hydration sites can be distinguished, namely the outer nitrogen atoms and the central N/NH groups. Starting with a second water molecule, water clusters might be formed as well. Our calculations show that for both H_2_Pc and H_2_Pc^+^, the first water molecule is preferentially attached to an outer nitrogen atom, isomer N1a/C1a, in agreement with a recent study on neutral H_2_Pc‐H_2_O clusters.^[^ ^10c^ ^]^ However, the alternative structure with a water molecule attached to the central protons, N1b/C1b, breaking the planarity of the phthalocyanine ring, lies close in energy. For two water molecules, two hydrogen bonds with outer nitrogen atoms are preferentially formed in H_2_Pc(H_2_O)_2_ while a structure with a water dimer is more stable for H_2_Pc^+^(H_2_O)_2_. For larger clusters, water clustering seems to be preferred compared to attaching water molecules to several nitrogen sites (see Figures S8 and S9, Supporting Information for further structures). The calculated hydration energies are ≈0.5 eV per H_2_O, slightly higher than the hydration energy deduced from the experiment in case the whole excitation energy is used for water evaporation. Note that the experimental value is derived for larger clusters for which a decrease in average hydration energy is expected. Also, one might assume that the particular stability of H_2_Pc^+^(H_2_O)_5_ could be connected to the formation of a hydrogen bonding bridge between the central nitrogen atoms and the outer ones that are evident already in N4a and C4a; however, more extensive exploration of possible structures would be needed to confirm this.

**Scheme 1:**
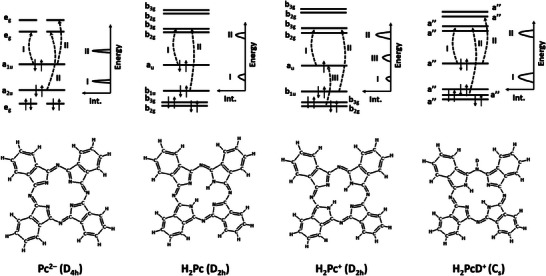
Simplified comparison of the electronic structure and the main spectral features in Pc^2–^, H_2_Pc, H_2_Pc^+^, and H_2_PcD^+^. See Figure 4 and Figures S12–S15 (Supporting Information) for spectra, transition energies, oscillator strengths, and transition character.

To understand the features observed in the electronic spectra of H_2_Pc^+^(H_2_O)*_n_*, we analyze the electronic structure in molecules with the phthalocyanine ring, starting with Pc^2–^, the conjugate base of H_2_Pc, see **Scheme** **1**. The Pc^2–^ anion has a high symmetry, D_4h_, and degeneracies are possible due to the presence of the C_4_ axis. When H_2_Pc or H_2_Pc^+^ is formed, symmetry is reduced to D_2h,_ and degeneracies are split. The addition of a deuterium atom lowers the symmetry even further to C_s_. These effects are directly reflected in the electronic spectra. Moreover, the open‐shell character of H_2_Pc^+^ allows a new class of electronic transitions into the singly‐occupied molecular orbital (SOMO).

**Figure 4:**
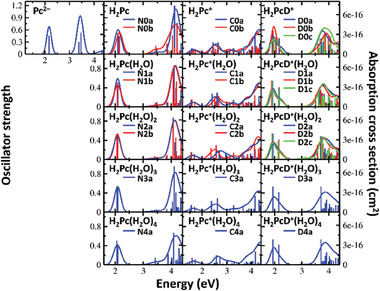
Calculated electronic transitions (bars) and spectra modeled using the linearized reflection principle (curves) of Pc^2–^, H_2_Pc(H_2_O)*_n_*, H_2_Pc^+^(H_2_O)*_n_*, H_2_PcD^+^(H_2_O)*_n_*, *n* = 0–4. Calculated at the TD‐BMK/aug‐cc‐pVDZ//ωB97XD/cc‐pVDZ level. See Figure 3 for isomer structures.

**Figure** **4** shows the electronic transitions in the calculated structures along with spectra modeled within the linearized reflection principle. We start our discussion with electronic spectra of Pc^2–^ and H_2_Pc. Although they were not probed experimentally here, they provide a basis for understanding the transitions in H_2_Pc^+^ and H_2_PcD^+^. In Pc^2–^, the three lowest bright transitions correspond to e_g_ ← a_1u_, e_g_ ← a_2u,_ and e_g_ ← a_1u_, with two higher‐lying transitions lying very close to each other, forming one band (see Figure 4 and Scheme 1). As expected, valence orbitals are delocalized over the π system (Figure S12, Supporting Information). Note that all transitions are doubly degenerate and thus split when the symmetry is lowered. This is indeed observed in H_2_Pc where the e_g_ orbitals split into b_2g_ and b_3g_ orbitals, forming broader bands in the calculated spectrum. Within 4.3 eV, there are six bright electronic transitions present in H_2_Pc (see Figure 4; Figure S13, Supporting Information). Two transitions of the b_2g_/b_3g_ ← a_u_ character form a band at ≈2.1 eV, the second band at ≈4.2 eV corresponds mainly to b_2g_/b_3g_ ← b_1u_ transitions. The spectra are only mildly sensitive to the position of the hydrogen atoms in isomers N0a/N0b. We note that the broad, structureless peaks are predicted due to the usage of the linearized reflection principle.

Upon ionization of H_2_Pc, an electron is removed from the HOMO orbital of a_u_ symmetry. The open‐shell character of the H_2_Pc^+^ ion significantly alters the spectrum, introducing several new electronic transitions within the energy range covered by the experiment (Figure 4). Our spectra modeling predicts three bands centered at ≈1.7, 2.6, and 4.3 eV. The first and third bands roughly correspond to the first and second bands found in Pc^2–^/H_2_Pc (see Figure S14, Supporting Information). The second band newly appears as it corresponds to an excitation from a doubly occupied b_2g_/b_3g_ orbital into the a_u_ SOMO orbital as shown in Scheme 1. Again, the position of the hydrogen atoms has a rather mild effect on the spectral shape.

Comparing the modeled spectrum of H_2_Pc^+^ to the experimental data, the lowest‐lying band falls outside the laser energy range and is not observed. However, the following two bands are well reproduced by our modeling with respect to their position, width, and relative intensity of ≈1:3. The structure of the lower‐lying band with two Lorentzian functions used for fitting in Figure 1 can be explained by the e_g_ orbital in Pc^2–^ splitting to b_2g_/b_3g_ in H_2_Pc^+^. The calculated splitting of 0.21 eV for the a_u_ ← b_2g_/b_3g_ transitions is in good agreement with the 0.18 eV splitting obtained through the experimental fit. The higher‐lying band is calculated to lie by ≈0.3 eV higher than the experimental data, most probably due to the limitations of the employed quantum chemical method.

The spectra of the hydrated species (see Figure 4) give us an insight into the possible origin of the broadening and shifting of the spectra upon hydration (see Figure 1b–d). Electronic transitions in both H_2_Pc(H_2_O)*_n_* and H_2_Pc^+^(H_2_O)*_n_* are somewhat sensitive to the position of the water molecules, with a difference of the calculated maximum of the second band in C_1a_ and C_1b_ of 0.09 eV. With the increasing number of water molecules, the low‐lying band undergoes minimal changes, while the higher‐lying bands become broader and shift slightly to higher energies. As the transitions occur between highly delocalized orbitals, adsorbed water molecules have only a limited effect on the excitation energy. However, their presence still induces a subtle blue shift. This can be explained by the selective stabilization of the ground electronic state by the adsorbed water molecules while the electronically excited is either almost unaffected or even slightly destabilized by the presence of water.

### 2.2 H_2_PcD^+^(H_2_O)*_n_*

In the Next Step, we Investigate the spectroscopy of deuterated H_2_Pc^+^. We decided to deuterate instead of protonate our sample, due to the lower contribution of the H_2_Pc^+^ isotopes to the H_2_PcD^+^ rather than to the H_2_PcH^+^ peak, which simplifies the evaluation. Deuterated H_2_Pc^+^ ions are generated by predoping multiply charged HNDs with deuterium, leading to the formation of D_3_^+^ ions distributed on the HND surface.^[^ ^24^ ^]^ Further pick‐up of D_2_ molecules results in the formation of (D_2_)*_n_* D^+^ ions. Doping these predoped HNDs with H_2_Pc leads to the formation of H_2_PcD^+^(D_2_)*_n_* via proton transfer processes. Once again, these ions are gently released from the HND through collisions with He gas in the evaporation chamber. For spectroscopic investigations, the loss of weakly attached (D_2_)*_n_* molecules is used as the messenger for photoabsorption.

**Figure 5:**
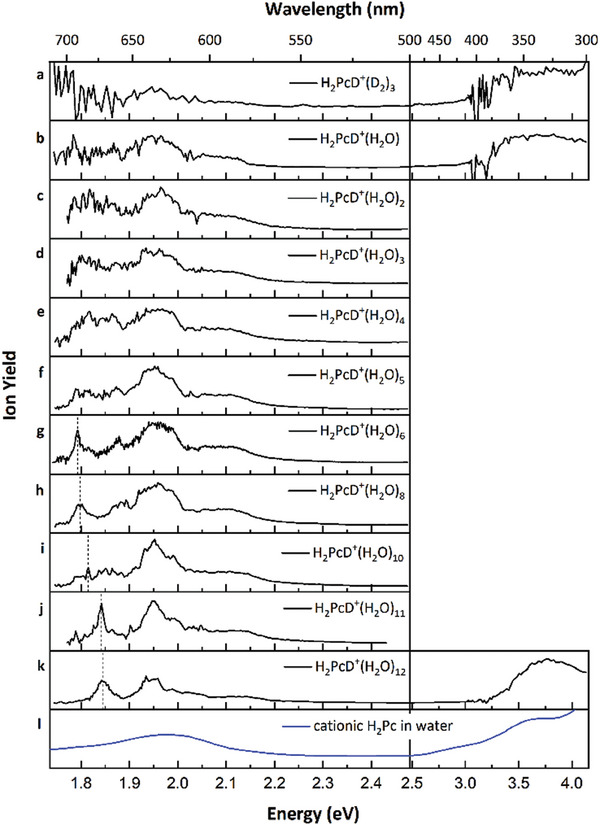
a) Wavelength scan for H_2_PcD^+^(D_2_)_3_; b–k) Wavelength scan for the precursor ions H_2_PcD^+^(H_2_O)*_n_* for *n* = 1–6, 8, 10–12. In the case of deuterium tagged H_2_PcD^+^, we obtained a count rate of ≈1360 cps for the parent peak H_2_PcD^+^(D_2_)_3_ and a count rate of ≈50 cps for the photofragment H_2_PcD^+^ (at 318 nm). In the case of the hydrated H_2_Pc^+^, we obtained ≈2000 cps for the selected parent peak H_2_PcD^+^(H_2_O)_6_, and ≈35 cps for the photofragment H_2_PcD^+^ (at 582 nm). l) UV–vis spectrum of cationic H_2_Pc solvated in water performed by Bayrak.^[^ ^25^ ^]^

**Figure** **5**a displays the UV–vis wavelength scan of H_2_PcD^+^ with three D_2_ attached. The absorption spectrum exhibits two broad absorption bands, one spanning from ≈1.77 to 2.26 eV and anotherat ≈3.8 eV. A poor signal‐to‐noise ratio due to low laser power hinders the observation of distinct features in the spectrum, especially at laser photon energies lower than 1.9 eV.

Absorption spectra of H_2_PcD^+^(H_2_O)*_n_*, where *n* ranges from 1 to 12, are depicted in Figure 5b–k. These spectra are evaluated similarly to those in Figure 1b–d, with a notable difference in dissociation channels. While the corresponding spectra in Figure 1b–d mainly show the loss of H_2_O molecules, the mass spectra in Figure 5b–k reveal additional fragmentation channels due to the loss of DHO(H_2_O)*_n_* molecules (see Figure S3, Supporting Information). The relative loss of DHO itself increases exponentially with the number of detached water molecules (see Figure S3, Supporting Information, inset). Our analysis considers various fragmentation channels, protonated or deuterated, leading to no discernible spectroscopic differences between these species. As more water molecules are attached, the signal‐to‐noise ratio improves due to a higher ion yield, making the features more distinct. In addition to the reduced noise, we observe changes in the absorption properties. The peak at 1.96 eV narrows as more water molecules solvate the cluster. Moreover, an absorption feature appears at ≈1.79 eV in Figure 5g (indicated with a dashed line), which shifts to higher energies as additional water molecules are added (dashed line in Figure 5h–k). The experimentally derived hydration energy of 0.43(5) eV per H_2_O molecule for H_2_PcD^+^(H_2_O)_12_ clusters (see Figure S5, Supporting Information) is similar to the binding energy of water to the unprotonated H_2_Pc^+^. Earlier UV–vis spectra of cationic H_2_Pc solvated in water^[^ ^25^ ^]^ (data is reproduced in Figure 5l) have shown unstructured broad absorption bands in the visible (max ≈625 nm) and UV (max ≈340 nm) wavelength range. It appears that solvation smoothens out the individual characteristics.

In comparison to fluorescence excitation, spectroscopic investigations utilizing HNDs as an ultracold matrix, which show narrow absorption bands (<0.1 cm^–1^)^[13a^^]^ including the electronic band origin S_1_ ← S_0_ of neat H_2_Pc at 15 089 cm^−1^ (1.87 eV),^[^ ^15^, ^26^ ^]^ no distinct peaks were observed in our measurements. Several factors may contribute here. The main difference between these former fluorescence excitation spectroscopic measurements and our results, besides the fact that we are studying H_2_Pc analogs in their cationic and not neutral form, is, that our measurements include excitation into higher vibrational states, which cannot be resolved well, likely due to a high density of vibrational states with similar Franck–Condon overlap. Additionally, lifetime broadening may be a contributing factor.^[^ ^27^ ^]^ Furthermore, our measurements were conducted at higher temperatures, with dopant ions fully or nearly fully liberated from the He matrix, and we employed an OPO laser system with linewidths on the order of 4–5 cm^–1^. The presence of multiple isomers may also broaden the absorption bands.

Calculated structures of H_2_PcD^+^(H_2_O)*_n_*, with *n* ranging from 0 to 4, are presented in Figure 3c. Among the various protonation possibilities, the most favorable involves adding a proton to an outer nitrogen atom of the H_2_Pc molecule, yielding the isomers D0a and D0b. Protonating an inner nitrogen atom is less favored, as seen in D0c (see Figure S10, Supporting Information for further isomers). The most favorable position for hydration is then the protonated side, represented by isomer D1a. As further water molecules attach, water clusters are predicted to be preferred, with a hydration energy close to 0.6 eV per H_2_O molecule, slightly higher than the experimental value due to the analysis of the cluster with *n* = 12 in the experiment.

Modeled electronic spectra of H_2_PcD^+^(H_2_O)*_n_* ions are shown in Figure 4. As these ions have closed‐shell character and the additional proton does not influence the π system considerably, their electronic structure closely resembles that of the H_2_Pc(H_2_O)*_n_* clusters previously discussed. As a result, the spectrum of H_2_PcD^+^ predicts two broad bands: one centered at ≈1.9 eV, the other at ≈3.8 eV.

The lower‐energy band consists of two transitions that correspond to the e_g_ ← a_1u_ transition in Pc^2–^, with a separation of 0.25 eV between them. The transitions in the higher‐energy peak primarily correlate with e_g_ ← a_2u_ in Pc^2–^, as illustrated in Scheme 1. The calculated bands closely match the experimental results in terms of their position, width, and intensity with a ratio of ≈1:1. As these transitions occur between highly delocalized orbitals (see Figure S15, Supporting Information), the spectra exhibit remarkable similarity among different isomers, regardless of the deuteration site.

When H_2_PcD^+^ forms complexes with water, minimal shifts in the spectral position and intensity are observed, as exemplified for D1a–c isomers in Figure 4. This observation is consistent with the experimental spectra, where any significant changes in the absorption spectra would imply a shift in the deuteration site. With increasing hydration, a slight blueshift is noted in the calculated spectra, up to 0.05 and 0.1 eV for the first and second bands, respectively, in agreement with the experimental findings.

Two spectral features of H_2_PcD^+^(H_2_O)*_n_* observed experimentally in the 1.8–2.4 eV region can be attributed to two distinct transitions corresponding to the e_g_ ← a_1u_ transition in Pc^2–^. The calculated splitting of 0.21–0.25 eV for H_2_PcD^+^(H_2_O)*_n_*, where *n* = 0–4, agrees with the separation of ≈0.1–0.2 eV observed experimentally for various degrees of hydration. The experimental spectrum is more structured as the vibrational progression cannot be reproduced within linearized reflection principle approximation. However, our analysis does not suggest the presence of several isomers with different protonation sites.

## 3 Conclusion

We employed an innovative experimental setup to investigate the spectra of mass‐selected phthalocyanine cations, which were either pure, deuterated, or complexed with water molecules. Our observations reveal broad absorption bands in the ultraviolet and visible regions. To comprehend these spectral characteristics, we established connections between the electronic structures of H_2_Pc^+^ and H_2_PcD^+^ and the high‐symmetry Pc^2–^ anion. While two absorption features in the closed‐shell H_2_PcD^+^ cation correlate directly with e_g_ ← a_1u_ and e_g_ ← a_2u_ transitions in Pc^2–^, an additional band corresponding to the excitation into a SOMO is observed in the spectrum of the H_2_Pc^+^ radical cation. Upon attaching single water molecules (ranging from 1 to 15), we note a slight blueshift and a narrowing of the absorption bands in our experimental data. Our calculations indicate that the initial water molecule preferentially adsorbs to the outer nitrogen atoms in both ions and as more water molecules are introduced, water clusters form. Theoretical analysis further confirms that the electronic transitions between highly delocalized orbitals are only minimally affected by the presence of the adsorbed water molecules.

## 4 Experimental Section

In the experiment, helium nanodroplets with a size of several million helium atoms were produced by supersonic expansion of helium at a pressure of 22 bar and a temperature of 8.5 Kelvin through a 5 µm nozzle. The HNDs were ionized by electron impact, mass‐per‐charge selected by an electrostatic sector, and doped in a differentially pumped chamber which was equipped with a resistively heated oven and a gas inlet system. In this study, the oven was utilized to vaporize H_2_Pc, and the gas inlet system to introduce water vapor. The picked‐up H_2_Pc molecules were attracted to the charged regions on the HND surface and were ionized through charge transfer processes. Further pick‐up of H_2_Pc and/or water molecules results in the formation of cluster ions, denoted as (H_2_Pc)*_n_* ^+^(H_2_O)*_m_*, where *n* and *m* were tuned by varying the oven temperature as well as by the valve‐controlled gas inlet system, respectively. Subsequently, the doped HNDs collided with additional helium gas at room temperature in a separate, differentially pumped evaporation chamber. These collisions serve to reduce the size of the droplets and gently release the helium‐tagged dopant ions from the helium nanodroplet. Depending on the helium pressure in the evaporation chamber, additional collisions of these ejected cluster ions with He atoms reduce the number of He atoms attached to a desired value.

After mass‐to‐charge selection using a quadrupole mass filter, the ions were merged with a laser beam generated by a pulsed OPO laser system (EKSPLA NT242). The resulting products were then directed into a time‐of‐flight mass spectrometer. The mass spectrometer was operated at a frequency of 10 kHz, while the laser operated at 1 kHz, allowing for the recording of mass spectra with and without laser irradiation on an alternating basis. For a more detailed description of the experimental setup, additional information can be found elsewhere.^[^ ^22^ ^]^

From the measured mass spectra, the normalized photofragment yield Y_Norm_ related to absorption using the following formula was calculated:

(1) $$Y_{\mathrm{Norm}}=\left(N_{\mathrm{Frag}}-N_{\mathrm{BG}}\right)/\left(N_{\mathrm{Parent}}\times N_{\mathrm{Photon}}\right)$$

where N_Frag_, N_BG_, N_Parent,_ and N_Photon_ were the integrated counts of the fragments resulting from photoabsorption, the background counts at this mass per charge value without photoabsorption, the parent ion peak without photoabsorption, and the number of photons, respectively. Caution was exercised to select only the desired photofragments and to avoid photoproducts resulting from multiphoton processes. Further details can be found in Figure S1 (Supporting Information).

To analyze the structure, hydration energy, and electronic spectra of specific ions, quantum chemical calculations, optimizing Pc^2–^, H_2_Pc, H_2_Pc^+^, and H_2_PcD^+^ along with their hydrated forms employing density functional theory (DFT) at the ωB97XD/cc‐pVDZ level of theory were performed.^[^ ^28^ ^]^ For hydrated clusters, only representative structures with respect to the bonding patterns were picked to study the influence of the hydration on the electronic spectra (see the Supporting Information for details). The nomenclature of N, C, and D for neutral (H_2_Pc), cationic nondeuterated (H_2_Pc^+^), and cationic deuterated clusters (H_2_PcD^+^) was used, respectively. Additionally, we indicate the number of water molecules and use the letters a–i to represent the relative stability, e.g., D2b refers to the second most stable H_2_PcD^+^(H_2_O)_2_ structure identified. The electronic transitions were calculated Within time‐dependent DFT (TDDFT) theory at the TD‐BMK/aug‐cc‐pVDZ level,^[^ ^29^ ^]^ covering transitions up to 4.7 eV. The shape of the spectra was modeled using the linearized reflection principle approximation^[^ ^30^ ^]^ that requires only calculations of molecular properties (vibrational frequencies in the ground state, excitation energies, and forces in every electronically excited state) in the ground‐state minimum structure. However, the vibrational resolution was lost within this approach, and an unreasonable spectral width might be predicted for bound electronically excited states. Wave function stabilization was performed prior to each optimization and all calculations were done in the Gaussian package.^[^ ^31^ ^]^

## Conflict of Interest

The authors declare no conflict of interest.

## Supporting information

Supporting Information

## Data Availability

The data that support the findings of this study are available from the corresponding author upon reasonable request.
