# Supplementary material for: Gas‐Phase Electronic Structure of Phthalocyanine Ions: A Study of Symmetry and Solvation Effects
Source: Adv Sci (Weinh). 2024 Jan 15;11(12):2307816. doi: 10.1002/advs.202307816 (PMC10966524; doi:10.1002/advs.202307816)
Supplement: Supplementary file 1 — Supporting Information [file ADVS-11-2307816-s001.pdf]

ADVANCED SCIENCE
Open Access

Supporting Information

for *Adv. Sci.*, DOI 10.1002/advs.202307816

Gas-Phase Electronic Structure of Phthalocyanine Ions: A Study of Symmetry and Solvation Effects

Stefan Bergmeister, Lisa Ganner, Milan Ončák and Elisabeth Gruber**

Supporting Information
©Wiley-VCH 2021
69451 Weinheim, Germany

Gas-Phase Electronic Structure of Phthalocyanine Ions: A Study of Symmetry and Solvation Effects

Stefan Bergmeister, Lisa Ganner, Milan Ončák* and Elisabeth Gruber*

DOI:

Table of Contents

Laser power dependence measurements 3
Absorption spectra of $H_2Pc^+He_3$ and $H_2Pc^+(H_2O)_n$ 4
Photofragment – mass spectrum of the parent ion $H_2PcD^+(H_2O)_{12}$ 5
Wavelength-dependent photofragment spectra of $H_2Pc^+(H_2O)_{12}$ 6
Wavelength-dependent photofragment spectra of $H_2PcD^+(H_2O)_{12}$ 7
Isomeric structures of H_2Pc 8
Isomeric structure of H_2Pc^+ 9
Isomeric structure of $H_2Pc(H_2O)_n$, n = 1-4 10
Isomeric structure of $H_2Pc^+(H_2O)_n$, n = 1-4 11
Isomeric structure of H_2PcD^+ 12
Isomeric structure of $H_2PcD^+(H_2O)_n$, n = 1-4 13
Calculated electronic transitions in Pc^{2-} 14
Calculated electronic transitions in H_2Pc 15
Calculated electronic transitions in H_2Pc^+ 16
Calculated electronic transitions in H_2PcD^+ 17
Cartesian coordinates of all calculated isomers (in Å) as optimized at the ωB97XD/cc-pVDZ level along with the zero-point corrected electronic energy (in Hartree) 18

Laser power dependence measurements

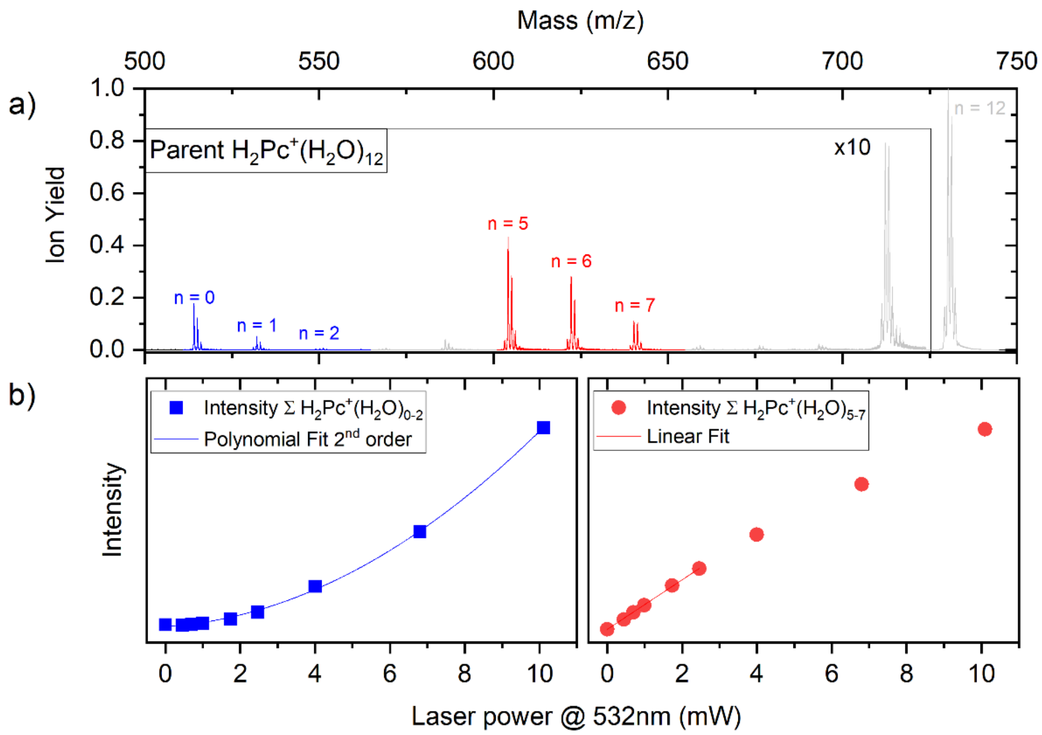

Figure S1: a) Photofragmentation spectrum of the mass-selected parent ion $H_2Pc^+(H_2O)_{12}$. A bimodal fragmentation pattern is visible, one corresponding to the loss of 5–8 water molecules and one to the loss of 11–12 water molecules. The loss of one water molecule is caused by collisions of the parent ion with residual gas between the exit of the quadrupole mass filter and the time-of-flight mass-spectrometer. b) Laser power dependence measurements of the photofragments $H_2Pc^+(H_2O)_{0\text{-}2}$ and $H_2Pc^+(H_2O)_{5\text{-}7}$. The quadratic increase of the photofragment ion yield points to a two-photon absorption process, the linear increase to a single-photon absorption process.

Absorption spectra of $H_2Pc^+He_3$ and $H_2Pc^+(H_2O)_n$

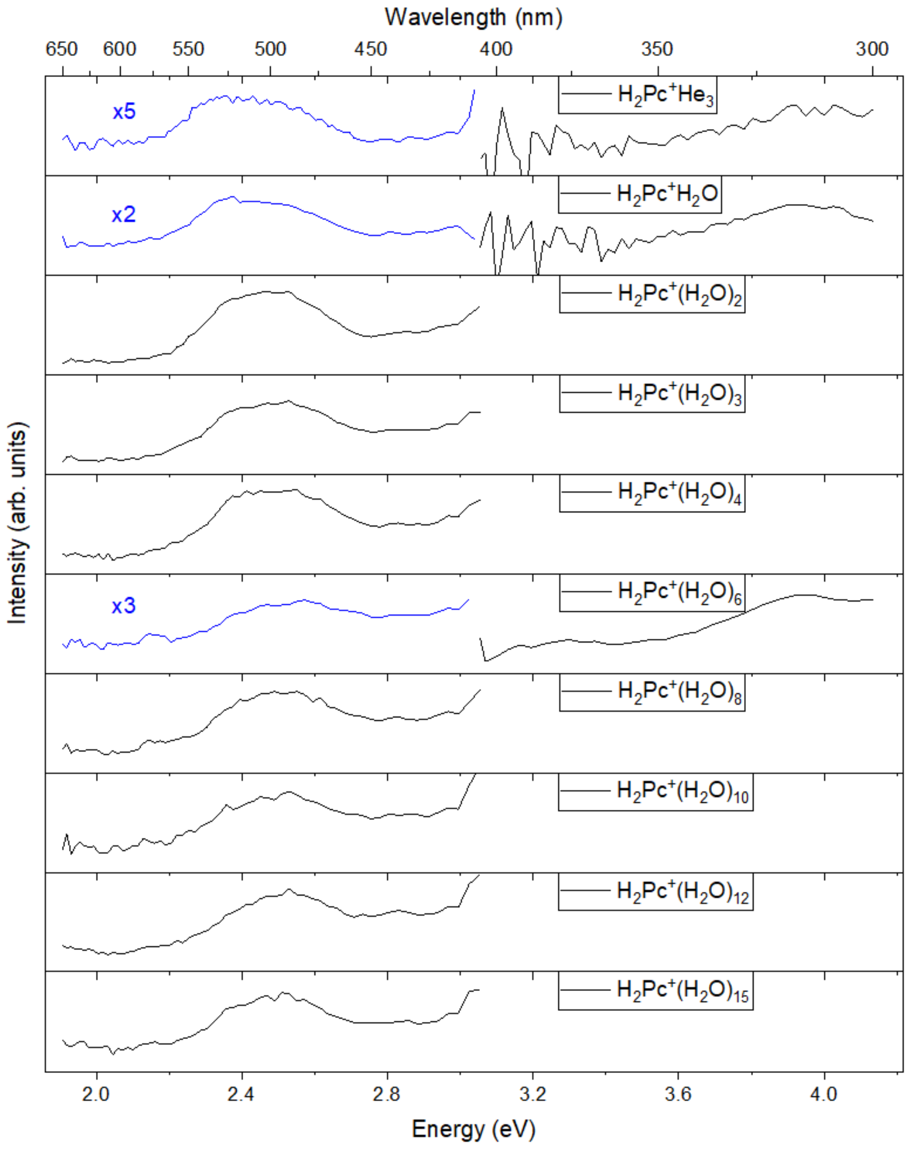

Figure S2: Absorption spectra for H_2Pc^+ attached with three He atoms and several water molecules, $(H_2O)_n$. A slight blueshift with the increase of number of water molecules attached is visible. To increase the visibililty, the spectra containing data in the UV range were adjusted in height by the corresponding factor visible in blue.

Photofragment – mass spectrum of the parent ion $H_2PcD^+(H_2O)_{12}$

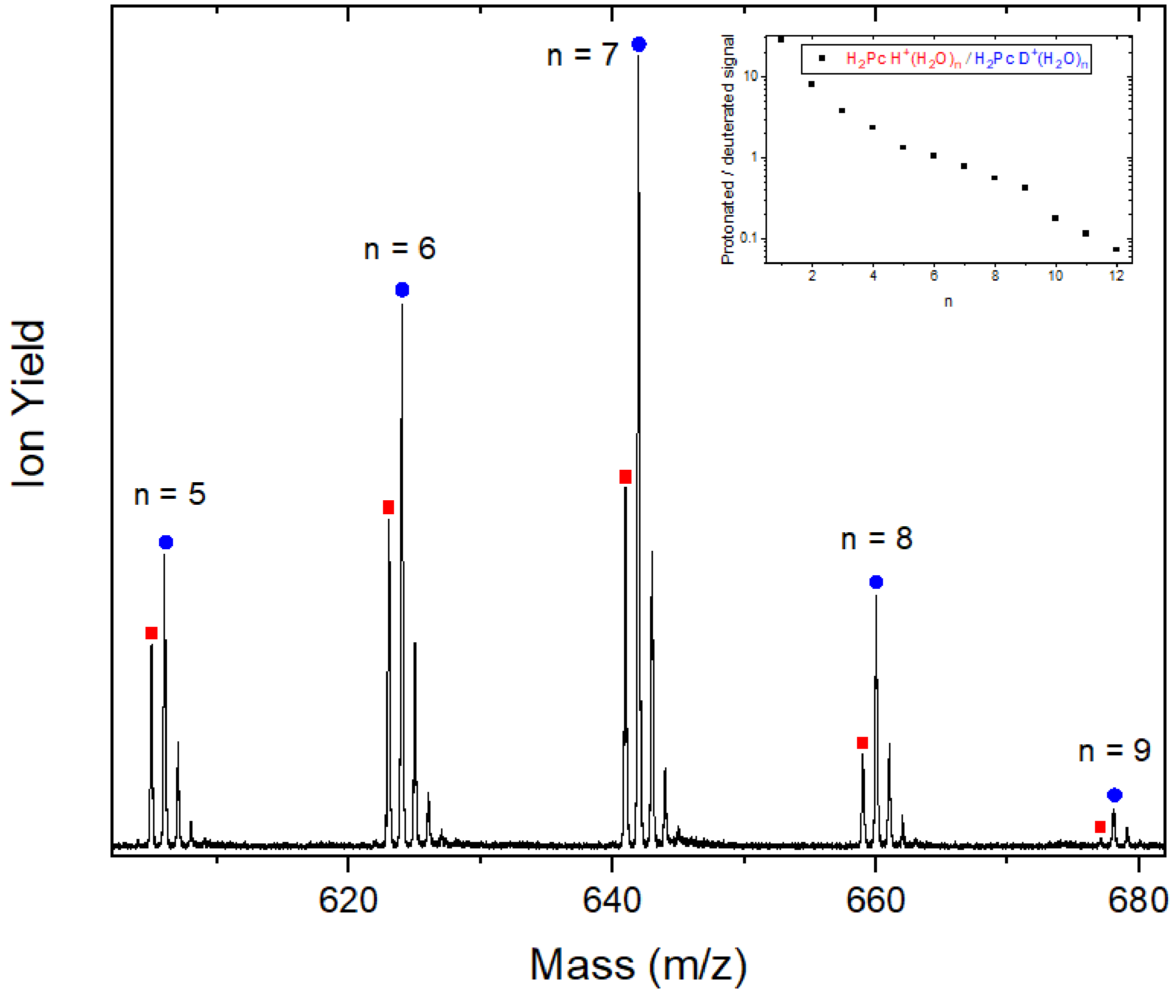

Figure S3: Mass spectrum showing the photofragments of $H_2PcD^+(H_2O)_{12}$. The peaks with the red square show photofragments which face a loss of $DHO(H_2O)_{(m-1)}$ molecules, the peaks with the blue circle show photofragments which loose only $(H_2O)_m$ molecules (with m=12-n). The inset shows the ratio of the neighbouring protonated/deuterated peaks in dependence of the number of remaining water molecules attached.

Wavelength-dependent photofragment spectra of $H_2Pc^+(H_2O)_{12}$

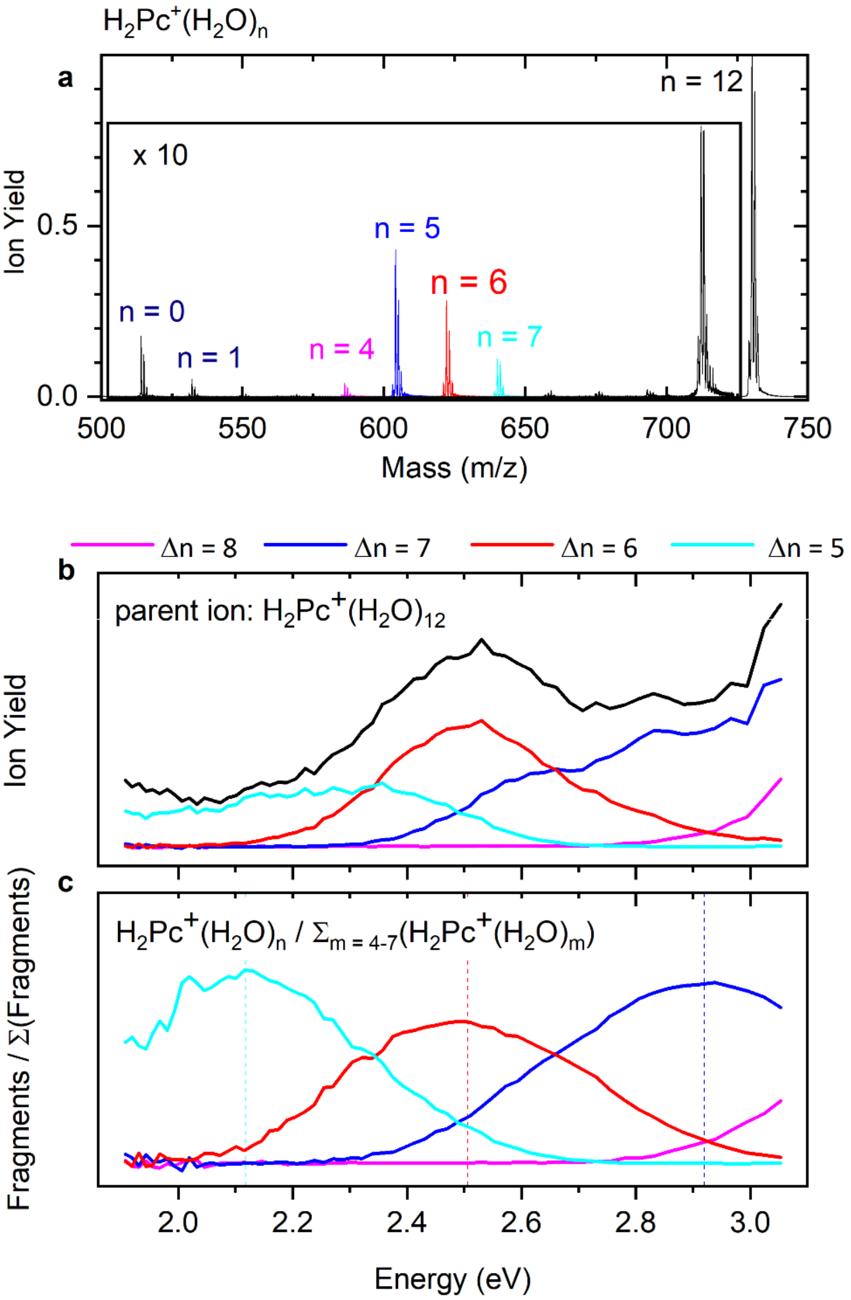

Figure S4: a) Mass spectrum of the precursor $H_2Pc^+(H_2O)_{12}$ when photofragmentation occurs. The fragments are plotted in different colors and are multiplied by a factor of 10 due to visibility reasons. b) shows the wavelength dependence of each photofragment for the precursor $H_2Pc^+(H_2O)_{12}$. c) shows the wavelength dependence of each photofragment for the precursor $H_2Pc^+(H_2O)_{12}$ relative to the sum of all photofragments. By dividing the mean value of these curves by the corresponding number of lost water molecules, we deduce a binding energy of 0.42(4)eV per water molecule.

Wavelength-dependent photofragment spectra of $H_2PcD^+(H_2O)_{12}$

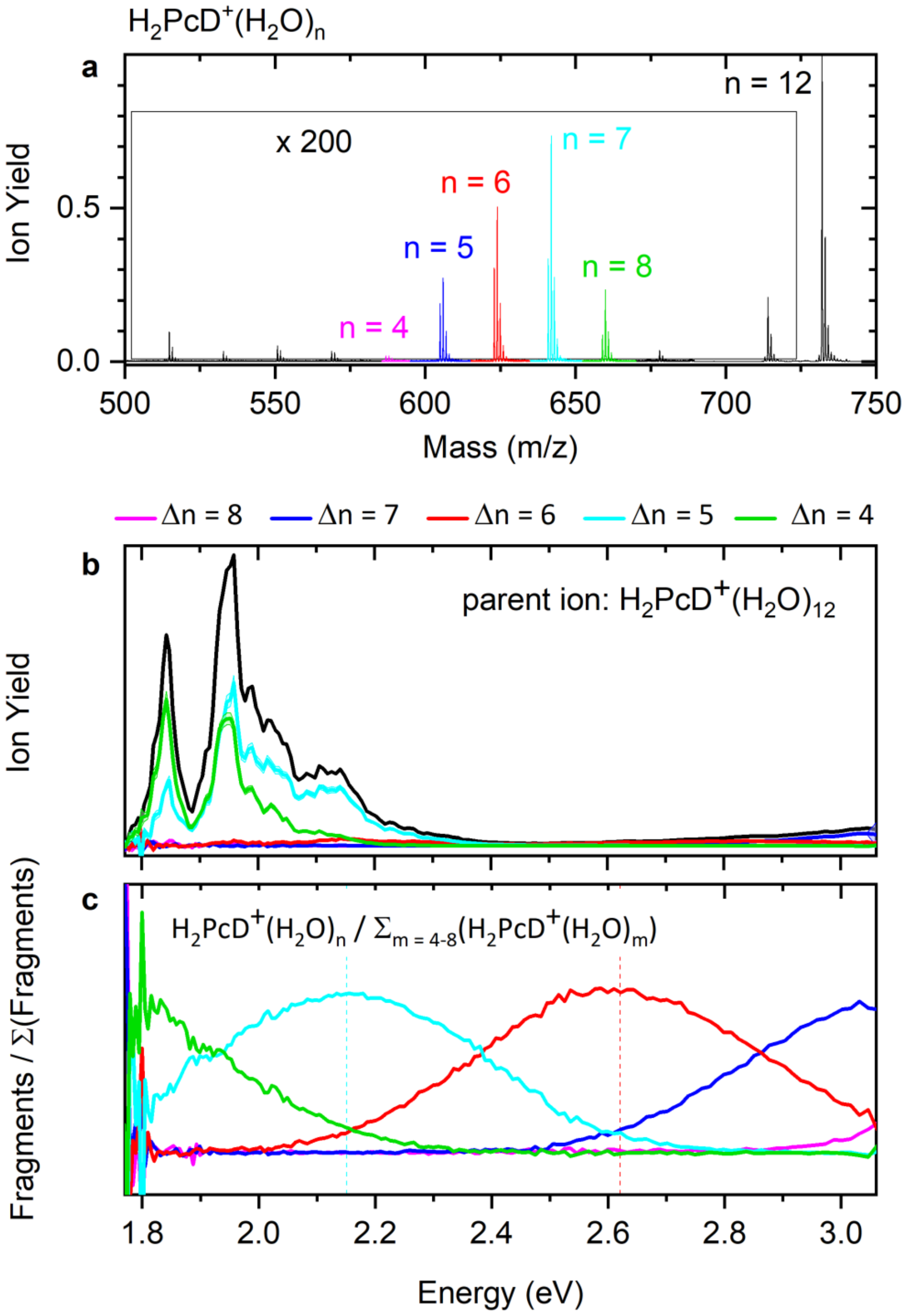

Figure S5: a) Mass spectrum of the precursor $H_2PcD^+(H_2O)_{12}$ when photofragmentation occur. The fragments are plotted in different colours and are multiplied by a factor of 200 due to visibility reasons. b) shows the wavelength dependence of each photofragment for the precursor $H_2PcD^+(H_2O)_{12}$. c) shows the wavelength dependence of each photofragment for the precursor $H_2PcD^+(H_2O)_{12}$ relative to the sum of all photofragments. By dividing the mean value of these curves by the corresponding number of lost water molecules, we deduce a binding energy of 0.43(5)eV per water molecule.

Isomeric structures of H_2Pc

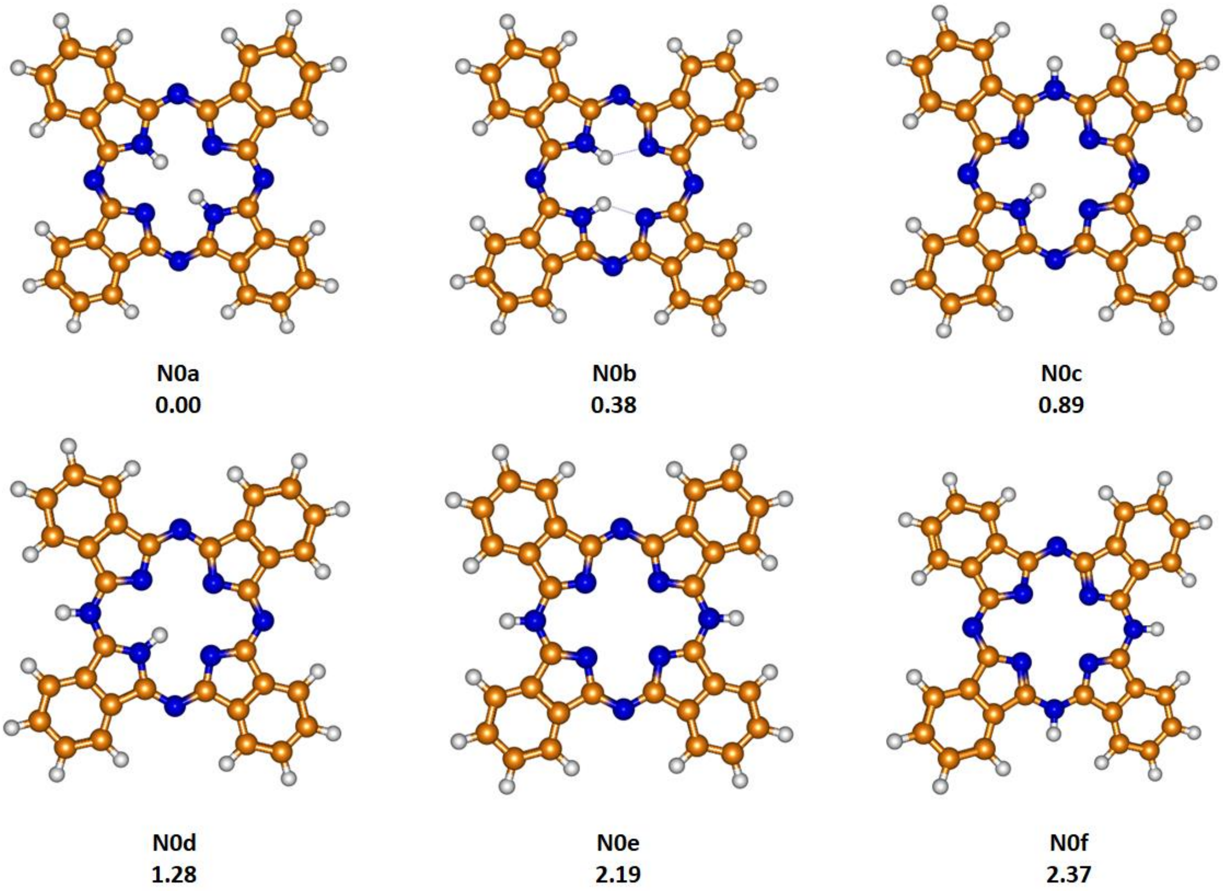

Figure S6: Isomers of H_2Pc as optimized at the ωB97XD/aug-cc-pVDZ level of theory along with their relative energies (in eV). Position of two hydrogen atoms was chosen as to include isomers with two N–H bonds in the inner cycle (a,b), combined isomers with hydrogens inside and outside (c,d) and only outside (e,f).

Isomeric structure of H_2Pc^+

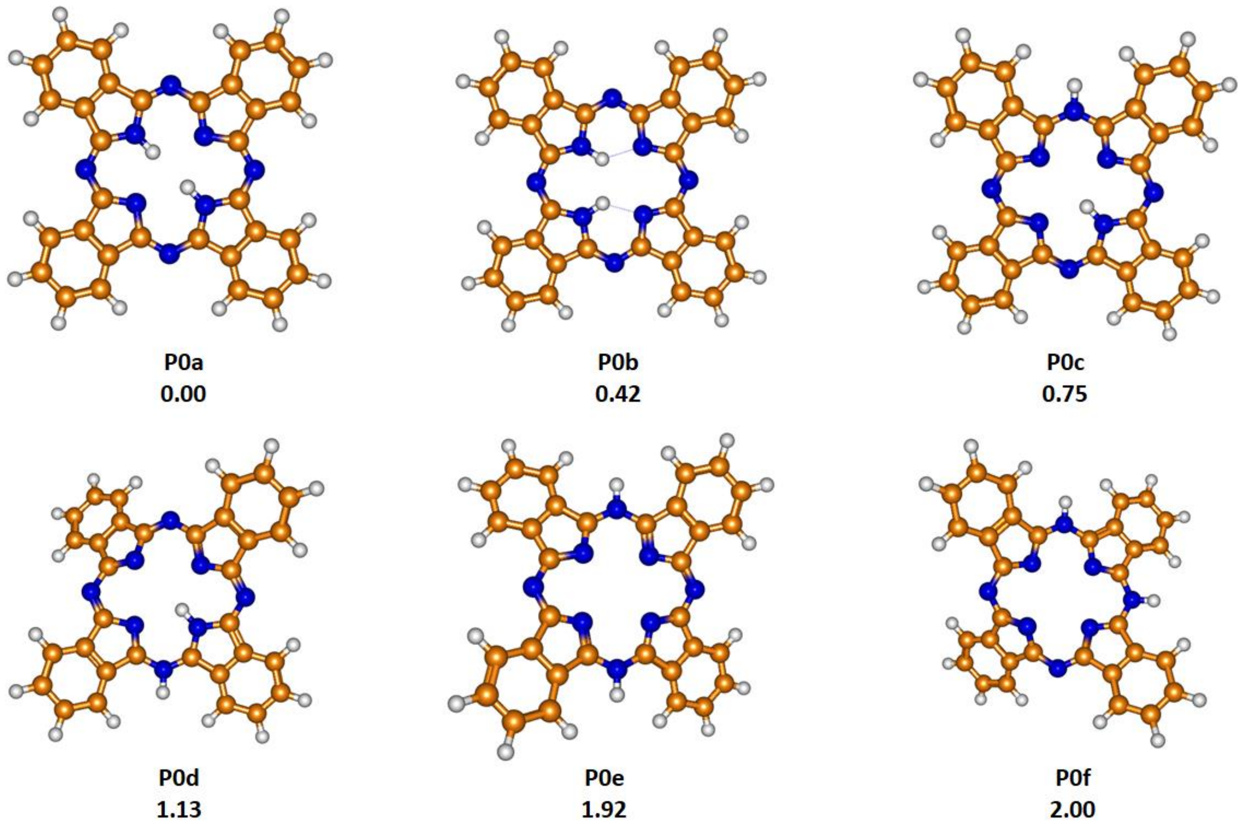

Figure S7: Isomers of H_2Pc^+ as optimized at the ωB97XD/aug-cc-pVDZ level of theory along with their relative energies (in eV). The same structure search strategy was employed as described for Figure S5.

Isomeric structure of $H_2Pc(H_2O)_n$, *n* = 1-4

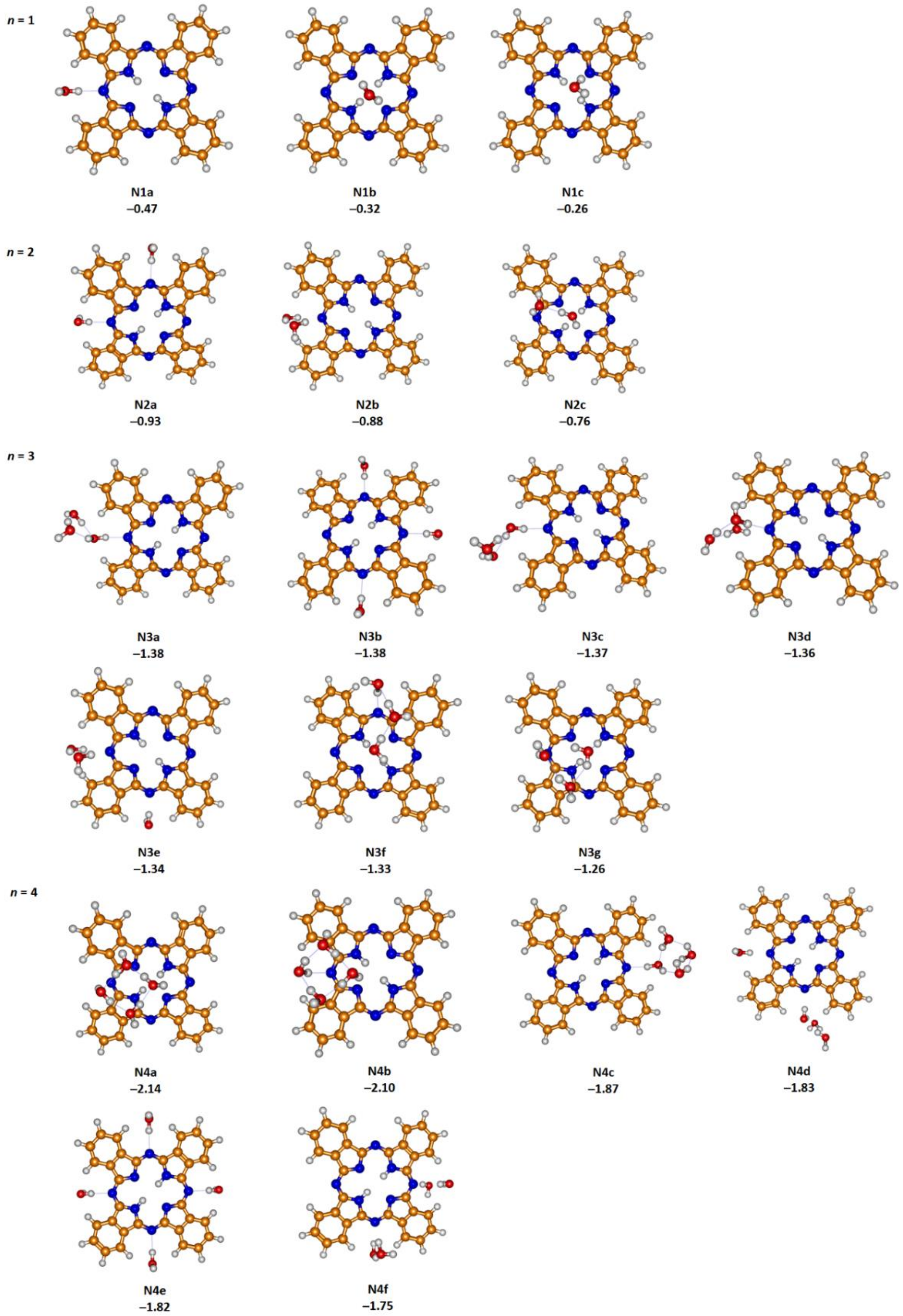

Figure S8: Isomers of $H_2Pc(H_2O)_n$, *n* = 1–4, as optimized at the ωB97XD/aug-cc-pVDZ level of theory along with the hydration energy corresponding to the H_2Pc + *n* H_2O → $H_2Pc(H_2O)_n$ reaction (in eV). For *n* = 1, the water molecule was positioned both outside the ring and in the middle of the molecule, above the ring. For *n* = 2, we considered positioning water molecules outside the ring without a hydrogen bond between them (a) as well as forming a water dimer outside the inner cycle (b) or on top of it (c). For *n* = 3, 4, independent water molecules connected to outside nitrogen atoms as well as water trimers at various positions were considered.

Isomeric structure of $H_2Pc^+(H_2O)_n$, n = 1-4

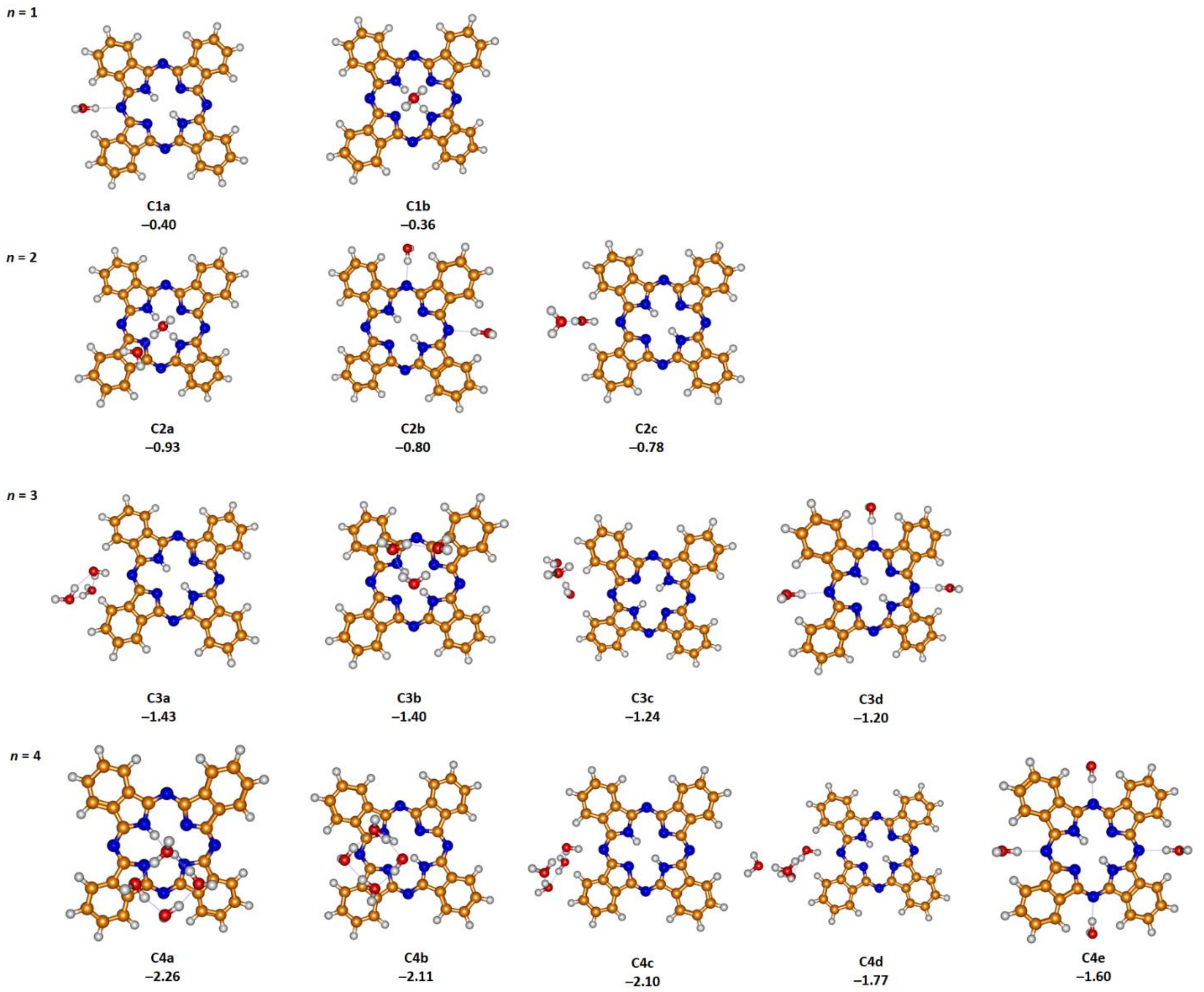

Figure S9: Isomers of $H_2Pc^+(H_2O)_n$, n = 1–4, as optimized at the ωB97XD/aug-cc-pVDZ level of theory along with the hydration energy corresponding to the $H_2Pc^+ + n\ H_2O \rightarrow H_2Pc^+(H_2O)_n$ reaction (in eV). The same structure search strategy was employed as described for Figure S7.

Isomeric structure of H_2PcD^+

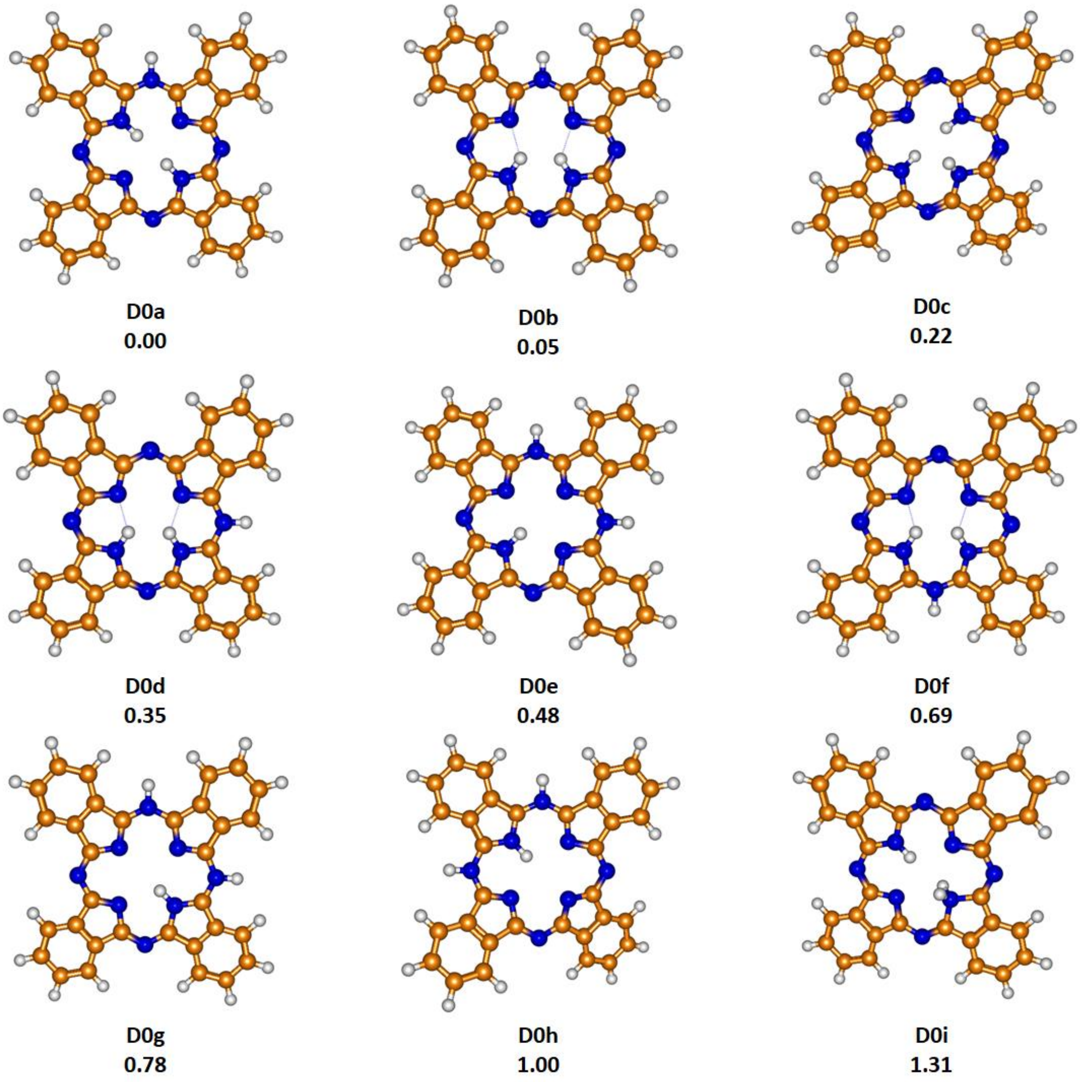

Figure S10: Isomers of H_2PcD^+ as optimized at the ωB97XD/aug-cc-pVDZ level of theory along with their relative energies (in eV). Position of three hydrogen atoms was chosen as to include isomers with three N–H bonds in the inner cycle (c,i), combined isomers with two hydrogens inside and one outside (a,b,d,f), and one inside and two outside (e,g,h).

Isomeric structure of $H_2PcD^+(H_2O)_n$, n = 1-4

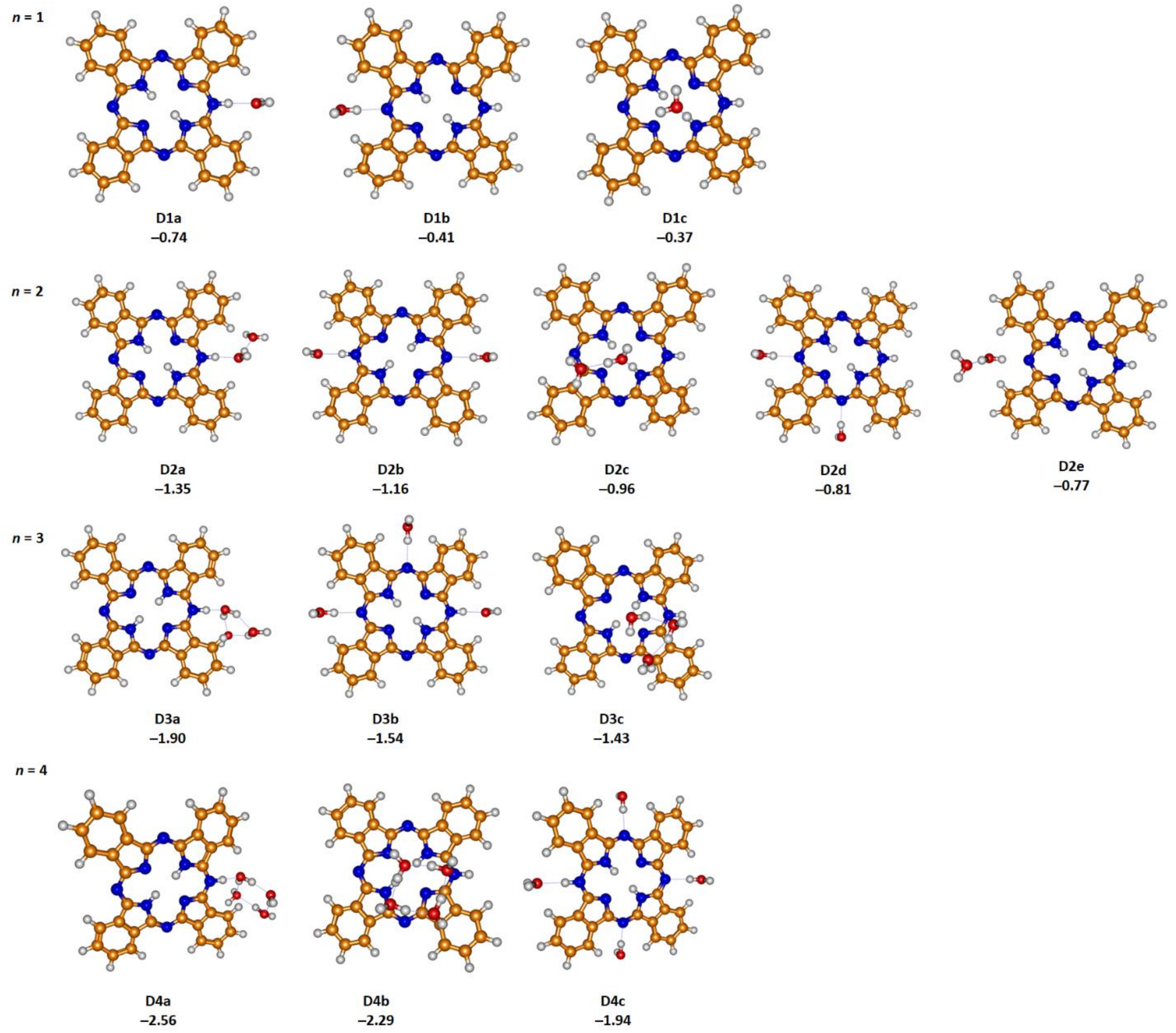

Figure S11: Isomers of $H_2PcD^+(H_2O)_n$, n = 1–4, as optimized at the ωB97XD/aug-cc-pVDZ level of theory along with the hydration energy corresponding to the $H_2PcD^+ + n\ H_2O \rightarrow H_2PcD^+(H_2O)_n$ reaction (in eV). The same structure search strategy was employed as described for Figure S7.

Calculated electronic transitions in Pc^{2-}

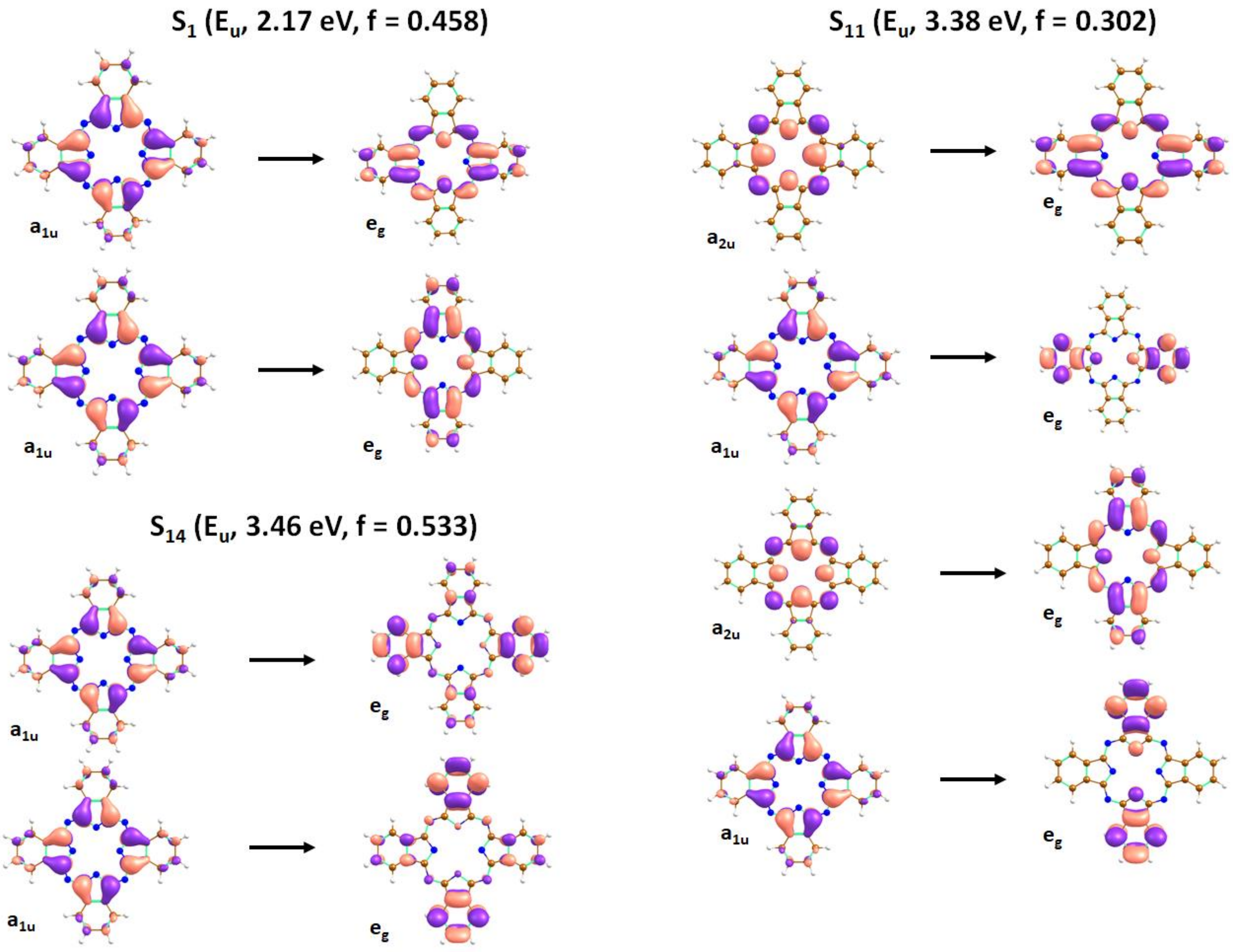

Figure S12: Character of selected electronic transitions in Pc^{2-} as calculated at the TD-BMK/aug-cc-pVDZ// ωB97XD/aug-cc-pVDZ level. The orbitals with the highest contribution within the natural transition orbitals analysis are shown.

Calculated electronic transitions in H_2Pc

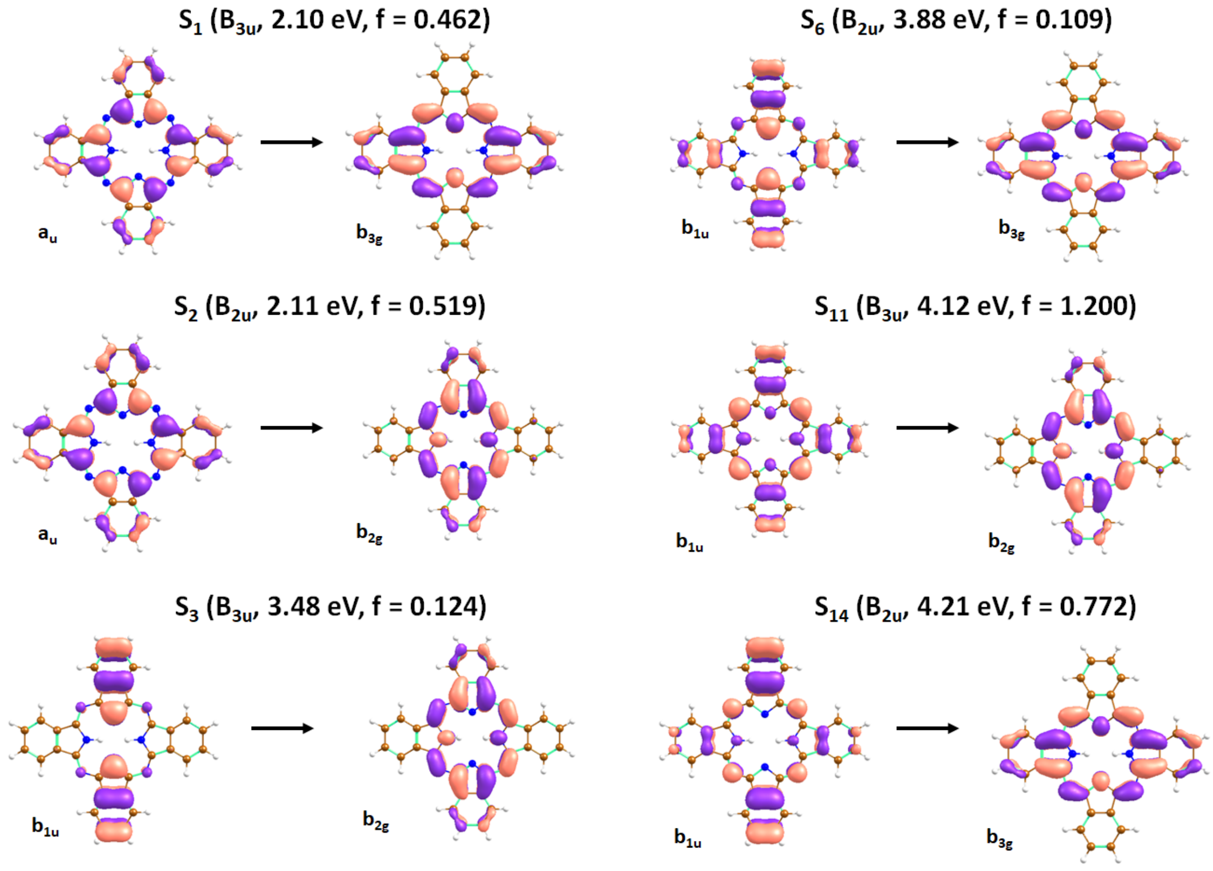

Figure S13: Character of selected electronic transitions in H_2Pc as calculated at the TD-BMK/aug-cc-pVDZ// ωB97XD/aug-cc-pVDZ level. The orbitals with the highest contribution within the natural transition orbitals analysis are shown.

Calculated electronic transitions in H_2Pc^+

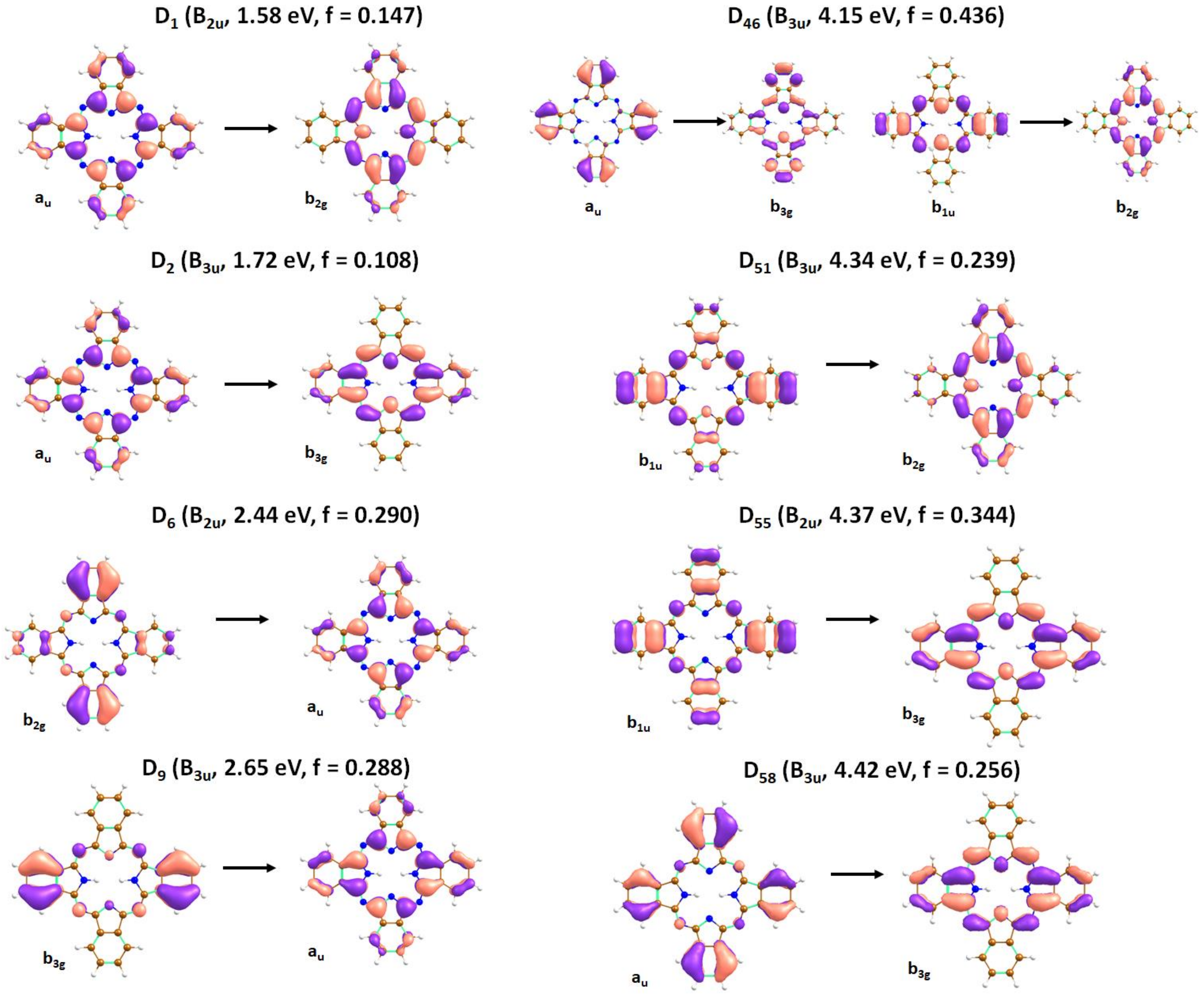

Figure S14: Character of selected electronic transitions in H_2Pc^+ as calculated at the TD-BMK/aug-cc-pVDZ// ωB97XD/aug-cc-pVDZ level. The orbitals with the highest contribution within the natural transition orbitals analysis are shown.

Calculated electronic transitions in H_2PcD^+

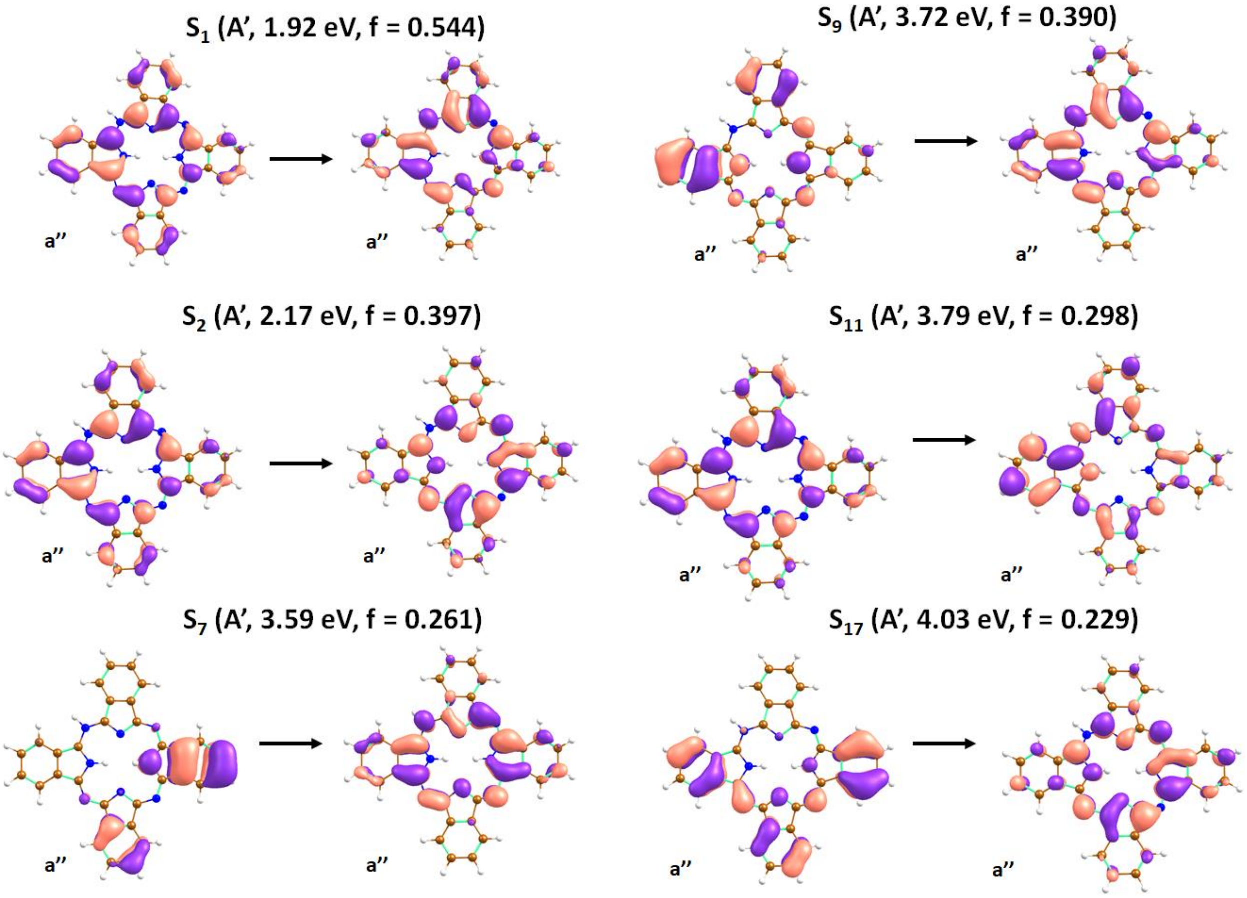

Figure S15: Character of selected electronic transitions in H_2PcD^+ as calculated at the TD-BMK/aug-cc-pVDZ// ωB97XD/aug-cc-pVDZ level. The orbitals with the highest contribution within the natural transition orbitals analysis are shown.

Cartesian coordinates of all calculated isomers (in Å) as optimized at the ωB97XD/cc-pVDZ level along with the zero-point corrected electronic energy (in Hartree)

Pc(2-)
E = -1666.232021
N 0.000000 1.961557 0.000000
C 1.093233 2.745641 0.000000
C 0.703437 4.171608 0.000000
C -0.703437 4.171608 -0.000000
C -1.093233 2.745641 -0.000000
C -1.418045 5.366838 0.000000
C -0.702724 6.566510 0.000000
C 0.702724 6.566510 0.000000
C 1.418045 5.366838 0.000000
N 2.379041 2.379041 -0.000000
C 2.745641 1.093233 -0.000000
C 4.171608 0.703437 -0.000000
C 4.171608 -0.703437 0.000000
C 2.745641 -1.093233 0.000000
N 1.961557 -0.000000 0.000000
C 5.366838 1.418045 0.000000
C 6.566510 0.702724 0.000000
C 6.566510 -0.702724 -0.000000
C 5.366838 -1.418045 -0.000000
N 2.379041 -2.379041 -0.000000
C 1.093233 -2.745641 -0.000000
C 0.703437 -4.171608 -0.000000
C -0.703437 -4.171608 0.000000
C -1.093233 -2.745641 0.000000
N -0.000000 -1.961557 0.000000
C 1.418045 -5.366838 0.000000
C 0.702724 -6.566510 0.000000
C -0.702724 -6.566510 -0.000000
C -1.418045 -5.366838 -0.000000
N -2.379041 -2.379041 -0.000000
C -2.745641 -1.093233 -0.000000
C -4.171608 -0.703437 -0.000000
C -4.171608 0.703437 -0.000000
C -2.745641 1.093233 -0.000000
N -1.961557 0.000000 0.000000
C -5.366838 -1.418045 -0.000000
C -6.566510 -0.702724 -0.000000
C -6.566510 0.702724 -0.000000
C -5.366838 1.418045 -0.000000
N -2.379041 2.379041 -0.000000
H -5.347656 2.510254 0.000000
H -7.520198 1.239356 0.000000
H -7.520198 -1.239356 -0.000000
H -5.347656 -2.510254 -0.000000
H 2.510254 -5.347656 -0.000000
H 1.239356 -7.520198 -0.000000
H -1.239356 -7.520198 -0.000000
H -2.510254 -5.347656 -0.000000
H 5.347656 2.510254 0.000000
H 7.520198 1.239356 0.000000
H 7.520198 -1.239356 0.000000
H 5.347656 -2.510254 0.000000
H -2.510254 5.347656 0.000000
H -1.239356 7.520198 0.000000
H 1.239356 7.520198 0.000000
H 2.510254 5.347656 0.000000

N0a
E = -1667.433958
C 0.000000 4.156161 0.698801
C 0.000000 5.344231 1.424291
C 0.000000 6.536598 0.702534
C 0.000000 6.536598 -0.702534
C 0.000000 5.344231 -1.424291
C 0.000000 4.156161 -0.698801
H 0.000000 5.332186 2.514686
H 0.000000 7.489491 1.235025
H 0.000000 7.489491 -1.235025
H 0.000000 5.332186 -2.514686
C 0.000000 2.744330 -1.090890
C -0.000000 2.744330 1.090890
N 0.000000 1.933125 0.000000
N 0.000000 2.388959 -2.378118
C 0.000000 1.139055 -2.777237
C 0.000000 0.702373 -4.164416
C -0.000000 -0.702373 -4.164416
C 0.000000 -1.425222 -5.358826
C 0.000000 -0.704538 -6.546116
C 0.000000 0.704538 -6.546116
C 0.000000 1.425222 -5.358826
C -0.000000 -1.139055 -2.777237
H 0.000000 -2.515259 -5.344685
H 0.000000 -1.235983 -7.499313
H 0.000000 1.235983 -7.499313
H 0.000000 2.515259 -5.344685
N 0.000000 0.000000 -2.013994
H 0.000000 0.000000 -0.998336
N -0.000000 -2.388959 -2.378118
C -0.000000 -2.744330 -1.090890
C -0.000000 -4.156161 -0.698801
C -0.000000 -4.156161 0.698801
C -0.000000 -5.344231 1.424291
C -0.000000 -6.536598 0.702534
C -0.000000 -6.536598 -0.702534
C -0.000000 -5.344231 -1.424291
C -0.000000 -2.744330 1.090890
H -0.000000 -5.332186 2.514686
H -0.000000 -7.489491 1.235025
H -0.000000 -7.489491 -1.235025
H -0.000000 -5.332186 -2.514686
N -0.000000 -1.933125 0.000000
N -0.000000 -2.388959 2.378118
C -0.000000 -1.139055 2.777237
C -0.000000 -0.702373 4.164416
C -0.000000 0.702373 4.164416
C 0.000000 1.425222 5.358826
C 0.000000 0.704538 6.546116
C -0.000000 -0.704538 6.546116
C -0.000000 -1.425222 5.358826
C -0.000000 1.139055 2.777237
H 0.000000 2.515259 5.344685
H 0.000000 1.235983 7.499313
H -0.000000 -1.235983 7.499313
H -0.000000 -2.515259 5.344685
N -0.000000 0.000000 2.013994
N -0.000000 2.388959 2.378118
H -0.000000 0.000000 0.998336

N0b
E = -1667.419919
6 0.000000 4.797010 -2.882962
6 0.000000 3.468700 -2.477141
6 0.000000 2.422204 -3.400817
6 0.000000 2.662677 -4.768345
6 0.000000 3.995442 -5.185363
6 0.000000 5.046231 -4.256917
6 0.000000 2.817831 -1.157808
7 0.000000 1.459754 -1.284157
6 0.000000 1.176228 -2.604376
7 0.000000 0.000000 -3.219004
6 -0.000000 -1.176228 -2.604376
6 -0.000000 -2.422204 -3.400817
6 -0.000000 -3.468700 -2.477141
6 -0.000000 -2.817831 -1.157808
7 -0.000000 -1.459754 -1.284157
6 -0.000000 -2.662677 -4.768345

6 -0.000000 -3.995442 -5.185363
6 -0.000000 -5.046231 -4.256917
6 -0.000000 -4.797010 -2.882962
7 -0.000000 -3.501564 -0.024576
6 -0.000000 -2.864065 1.143286
6 -0.000000 -3.477836 2.449099
6 -0.000000 -2.435455 3.394780
6 -0.000000 -1.181417 2.672397
7 -0.000000 -1.512544 1.330678
6 -0.000000 -4.820101 2.846385
6 -0.000000 -5.087249 4.205579
6 -0.000000 -4.041917 5.155777
6 -0.000000 -2.711891 4.767260
7 -0.000000 0.000000 3.249987
6 0.000000 1.181417 2.672397
7 0.000000 1.512544 1.330678
6 0.000000 2.864065 1.143286
6 0.000000 3.477836 2.449099
6 0.000000 2.435455 3.394780
6 0.000000 4.820101 2.846385
6 0.000000 5.087249 4.205579
6 0.000000 4.041917 5.155777
6 0.000000 2.711891 4.767260
7 0.000000 3.501564 -0.024576
1 0.000000 5.606063 -2.151871
1 0.000000 6.076380 -4.617810
1 0.000000 4.226994 -6.252042
1 0.000000 1.835682 -5.478953
1 -0.000000 -5.606063 -2.151871
1 -0.000000 -6.076380 -4.617810
1 -0.000000 -4.226994 -6.252042
1 -0.000000 -1.835682 -5.478953
1 -0.000000 -1.899223 5.493883
1 -0.000000 -4.292269 6.218152
1 -0.000000 -6.121438 4.553801
1 -0.000000 -5.615495 2.100864
1 0.000000 5.615495 2.100864
1 0.000000 6.121438 4.553801
1 0.000000 4.292269 6.218152
1 0.000000 1.899223 5.493883
1 0.000000 0.946491 0.466927
1 -0.000000 -0.946491 0.466927

N0c
E = -1667.401360
N 0.028419 1.971457 -0.000000
C 1.180804 2.734776 -0.000000
C 0.763144 4.126589 -0.000000
C -0.637221 4.145753 -0.000000
C -1.094460 2.763742 -0.000000
C -1.346995 5.347526 -0.000000
C -0.612466 6.526467 -0.000000
C 0.796657 6.507908 -0.000000
C 1.502203 5.311610 -0.000000
N 2.442592 2.362150 -0.000000
C 2.820351 1.088090 -0.000000
C 4.217771 0.659978 -0.000000
C 4.181639 -0.746087 0.000000
C 2.752482 -1.064733 0.000000
N 1.984044 0.010052 -0.000000
C 5.428298 1.347952 -0.000000
C 6.602769 0.599328 -0.000000
C 6.568877 -0.804604 0.000000
C 5.358556 -1.494786 0.000000
N 2.276651 -2.344993 0.000000
C 0.982355 -2.761673 0.000000
C 0.594359 -4.182855 0.000000
C -0.807498 -4.141141 0.000000
C -1.168204 -2.709693 0.000000
N -0.040130 -1.935807 0.000000
C 1.279746 -5.393778 0.000000
C 0.523109 -6.567444 0.000000
C -0.877372 -6.524171 0.000000
C -1.561437 -5.307199 0.000000
N -2.422609 -2.307182 0.000000
C -2.772163 -1.016975 0.000000
C -4.192341 -0.621016 0.000000
C -4.191106 0.778004 -0.000000
C -2.768793 1.154972 -0.000000
N -1.969266 0.072932 -0.000000
C -5.380446 -1.341972 0.000000
C -6.574055 -0.617465 0.000000
C -6.571885 0.785128 -0.000000
C -5.375300 1.504880 -0.000000
N -2.366969 2.430309 -0.000000
H -5.361882 2.595423 -0.000000
H -7.523519 1.320016 -0.000000
H -7.527747 -1.148791 0.000000
H -5.370234 -2.432520 0.000000
H 2.371178 -5.447825 0.000000
H 1.029967 -7.533761 0.000000
H -1.438941 -7.459988 0.000000
H -2.650780 -5.259106 0.000000
H 5.443410 2.438297 -0.000000
H 7.568342 1.107992 -0.000000
H 7.506363 -1.362952 0.000000
H 5.354162 -2.587674 0.000000
H -2.437126 5.343518 -0.000000
H -1.131555 7.486520 -0.000000
H 1.340182 7.454464 -0.000000
H 2.591967 5.282873 -0.000000
H 0.000000 0.942982 -0.000000
H 2.978812 -3.074548 0.000000

N0d
E = -1667.386949
N 0.076479 1.981822 -0.000000
C 1.225847 2.702556 -0.000000
C 0.909520 4.086077 -0.000000
C -0.506226 4.159404 0.000000
C -1.014578 2.827361 0.000000
C -1.160861 5.405165 0.000000
C -0.388841 6.546953 0.000000
C 1.029630 6.475741 -0.000000
C 1.687474 5.264767 -0.000000
N 2.491828 2.180844 -0.000000
C 2.863151 0.891796 -0.000000
C 4.258743 0.426444 -0.000000
C 4.132420 -0.967922 -0.000000
C 2.677125 -1.255902 -0.000000
N 1.971707 -0.087459 -0.000000
C 5.506412 1.040076 -0.000000
C 6.632141 0.212528 -0.000000
C 6.504632 -1.181801 -0.000000
C 5.247662 -1.791798 -0.000000
N 2.237318 -2.492950 -0.000000
C 0.919013 -2.773535 -0.000000
C 0.452103 -4.182530 -0.000000
C -0.940936 -4.100216 0.000000
C -1.245157 -2.645607 0.000000
N -0.091127 -1.912426 0.000000
C 1.107513 -5.403755 -0.000000
C 0.315273 -6.557646 0.000000
C -1.081578 -6.475231 0.000000
C -1.733614 -5.236549 0.000000
N -2.478883 -2.207578 0.000000
C -2.782162 -0.889445 0.000000
C -4.200584 -0.469351 0.000000
C -4.181083 0.927834 0.000000
C -2.752221 1.286031 0.000000
N -1.965533 0.159040 0.000000
C -5.394952 -1.178286 0.000000
C -6.580975 -0.440862 0.000000
C -6.561425 0.961087 0.000000
C -5.356662 1.667751 0.000000
N -2.318162 2.519214 0.000000

H -5.332144 2.758119 0.000000
H -7.506647 1.507407 0.000000
H -7.540739 -0.961036 0.000000
H -5.394027 -2.268808 0.000000
H 2.196894 -5.454939 -0.000000
H 0.792203 -7.539618 -0.000000
H -1.670370 -7.394504 0.000000
H -2.821395 -5.158903 0.000000
H 5.625004 2.126061 -0.000000
H 7.627150 0.660153 -0.000000
H 7.404809 -1.798667 -0.000000
H 5.131194 -2.875834 -0.000000
H -2.250386 5.443122 0.000000
H -0.868307 7.527117 0.000000
H 1.607244 7.401553 -0.000000
H 2.779935 5.235239 -0.000000
H 0.000000 0.951979 -0.000000
H 3.233356 2.871195 -0.000000

N0e
E = -1667.353660
N -1.972055 0.030354 -0.000000
C -2.767937 1.141552 -0.000000
C -4.190216 0.754525 -0.000000
C -4.202236 -0.650233 0.000000
C -2.772282 -1.009248 0.000000
C -5.399623 -1.359084 0.000000
C -6.589858 -0.628291 0.000000
C -6.576291 0.772724 -0.000000
C -5.373768 1.481868 -0.000000
N -2.384221 2.412306 -0.000000
C -1.109042 2.781109 -0.000000
C -0.705404 4.198750 -0.000000
C 0.699402 4.194328 -0.000000
C 1.041655 2.760272 -0.000000
N -0.007247 1.972268 -0.000000
C -1.418844 5.390732 -0.000000
C -0.695677 6.584871 -0.000000
C 0.705405 6.582045 -0.000000
C 1.422220 5.383342 -0.000000
N 2.330840 2.303693 -0.000000
C 2.772281 1.009245 -0.000000
C 4.202236 0.650232 -0.000000
C 4.190217 -0.754526 0.000000
C 2.767936 -1.141555 0.000000
N 1.972055 -0.030352 0.000000
C 5.399621 1.359086 -0.000000
C 6.589857 0.628293 -0.000000
C 6.576292 -0.772721 0.000000
C 5.373770 -1.481867 0.000000
N 2.384225 -2.412305 0.000000
C 1.109037 -2.781107 0.000000
C 0.705405 -4.198748 0.000000
C -0.699403 -4.194328 0.000000
C -1.041656 -2.760276 0.000000
N 0.007249 -1.972270 0.000000
C 1.418844 -5.390732 0.000000
C 0.695678 -6.584870 0.000000
C -0.705404 -6.582046 0.000000
C -1.422220 -5.383344 0.000000
N -2.330841 -2.303692 0.000000
H -2.514969 -5.401409 0.000000
H -1.244160 -7.531066 0.000000
H 1.226451 -7.538647 0.000000
H 2.509325 -5.379924 0.000000
H 5.430469 2.451548 -0.000000
H 7.545113 1.155918 -0.000000
H 7.523794 -1.314615 0.000000
H 5.350192 -2.572147 0.000000
H -2.509325 5.379923 -0.000000
H -1.226449 7.538648 -0.000000
H 1.244161 7.531066 -0.000000
H 2.514969 5.401406 -0.000000
H -5.430472 -2.451547 0.000000
H -7.545114 -1.155915 0.000000
H -7.523792 1.314619 -0.000000
H -5.350188 2.572147 -0.000000
H 3.050771 3.015237 -0.000000
H -3.050771 -3.015238 0.000000

N0f
E = -1667.346852
N -1.495559 1.276922 0.040653
C -1.422857 2.586804 -0.025801
C -2.734167 3.244445 -0.208367
C -3.636137 2.172178 -0.248929
C -2.815752 0.948301 -0.098919
C -4.997558 2.382644 -0.411508
C -5.443035 3.701645 -0.530521
C -4.543910 4.773786 -0.491071
C -3.172241 4.558151 -0.330404
N -0.258775 3.283364 0.061085
C 1.021414 2.797832 0.128351
C 2.211882 3.624011 0.245341
C 3.281796 2.693628 0.245398
C 2.628949 1.400005 0.128398
N 1.302586 1.497928 0.064893
C 2.461933 4.994693 0.346111
C 3.782562 5.420586 0.443104
C 4.843090 4.498286 0.443145
C 4.604506 3.131370 0.346191
N 3.287522 0.199625 0.061137
C 2.759328 -1.049878 -0.025768
C 3.592675 -2.257183 -0.208346
C 2.656027 -3.299268 -0.248962
C 1.330102 -2.656717 -0.099011
N 1.472250 -1.303718 0.040634
C 4.954469 -2.508782 -0.330390
C 5.358331 -3.837294 -0.491038
C 4.421377 -4.876511 -0.530481
C 3.053338 -4.618322 -0.411506
N 0.219154 -3.362550 -0.080982
C -0.996703 -2.795032 -0.027889
C -2.209774 -3.607647 0.208244
C -3.265902 -2.689314 0.208357
C -2.629754 -1.375115 -0.027793
N -1.298896 -1.493506 -0.181262
C -2.429898 -4.962291 0.412894
C -3.749722 -5.379137 0.613997
C -4.806490 -4.460201 0.614103
C -4.576857 -3.095305 0.413119
N -3.360596 -0.249846 -0.080841
H -5.391946 -2.370481 0.414493
H -5.825342 -4.818216 0.774710
H -3.962824 -6.437829 0.774545
H -1.598985 -5.668921 0.414154
H 5.700926 -1.710898 -0.306286
H 6.420437 -4.068021 -0.588692
H 4.769122 -5.903265 -0.657265
H 2.311537 -5.417004 -0.442899
H 1.651382 5.727443 0.353280
H 4.000860 6.486572 0.523057
H 5.868476 4.862371 0.523111
H 5.442605 2.430290 0.353402
H -5.685602 1.537206 -0.442921
H -6.508105 3.903522 -0.657359
H -4.919790 5.793604 -0.588725
H -2.485647 5.408099 -0.306287
H 4.298312 0.253888 0.041124
H -0.345361 4.291903 0.041049

N1a
E = -1743.829237
C 2.851795 4.858594 -0.008423
C 2.570860 3.495218 -0.004762
C 3.592193 2.541359 0.003865

C 4.933484 2.913390 0.009165
C 5.219979 4.277280 0.005549
C 4.193276 5.236483 -0.003147
C 1.321037 2.730349 -0.008014
N 1.563476 1.393745 -0.002231
C 2.914482 1.242748 0.005035
N 3.610294 0.102130 0.012400
C 3.034625 -1.076294 0.011411
C 3.730104 -2.353519 0.018047
C 2.754096 -3.363128 0.012254
C 1.452220 -2.713591 0.002301
N 1.694063 -1.364385 0.003044
C 5.091091 -2.663368 0.027661
C 5.443448 -4.006939 0.031410
C 4.464216 -5.019860 0.025573
C 3.109642 -4.712900 0.015892
N 0.300546 -3.338921 -0.006852
C -0.881652 -2.719225 -0.015061
C -2.122988 -3.490779 -0.031281
C -3.159163 -2.550648 -0.035252
C -2.486754 -1.242334 -0.020112
N -1.135829 -1.382908 -0.010010
C -2.371562 -4.859808 -0.045913
C -3.702547 -5.269075 -0.066318
C -4.745425 -4.329384 -0.072208
C -4.493915 -2.958117 -0.056361
N -3.176286 -0.090422 -0.017183
C -2.607782 1.098173 -0.015789
N -1.265998 1.374999 -0.014071
C -1.012572 2.724090 -0.016927
C -2.307121 3.381751 -0.021731
C -3.298330 2.384874 -0.019914
C -2.632453 4.738416 -0.029174
C -3.979172 5.075110 -0.035536
C -4.974559 4.080076 -0.033720
C -4.655149 2.727581 -0.025027
N 0.139006 3.349810 -0.014790
H 2.047171 5.594517 -0.015304
H 4.455176 6.296138 -0.005887
H 6.259451 4.610360 0.009425
H 5.721782 2.160002 0.015801
H 2.341746 -5.486593 0.011066
H 4.780397 -6.064358 0.028599
H 6.497995 -4.287702 0.038873
H 5.838672 -1.870011 0.031890
H 0.975085 -0.646881 -0.003266
H -5.313467 -2.237637 -0.061349
H -5.778804 -4.680323 -0.090876
H -3.941785 -6.333999 -0.079495
H -1.547307 -5.573643 -0.042907
H -1.845207 5.492388 -0.030895
H -4.274262 6.125787 -0.042716
H -6.024340 4.378142 -0.039837
H -5.432679 1.961472 -0.021202
H -0.554216 0.649935 -0.011138
H -5.105551 -0.141112 0.057576
O -6.070792 -0.141000 0.223950
H -6.110848 -0.185958 1.186935

N1b
E = -1743.823635
C -1.425625 5.339227 -0.123485
C -0.697182 4.152559 -0.094673
C 0.700442 4.154844 -0.127817
C 1.423076 5.343756 -0.190910
C 0.698518 6.533024 -0.217070
C -0.706930 6.530790 -0.183869
C -1.089324 2.744050 -0.035680
N 0.005651 1.933939 -0.025967
C 1.097271 2.747538 -0.087513
N 2.382279 2.388737 -0.107055
C 2.779691 1.138282 -0.110492
C 4.164887 0.699939 -0.074495
C 4.164096 -0.704553 -0.074501
C 2.778411 -1.141345 -0.110481
N 2.014867 -0.001104 -0.139581
C 5.358898 1.422472 -0.035960
C 6.544838 0.700938 -0.001179
C 6.544040 -0.708258 -0.001186
C 5.357282 -1.428444 -0.035968
N 2.379616 -2.391368 -0.106981
C 1.094223 -2.748760 -0.087242
C 0.695874 -4.155636 -0.127776
C -0.701736 -4.151842 -0.094291
C -1.092349 -2.742913 -0.035009
N 0.003496 -1.933994 -0.025281
C 1.417190 -5.345317 -0.191354
C 0.691321 -6.533788 -0.217622
C -0.714110 -6.530026 -0.184072
C -1.431490 -5.337691 -0.123225
N -2.378811 -2.385769 -0.015210
C -2.779642 -1.137185 -0.008386
N -2.017566 0.001040 0.001402
C -2.778381 1.140139 -0.008840
C -4.166543 0.704456 -0.039217
C -4.167326 -0.699981 -0.038905
C -5.358991 1.428182 -0.073171
C -6.546608 0.707751 -0.102149
C -6.547392 -0.700667 -0.101833
C -5.360575 -1.422401 -0.072532
N -2.376170 2.388264 -0.015997
H -2.515600 5.325067 -0.098153
H -1.240986 7.482449 -0.205989
H 1.227927 7.486376 -0.264028
H 2.513086 5.333372 -0.217173
H 5.342431 -2.518397 -0.033173
H 7.496585 -1.239924 0.028274
H 7.497985 1.231525 0.028292
H 5.345292 2.512441 -0.033153
H 1.001666 -0.000462 -0.197427
H -2.521442 -5.322336 -0.097649
H -1.249213 -7.481095 -0.206297
H 1.219677 -7.487706 -0.264950
H 2.507205 -5.336130 -0.217888
H -5.344313 2.518148 -0.074418
H -7.499212 1.239616 -0.125634
H -7.500587 -1.231482 -0.125086
H -5.347100 -2.512383 -0.073297
H -1.011493 0.000474 0.119354
H 0.152835 0.757637 1.852153
O 0.000272 0.000540 2.433046
H 0.154430 -0.754551 1.849982

N1c
E = -1743.821383
C -2.379567 4.977759 -0.132890
C -1.440551 3.946440 -0.053684
C -0.062364 4.213464 -0.106243
C 0.417568 5.518824 -0.238031
C -0.517076 6.542603 -0.306755
C -1.900424 6.274744 -0.254984
C -1.604900 2.507367 0.062732
N -0.338240 1.974910 0.084508
C 0.629335 2.940159 -0.019921
N -2.765301 1.890732 0.082251
C -2.900843 0.565811 0.053165
C -4.214763 -0.072762 -0.019102
C -3.963230 -1.448470 -0.086213
C -2.507805 -1.583971 -0.049666
N -1.909310 -0.368993 0.044121
C -5.515221 0.427284 -0.039767
C -6.555903 -0.493743 -0.124047
C -6.303071 -1.875610 -0.190770
C -5.002564 -2.372348 -0.175385
N -1.908107 -2.777935 -0.101010
C -0.605620 -2.922760 -0.124123

C 0.082677 -4.205782 -0.129963
C 1.461196 -3.946142 -0.139148
C 1.633211 -2.500676 -0.143325
N 0.373804 -1.959323 -0.143849
C -0.407338 -5.512038 -0.119865
C 0.521293 -6.545554 -0.122268
C 1.905272 -6.284804 -0.131454
C 2.393505 -4.984044 -0.138994
N 2.791139 -1.886290 -0.137451
C 2.911178 -0.555259 -0.112560
C 4.232819 0.082233 -0.080424
C 3.982314 1.456764 -0.056584
C 2.520599 1.587523 -0.070511
N 1.921230 0.367498 -0.111190
C 5.529991 -0.419452 -0.072152
C 6.574396 0.504130 -0.039629
C 6.322845 1.885290 -0.015631
C 5.019898 2.382510 -0.022896
N 1.934883 2.783951 -0.054275
O -0.135199 -0.131467 2.380784
H 4.812953 3.452979 -0.003437
H 7.164932 2.579400 0.009452
H 7.607173 0.150844 -0.031961
H 5.713049 -1.494263 -0.089621
H -1.481113 -5.699564 -0.110389
H 0.175791 -7.580742 -0.115929
H 2.603956 -7.123136 -0.131507
H 3.461744 -4.767105 -0.143797
H 0.202296 -0.957419 -0.154563
H -5.700224 1.500848 0.008652
H -7.588767 -0.140962 -0.140354
H -7.144582 -2.567652 -0.256860
H -4.794577 -3.441391 -0.229854
H 1.489717 5.710834 -0.282747
H -0.179552 7.575758 -0.405011
H -2.604695 7.106322 -0.314856
H -3.446488 4.756711 -0.098025
H -0.111392 1.005369 0.293944
H -0.450500 0.610259 2.908835
H -0.889974 -0.334151 1.806530

N2a
E = -1820.224239
C 5.402162 -1.086812 0.048694
C 4.185202 -0.395842 0.037268
C 4.161807 1.009408 0.053449
C 5.334548 1.764016 0.081335
C 6.539805 1.075174 0.094090
C 6.568684 -0.331446 0.077968
C 2.793970 -0.845083 0.015461
N 2.019516 0.285139 0.018089
C 2.773429 1.431969 0.037760
N 2.381732 -2.095143 0.001158
C 1.090678 -2.464986 -0.000204
C 0.699723 -3.879457 0.000649
C -0.701278 -3.879147 -0.000718
C -1.091622 -2.464513 0.000174
N -0.000302 -1.653327 -0.000027
C 1.423090 -5.072661 0.005151
C 0.699610 -6.263179 0.003532
C -0.702270 -6.262863 -0.003665
C -1.425197 -5.072011 -0.005251
N -2.382531 -2.094142 -0.001170
C -2.794266 -0.843922 -0.015485
C -4.185324 -0.394150 -0.037346
C -4.161410 1.011091 -0.053500
C -2.772867 1.433125 -0.037786
N -2.019387 0.286007 -0.018112
C -5.402526 -1.084679 -0.048838
C -6.568770 -0.328885 -0.078135
C -6.539377 1.077724 -0.094216
C -5.333870 1.766130 -0.081400
N -2.373970 2.681000 -0.038917
C -1.087890 3.038525 -0.018862
C -0.697642 4.450353 -0.012646
C 0.699435 4.450060 0.012696
C 1.089093 3.038067 0.018880
N 0.000428 2.225991 -0.000001
C -1.423283 5.638068 -0.025741
C -0.701000 6.830036 -0.012701
C 0.703796 6.829739 0.012808
C 1.425576 5.637467 0.025819
N 2.375020 2.680006 0.038914
O 4.473964 -4.099821 -0.317070
H 2.515745 5.625469 0.045613
H 1.236006 7.782649 0.022519
H -1.232808 7.783170 -0.022391
H -2.513458 5.626532 -0.045536
H -5.436433 -2.175395 -0.030304
H -7.532490 -0.840774 -0.089043
H -7.479201 1.631933 -0.117364
H -5.286163 2.855051 -0.093645
H -1.003894 0.272883 -0.004188
H 2.513818 -5.081845 0.007469
H 1.235988 -7.213757 0.007379
H -1.239080 -7.213196 -0.007532
H -2.515928 -5.080665 -0.007554
H 5.287237 2.852954 0.093602
H 7.479831 1.629041 0.117209
H 7.532216 -0.843689 0.088815
H 5.435676 -2.177539 0.030102
H 1.004017 0.272408 0.004160
H 3.763704 -3.447790 -0.144872
H 4.475606 -4.153827 -1.280428
H -3.765179 -3.446279 0.145158
O -4.475699 -4.097931 0.317674
H -4.476980 -4.151916 1.281035

N2b
E = -1820.222303
C 3.256957 4.740515 0.004722
C 2.913164 3.391888 -0.004279
C 3.887906 2.390799 0.012406
C 5.244733 2.699382 0.038934
C 5.594602 4.048623 0.047470
C 4.614552 5.055071 0.030741
C 1.628934 2.687168 -0.029928
N 1.806737 1.340656 -0.029355
C 3.149459 1.125268 -0.001286
N 3.791311 -0.045663 0.017834
C 3.166591 -1.199479 0.014814
C 3.810730 -2.503332 0.034479
C 2.796077 -3.474280 0.024004
C 1.521562 -2.773989 -0.004446
N 1.816635 -1.434371 -0.006714
C 5.158631 -2.864910 0.058921
C 5.458993 -4.220931 0.072955
C 4.441310 -5.195171 0.062302
C 3.099592 -4.836737 0.037286
N 0.344238 -3.350540 -0.027515
C -0.806736 -2.677381 -0.073266
C -2.086371 -3.383358 -0.106878
C -3.069355 -2.389041 -0.166070
C -2.330190 -1.120297 -0.148030
N -0.992508 -1.327592 -0.100373
C -2.413651 -4.736249 -0.100438
C -3.764514 -5.069889 -0.159986
C -4.752374 -4.074572 -0.235029
C -4.423764 -2.719722 -0.242157
N -2.971914 0.062319 -0.152496
C -2.352095 1.224270 -0.134992
C -2.995552 2.533377 -0.111875
C -1.970530 3.494220 -0.071777
C -0.699873 2.788508 -0.077102
N -1.002369 1.448847 -0.119909
C -2.252302 4.859548 -0.036552

C -3.588924 5.237927 -0.045224
C -4.615987 4.277482 -0.093598
C -4.340066 2.915385 -0.128198
N 0.476456 3.363144 -0.044167
O -6.094000 0.158125 -0.644720
O -5.101361 -0.256209 1.943321
H 2.487660 5.513159 -0.008088
H 4.925967 6.101167 0.038482
H 6.648349 4.332514 0.067667
H 5.996917 1.910060 0.052265
H 2.303049 -5.580844 0.028055
H 4.716945 -6.251020 0.073675
H 6.501706 -4.542231 0.092356
H 5.935892 -2.100663 0.066414
H -5.199697 -1.957916 -0.332971
H -5.801636 -4.368221 -0.298140
H -4.063325 -6.119664 -0.157858
H -1.633254 -5.496350 -0.053793
H -1.442992 5.589038 -0.004651
H -3.850390 6.297121 -0.018504
H -5.655257 4.609397 -0.107358
H -5.143231 2.178760 -0.189459
H -0.311161 0.704452 -0.127438
H -5.162757 0.129687 -0.906454
H -6.016762 0.050266 0.321774
H -5.061565 -1.206301 2.107490
H -4.302387 -0.091981 1.415017
H 1.121665 -0.693927 -0.015959

N2c
E = -1820.218083
C -0.112194 -5.478689 -0.546971
C -0.454593 -4.134268 -0.379649
C -1.793608 -3.712839 -0.435278
C -2.829061 -4.623698 -0.661856
C -2.487344 -5.959780 -0.816840
C -1.143133 -6.382821 -0.760135
C 0.362589 -2.956651 -0.150681
N -0.494578 -1.887808 -0.088949
C -1.801877 -2.273632 -0.246093
N 1.676104 -2.969167 -0.043542
C 2.410286 -1.861461 0.022350
C 3.872535 -1.911166 0.151512
C 4.291511 -0.576521 0.140438
C 3.062664 0.216571 0.003476
N 1.973208 -0.576652 -0.054454
C 4.784881 -2.955361 0.262488
C 6.135037 -2.620917 0.365019
C 6.555287 -1.281342 0.353844
C 5.636926 -0.237928 0.239426
N 3.100719 1.552529 -0.072904
C 2.021493 2.289731 -0.183470
C 2.030738 3.742588 -0.309756
C 0.696173 4.167007 -0.372735
C -0.144093 2.978570 -0.287517
N 0.706904 1.905501 -0.196898
C 3.084074 4.654546 -0.366589
C 2.761173 6.001318 -0.486849
C 1.421097 6.427600 -0.549908
C 0.371582 5.517635 -0.494682
N -1.453641 2.997702 -0.280170
C -2.196179 1.883375 -0.243210
C -3.657049 1.940063 -0.118299
C -4.080978 0.604651 -0.115964
C -2.854154 -0.191452 -0.236315
N -1.762553 0.607891 -0.311140
C -4.566812 2.987968 -0.003906
C -5.916250 2.659561 0.114530
C -6.341733 1.320423 0.118183
C -5.429055 0.273200 0.002491
N -2.887280 -1.525457 -0.242774
O -0.129534 0.104892 2.013832
H -5.750067 -0.769386 0.000710
H -7.406792 1.100219 0.211378
H -6.659019 3.454093 0.206592
H -4.225812 4.023846 -0.006617
H 4.116943 4.309447 -0.316799
H 3.558478 6.745083 -0.533647
H 1.206281 7.493501 -0.644011
H -0.670651 5.833573 -0.542865
H 0.394545 0.948418 -0.038010
H 4.448226 -3.992659 0.267284
H 6.881499 -3.412394 0.454374
H 7.620388 -1.056587 0.435204
H 5.951841 0.806072 0.227014
H -3.863589 -4.283244 -0.712237
H -3.270191 -6.700402 -0.989406
H -0.914052 -7.441966 -0.889941
H 0.931593 -5.790812 -0.507644
H -0.220116 -0.968496 0.271964
H -1.031729 -0.057611 2.354360
H 0.447178 -0.387068 2.608330
O -2.784612 -0.452447 2.792097
H -3.316522 0.303866 2.509689
H -3.067071 -1.146517 2.180361

N3a
E = -1896.618879
C -3.591976 -3.520576 -0.210052
C -2.318887 -2.941565 -0.158917
C -1.168649 -3.748894 -0.120916
C -1.247492 -5.141678 -0.132813
C -2.511748 -5.712153 -0.184288
C -3.666125 -4.908318 -0.222865
C -0.010119 -2.875563 -0.073719
N -0.496075 -1.591577 -0.083883
C -1.865005 -1.552644 -0.133521
N -2.629223 -0.478278 -0.154130
C -2.141359 0.773726 -0.130993
C -3.022057 1.951197 -0.149202
C -2.161124 3.052995 -0.130945
C -0.808273 2.501323 -0.095338
N -0.833472 1.140246 -0.095955
C -4.403137 2.130025 -0.179512
C -4.889321 3.435327 -0.198384
C -4.021151 4.537675 -0.182244
C -2.639571 4.359545 -0.146223
N 0.251067 3.312121 -0.067225
C 1.492712 2.893832 -0.029063
C 2.662394 3.758288 0.006178
C 3.797422 2.932255 0.044594
C 3.332845 1.554501 0.032762
N 1.963230 1.607069 -0.011763
C 2.780286 5.149253 0.007334
C 4.061074 5.684955 0.047534
C 5.199783 4.856020 0.086050
C 5.084109 3.471950 0.085227
N 4.101572 0.492287 0.060722
C 3.609607 -0.749986 0.047589
C 4.496090 -1.915309 0.079478
C 3.650588 -3.027839 0.052193
C 2.290712 -2.484884 0.005276
N 2.304140 -1.127145 0.004478
C 5.880217 -2.056653 0.126648
C 6.391802 -3.352721 0.145875
C 5.541767 -4.471371 0.118465
C 4.156840 -4.324522 0.071086
N 1.230541 -3.294682 -0.031963
O -5.512232 -1.002144 -0.187833
O -6.823616 0.017740 2.009173
O -7.479030 0.833677 -0.527018
H 3.487757 -5.185284 0.049436
H 5.978133 -5.471769 0.134756
H 7.471911 -3.506015 0.182894
H 6.530235 -1.181379 0.147523
H 1.891013 5.779028 -0.022364

H 4.192514 6.768329 0.049652
H 6.189673 5.314405 0.117241
H 5.956596 2.819114 0.114980
H -5.093568 1.292479 -0.184970
H -5.969023 3.589783 -0.227148
H -4.436280 5.547163 -0.196753
H -1.948866 5.203191 -0.130902
H -0.338866 -5.743118 -0.102750
H -2.615741 -6.798510 -0.195694
H -4.645835 -5.387521 -0.263862
H -4.493046 -2.906169 -0.240518
H 0.074247 -0.750915 -0.058891
H -4.561095 -0.772583 -0.239661
H -5.757818 -0.805353 0.741497
H 1.380941 0.774856 -0.030425
H -7.282369 0.459796 1.265070
H -7.484853 -0.587433 2.362831
H -6.810358 0.136969 -0.711032
H -8.212280 0.647307 -1.121215

N3b
E = -1896.618688
C 4.884795 -2.913211 -0.013962
C 3.528033 -2.571704 -0.018066
C 2.539041 -3.569352 -0.049035
C 2.864121 -4.925238 -0.076944
C 4.211382 -5.261104 -0.074326
C 5.205105 -4.265695 -0.043227
C 2.833426 -1.285716 -0.001529
N 1.492383 -1.563854 -0.019470
C 1.243745 -2.913096 -0.046333
N 3.396361 -0.096978 0.020135
C 2.705716 1.055365 0.012023
C 3.386286 2.354231 0.017959
C 2.363264 3.311200 0.000613
C 1.111610 2.542992 -0.015298
N 1.355002 1.205523 -0.006383
C 4.730304 2.727514 0.034780
C 5.017868 4.090557 0.038725
C 3.995105 5.049003 0.027300
C 2.652705 4.676295 0.007776
N -0.084388 3.151759 -0.029646
C -1.244753 2.531669 -0.012579
C -2.553087 3.178532 0.009697
C -3.524936 2.160977 0.038524
C -2.819627 0.885896 0.025659
N -1.480119 1.179961 -0.000794
C -2.922724 4.527366 0.012537
C -4.280251 4.821246 0.044368
C -5.249617 3.804657 0.072575
C -4.889815 2.462910 0.070143
N -3.383699 -0.303276 0.028237
C -2.694545 -1.454595 -0.003450
C -3.365297 -2.763900 -0.033538
C -2.326482 -3.700289 -0.064246
C -1.088950 -2.924307 -0.049136
N -1.343855 -1.589710 -0.015906
C -4.698533 -3.176631 -0.044254
C -4.944432 -4.548597 -0.085188
C -3.898466 -5.484022 -0.114007
C -2.568884 -5.069690 -0.103792
N 0.095171 -3.539589 -0.063185
O 6.290308 -0.082522 0.359618
H -1.741829 -5.779813 -0.127563
H -4.133920 -6.549316 -0.146238
H -5.976631 -4.903165 -0.096133
H -5.521759 -2.460938 -0.020010
H -2.174784 5.321013 -0.019710
H -4.598939 5.864969 0.046729
H -6.307040 4.073260 0.096558
H -5.644143 1.674712 0.095564
H -0.756225 0.467254 -0.014679
H 5.530939 1.986728 0.046125
H 6.059291 4.416937 0.051250
H 4.253798 6.109355 0.032942
H 1.864925 5.430304 -0.008374
H 2.077355 -5.679254 -0.101049
H 4.507443 -6.311222 -0.097072
H 6.255191 -4.562583 -0.042285
H 5.662459 -2.147886 0.015402
H 0.778085 -0.841476 -0.012276
H 5.328344 -0.066602 0.177566
H 6.318615 -0.046965 1.323424
H -0.138696 5.083054 -0.280249
O -0.191601 6.044351 -0.458657
H -0.154205 6.083268 -1.421952
H -5.314080 -0.385401 0.190053
O -6.275530 -0.404787 0.374192
H -6.300254 -0.459014 1.337223

N3c
E = -1896.618486
C 3.536201 -3.584241 -0.143552
C 2.270545 -2.988420 -0.120271
C 1.108206 -3.778912 -0.117710
C 1.168051 -5.172459 -0.142154
C 2.425351 -5.760004 -0.169365
C 3.591772 -4.972654 -0.167747
C -0.037873 -2.888341 -0.085606
N 0.467444 -1.611580 -0.079130
C 1.837337 -1.593364 -0.101323
N 2.619199 -0.531884 -0.121604
C 2.150581 0.727500 -0.130956
C 3.048993 1.888489 -0.211450
C 2.206734 3.004931 -0.190505
C 0.846819 2.476952 -0.102444
N 0.850009 1.115755 -0.075936
C 4.429027 2.042674 -0.320779
C 4.933184 3.339248 -0.397106
C 4.084828 4.456349 -0.366413
C 2.703758 4.302414 -0.263474
N -0.198435 3.305134 -0.059388
C -1.445976 2.906585 -0.002653
C -2.601507 3.789048 0.049701
C -3.749044 2.980555 0.091758
C -3.306517 1.595801 0.064108
N -1.936534 1.627286 0.012048
C -2.697592 5.181665 0.060928
C -3.969446 5.737025 0.115596
C -5.120638 4.925696 0.157777
C -5.026735 3.540031 0.146138
N -4.092209 0.545867 0.080988
C -3.621276 -0.704134 0.047263
C -4.527599 -1.854500 0.057031
C -3.701146 -2.980718 0.011608
C -2.332145 -2.460495 -0.021554
N -2.322295 -1.102852 0.000062
C -5.914070 -1.973115 0.099006
C -6.447623 -3.260399 0.094044
C -5.616694 -4.392762 0.048414
C -4.229390 -4.268732 0.006621
N -1.285396 -3.287724 -0.062929
O 5.440552 -1.117186 0.413414
O 7.293964 0.571611 -0.776127
O 7.114238 0.400041 1.933513
H -3.574931 -5.140226 -0.028720
H -6.070080 -5.385693 0.045915
H -7.530268 -3.395874 0.126168
H -6.549177 -1.087407 0.134292
H -1.798844 5.797674 0.027712
H -4.083833 6.822279 0.126212
H -6.102948 5.399244 0.200288
H -5.909122 2.900727 0.177929
H 5.113943 1.200942 -0.345483
H 6.011938 3.475811 -0.486280
H 4.514833 5.457785 -0.427815

H 2.027172 5.157333 -0.244464
H 0.250953 -5.761660 -0.139622
H 2.514420 -6.847563 -0.189283
H 4.565382 -5.465564 -0.183145
H 4.444671 -2.981216 -0.119959
H -0.091677 -0.763373 -0.056684
H 4.518505 -0.850013 0.216813
H 5.981531 -0.719697 -0.296277
H -1.366829 0.786767 -0.015774
H 7.470349 0.651203 0.188579
H 8.132096 0.298231 -1.161158
H 6.366592 -0.167627 1.641141
H 7.686125 -0.208086 2.414864

N3d
E = -1896.617955
C 4.080345 -2.905700 -0.298132
C 2.736580 -2.536185 -0.182056
C 1.715756 -3.499123 -0.105218
C 2.007657 -4.863379 -0.105988
C 3.344296 -5.232698 -0.193068
C 4.363603 -4.266944 -0.296551
C 0.446069 -2.793032 -0.055016
N 0.748323 -1.452153 -0.090642
C 2.094850 -1.229455 -0.146783
N 2.723223 -0.071103 -0.130844
C 2.093372 1.114335 -0.096107
C 2.846080 2.371293 -0.059640
C 1.875288 3.378259 -0.052137
C 0.586370 2.685954 -0.074439
N 0.757570 1.334273 -0.098396
C 4.205452 2.676799 -0.033228
C 4.555875 4.025446 -0.004918
C 3.580974 5.036243 -0.000874
C 2.223110 4.725458 -0.022905
N -0.560280 3.368138 -0.067687
C -1.740337 2.795839 -0.057910
C -3.012843 3.500615 -0.048725
C -4.030498 2.533197 -0.024079
C -3.390637 1.226420 -0.016985
N -2.039745 1.457341 -0.042603
C -3.311760 4.863944 -0.057903
C -4.652517 5.227054 -0.042671
C -5.673147 4.256451 -0.017965
C -5.377041 2.899262 -0.007802
N -4.019964 0.076533 0.015893
C -3.386156 -1.100025 0.027930
C -4.135251 -2.358315 0.077431
C -3.169387 -3.368354 0.077951
C -1.879546 -2.676333 0.026133
N -2.046722 -1.327093 -0.000591
C -5.494634 -2.654175 0.118600
C -5.856385 -3.999547 0.160764
C -4.885334 -5.014916 0.161261
C -3.525296 -4.713337 0.119437
N -0.732798 -3.361233 0.004751
O 5.567567 -0.399703 -1.516326
O 6.986634 0.685650 0.624429
H -2.763077 -5.493083 0.118629
H -5.205923 -6.057730 0.194680
H -6.912461 -4.273382 0.193783
H -6.239726 -1.858038 0.117349
H -2.512728 5.605193 -0.076560
H -4.924710 6.283821 -0.049749
H -6.714906 4.581205 -0.006488
H -6.156734 2.137721 0.011925
H 4.973343 1.902492 -0.029121
H 5.610984 4.304928 0.011031
H 3.896189 6.080956 0.020182
H 1.454827 5.499136 -0.018094
H 1.206385 -5.599933 -0.045461
H 3.611390 -6.290864 -0.197469
H 5.399690 -4.595144 -0.394519
H 4.857748 -2.150081 -0.447207
H 0.054316 -0.711266 -0.056495
H 4.691704 -0.072321 -1.271021
H 6.168784 0.069124 -0.909197
H 6.301140 0.297331 1.211465
H 7.152135 1.561095 0.987985
H -1.343950 0.717906 -0.048778
H 4.172715 -0.262254 1.174667
O 4.836751 -0.422794 1.872618
H 4.878558 -1.385262 1.923090

N3e
E = -1896.617410
C 2.835451 5.196118 -0.010755
C 2.595562 3.825344 -0.013697
C 3.643409 2.901366 0.010594
C 4.972757 3.311785 0.039079
C 5.218943 4.683872 0.041491
C 4.165423 5.612769 0.017002
C 1.369555 3.023905 -0.038153
N 1.648570 1.694696 -0.030623
C 3.002488 1.583760 0.001652
N 3.731373 0.466270 0.028425
C 3.210053 -0.736269 0.029041
C 3.978397 -1.967501 0.058728
C 3.072181 -3.042082 0.047642
C 1.729570 -2.466285 0.009670
N 1.888940 -1.104797 0.002795
C 5.357904 -2.169691 0.095992
C 5.812764 -3.481065 0.123817
C 4.909712 -4.560040 0.113693
C 3.534302 -4.362688 0.074649
N 0.596376 -3.136244 -0.013021
C -0.615805 -2.561921 -0.054087
C -1.847750 -3.359864 -0.070496
C -2.892091 -2.427660 -0.132672
C -2.244107 -1.113816 -0.130267
N -0.893570 -1.229392 -0.089699
C -2.107055 -4.730920 -0.040872
C -3.440044 -5.134230 -0.083618
C -4.483361 -4.200511 -0.165902
C -4.226580 -2.831565 -0.193860
N -2.974621 0.015849 -0.140415
C -2.459408 1.226814 -0.132662
C -3.217109 2.474842 -0.117915
C -2.281470 3.522971 -0.084786
C -0.954222 2.932281 -0.086298
N -1.135097 1.570882 -0.120515
C -2.680222 4.858702 -0.058044
C -4.044662 5.119352 -0.068273
C -4.983093 4.072314 -0.110313
C -4.589542 2.739080 -0.136413
N 0.167413 3.607128 -0.056973
O -6.134252 -0.121699 -0.656851
O 0.834500 -6.030896 -0.242286
H 2.009900 5.908196 -0.029334
H 4.396439 6.679486 0.020151
H 6.247998 5.047045 0.063061
H 5.782842 2.582183 0.058650
H 2.842095 -5.206471 0.063984
H 5.298944 -5.579236 0.137656
H 6.884967 -3.681752 0.154849
H 6.038396 -1.318229 0.103978
H 1.113062 -0.449635 -0.013397
H -5.042853 -2.114333 -0.287620
H -5.515517 -4.551648 -0.216079
H -3.675787 -6.199625 -0.061076
H -1.301777 -5.465282 0.009614
H -1.937080 5.655651 -0.031270
H -4.397699 6.151749 -0.048027
H -6.047502 4.311406 -0.125988
H -5.327618 1.937511 -0.194278
H -0.371876 0.900803 -0.122363

H -5.208769 -0.085854 -0.935733
H -6.035005 -0.204316 0.310480
H 0.737879 -5.067655 -0.095443
H 0.813926 -6.095806 -1.204783
O -5.105105 -0.401009 1.929550
H -5.012664 -1.339580 2.133854
H -4.310834 -0.211522 1.402178

N3f
E = -1896.616991
C -1.539354 -5.161938 -0.719960
C -1.472887 -3.782780 -0.510047
C -2.620492 -2.977787 -0.602971
C -3.867865 -3.528688 -0.909979
C -3.934125 -4.902428 -1.100718
C -2.782254 -5.709805 -1.007549
C -0.355216 -2.907531 -0.200892
N -0.854044 -1.632290 -0.127126
C -2.200995 -1.609876 -0.355966
N 0.888718 -3.310433 -0.053826
C 1.916009 -2.469160 0.031994
C 3.290426 -2.955204 0.208848
C 4.095470 -1.812209 0.185713
C 3.167768 -0.690293 -0.005606
N 1.890154 -1.114266 -0.083297
C 3.837773 -4.223520 0.369726
C 5.222801 -4.312143 0.508947
C 6.030899 -3.164451 0.485411
C 5.477025 -1.894780 0.321662
N 3.606043 0.571612 -0.091859
C 2.799005 1.594078 -0.235838
C 3.240390 2.980143 -0.291832
C 2.097713 3.784999 -0.398942
C 0.939622 2.903798 -0.410290
N 1.426370 1.616374 -0.326571
C 4.518218 3.538844 -0.240877
C 4.615983 4.922971 -0.306069
C 3.467736 5.732072 -0.413547
C 2.195539 5.176082 -0.458563
N -0.302344 3.318717 -0.438818
C -1.346459 2.481798 -0.403160
C -2.723437 2.972169 -0.299439
C -3.524517 1.823135 -0.259152
C -2.586090 0.697701 -0.349145
N -1.307234 1.132843 -0.429577
C -3.276701 4.247183 -0.221308
C -4.661739 4.340877 -0.098109
C -5.465367 3.190096 -0.048262
C -4.909230 1.914234 -0.127799
N -3.010200 -0.568639 -0.370454
O -2.796784 0.775177 2.738303
H -5.533225 1.022125 -0.066623
H -6.546105 3.298707 0.058018
H -5.133142 5.323131 -0.032581
H -2.641026 5.132698 -0.252686
H 5.396966 2.899721 -0.153697
H 5.598552 5.396660 -0.272270
H 3.584295 6.816153 -0.460775
H 1.298410 5.790222 -0.538037
H 0.842900 0.778988 -0.367748
H 3.200959 -5.108610 0.385182
H 5.690457 -5.289975 0.638116
H 7.111411 -3.271433 0.597390
H 6.095295 -0.996738 0.300506
H -4.752075 -2.895390 -0.994097
H -4.892901 -5.368987 -1.333283
H -2.873720 -6.785281 -1.168632
H -0.639684 -5.773897 -0.652865
H -0.380499 -0.877684 0.398178
H -3.541472 0.182208 2.503080
H -3.048181 1.609712 2.324231
O -4.847524 -0.862997 1.736656
H -4.398874 -0.868612 0.865926

H -4.772348 -1.775372 2.040417
H 0.283915 0.714600 2.033010
O -0.237374 -0.093911 2.080171
H -1.132569 0.217955 2.341210

N3g
E = -1896.614176
C -2.384440 -4.768549 -0.745504
C -2.105427 -3.403416 -0.666582
C -3.133616 -2.450386 -0.673596
C -4.473797 -2.832215 -0.763774
C -4.750949 -4.190689 -0.843875
C -3.717247 -5.148551 -0.834819
C -0.847762 -2.687270 -0.546691
N -1.157168 -1.349392 -0.530405
C -2.511999 -1.140908 -0.568996
N 0.322928 -3.272536 -0.438902
C 1.453027 -2.592246 -0.248195
C 2.744787 -3.270672 -0.081091
C 3.681422 -2.245427 0.084923
C 2.910602 -0.996219 0.009722
N 1.595028 -1.247344 -0.181477
C 3.125290 -4.608329 -0.070525
C 4.478879 -4.890027 0.114764
C 5.419022 -3.860853 0.281605
C 5.032265 -2.520637 0.268038
N 3.512396 0.193925 0.064707
C 2.853443 1.329868 0.014151
C 3.483347 2.626631 -0.192623
C 2.460502 3.584397 -0.237717
C 1.199207 2.878660 -0.060264
N 1.503769 1.547463 0.093326
C 4.820813 2.986181 -0.362407
C 5.103045 4.331889 -0.562328
C 4.075543 5.294342 -0.607653
C 2.742411 4.933756 -0.454459
N 0.022020 3.457046 -0.085026
C -1.123310 2.772216 -0.151862
C -2.426944 3.438652 -0.080060
C -3.376585 2.416762 -0.211913
C -2.596959 1.185676 -0.366850
N -1.271621 1.436606 -0.325302
C -2.811965 4.766215 0.088006
C -4.177444 5.042753 0.119159
C -5.130256 4.018511 -0.015498
C -4.742421 2.690690 -0.185342
N -3.186208 -0.012166 -0.486572
O -1.184835 -2.300560 2.355366
H -5.475774 1.890953 -0.299675
H -6.191805 4.272003 0.009478
H -4.517188 6.072205 0.247309
H -2.064159 5.553580 0.189319
H 5.604201 2.228574 -0.334269
H 6.138215 4.653120 -0.690361
H 4.335706 6.341871 -0.769757
H 1.936671 5.666875 -0.497122
H 0.848387 0.864066 0.509845
H 2.386633 -5.399594 -0.203377
H 4.817043 -5.927892 0.129962
H 6.470558 -4.117734 0.422873
H 5.753939 -1.712863 0.394533
H -5.264385 -2.081294 -0.772591
H -5.786720 -4.526918 -0.917246
H -3.973707 -6.207412 -0.900391
H -1.574141 -5.497758 -0.735576
H -0.465498 -0.609248 -0.403209
H -1.941272 -1.728179 2.575225
H -1.121593 -2.926649 3.083271
O -2.974309 -0.167702 2.456879
H -3.433556 -0.096316 1.603869
H -3.375819 0.506638 3.014768
H -0.917672 0.441419 2.044028
O 0.015154 0.181486 2.026852

H -0.059055 -0.785294 2.127405

N4a
E = -1973.024748
C 2.764325 4.952241 -0.425076
C 2.509692 3.585028 -0.292768
C 3.559165 2.655833 -0.202466
C 4.893992 3.066029 -0.241534
C 5.144695 4.425837 -0.363427
C 4.091285 5.358675 -0.454027
C 1.264536 2.839413 -0.241035
N 1.604550 1.515182 -0.121835
C 2.962507 1.336191 -0.092675
N 0.066486 3.375105 -0.312039
C -1.049406 2.655739 -0.410956
C -2.370917 3.288259 -0.379041
C -3.290395 2.238176 -0.496556
C -2.477069 1.024023 -0.599785
N -1.161415 1.306647 -0.552064
C -2.793896 4.607899 -0.234988
C -4.167206 4.844567 -0.215651
C -5.089262 3.790963 -0.335209
C -4.663185 2.471783 -0.477761
N -3.048206 -0.186897 -0.699333
C -2.358037 -1.300772 -0.720465
C -2.950169 -2.630802 -0.755531
C -1.902550 -3.555896 -0.666125
C -0.660659 -2.800895 -0.568950
N -1.000871 -1.474363 -0.659120
C -4.278232 -3.046021 -0.833721
C -4.524252 -4.414577 -0.824263
C -3.471575 -5.344025 -0.736142
C -2.147384 -4.927529 -0.653188
N 0.519172 -3.340827 -0.399971
C 1.634813 -2.616930 -0.241188
C 2.936515 -3.264224 -0.043737
C 3.856349 -2.218175 0.078871
C 3.067142 -0.987650 -0.046160
N 1.749121 -1.271129 -0.232820
C 3.337467 -4.594863 0.024914
C 4.694348 -4.846980 0.223401
C 5.618309 -3.796388 0.346802
C 5.211561 -2.464589 0.275708
N 3.641946 0.212254 -0.003282
O -2.191662 1.716178 2.604662
H 5.920829 -1.641397 0.368331
H 6.673443 -4.030749 0.499756
H 5.048815 -5.877658 0.283510
H 2.611160 -5.402276 -0.074325
H -5.086402 -2.317028 -0.897460
H -5.551470 -4.777982 -0.884734
H -3.703073 -6.410533 -0.731050
H -1.322096 -5.635874 -0.578623
H -0.333510 -0.709240 -0.581685
H -2.069520 5.417611 -0.140227
H -4.537235 5.865381 -0.104410
H -6.157585 4.013095 -0.313243
H -5.373282 1.648961 -0.568898
H 5.698716 2.333441 -0.176656
H 6.175195 4.784077 -0.392500
H 4.328455 6.419569 -0.550975
H 1.940789 5.662585 -0.500407
H 0.934149 0.779490 0.104774
H -2.910418 1.045802 2.485063
H -2.462445 2.456746 2.048583
O -3.906100 -0.335270 2.151136
H -3.214132 -1.026418 2.242024
H -4.060963 -0.321796 1.194865
H 0.559790 0.388124 2.753906
O -0.050096 0.232501 2.023662
H -0.790960 0.877263 2.183082
H -0.986755 -1.251740 2.151546
O -1.680562 -1.949640 2.189621

H -1.414654 -2.533448 2.906721

N4b
E = -1973.023087
C 5.194633 -2.867746 0.249153
C 3.855957 -2.507832 0.092222
C 2.846461 -3.479103 0.002739
C 3.144611 -4.840530 0.067360
C 4.478021 -5.198207 0.224558
C 5.490728 -4.223760 0.314384
C 3.217840 -1.203463 -0.011682
N 1.873738 -1.440908 -0.149943
C 1.579367 -2.780668 -0.157832
N 3.840939 -0.052355 0.018619
C 3.208580 1.122791 -0.081329
C 3.955399 2.382051 -0.030429
C 2.995469 3.390953 -0.144559
C 1.710578 2.697914 -0.260825
N 1.877902 1.345515 -0.225572
C 5.308647 2.679047 0.103763
C 5.670575 4.024764 0.117736
C 4.705327 5.039328 0.002897
C 3.351471 4.736529 -0.129285
N 0.569370 3.378732 -0.364189
C -0.605891 2.803549 -0.465811
C -1.880311 3.497638 -0.506788
C -2.891316 2.523634 -0.588920
C -2.239375 1.225442 -0.607132
N -0.894648 1.463641 -0.534070
C -2.188932 4.859220 -0.448693
C -3.530309 5.214610 -0.472901
C -4.544348 4.237642 -0.541413
C -4.242310 2.884183 -0.596977
N -2.855222 0.066544 -0.690030
C -2.230488 -1.110624 -0.640253
C -2.965298 -2.369257 -0.753172
C -2.002008 -3.373784 -0.631171
C -0.727492 -2.680930 -0.449725
N -0.897820 -1.330648 -0.462871
C -4.312206 -2.674730 -0.927869
C -4.661190 -4.021895 -0.978091
C -3.691854 -5.031874 -0.857991
C -2.345686 -4.721499 -0.681801
N 0.412276 -3.360230 -0.295091
O -3.504370 -1.860954 2.356805
H -1.584279 -5.495544 -0.582672
H -4.004037 -6.076843 -0.900810
H -5.707887 -4.301388 -1.109550
H -5.065540 -1.890824 -1.009898
H -1.394408 5.602498 -0.381700
H -3.810138 6.268383 -0.427989
H -5.588456 4.554261 -0.540475
H -5.024493 2.125341 -0.608196
H -0.183873 0.738873 -0.533080
H 6.048670 1.883338 0.194123
H 6.721846 4.299693 0.220498
H 5.025654 6.082652 0.018865
H 2.593886 5.515793 -0.217347
H 2.351039 -5.584430 -0.003618
H 4.749913 -6.253581 0.280067
H 6.526621 -4.543947 0.437773
H 5.968599 -2.103205 0.317230
H 1.183401 -0.703010 -0.245980
H -4.165640 -1.245671 1.957411
H -3.855534 -2.040996 3.235900
O -5.026639 0.101519 1.200797
H -4.507950 0.052899 0.378829
H -4.520975 0.781667 1.703112
H -0.870201 -0.445643 1.542609
O -1.342506 -0.209544 2.351970
H -2.047121 -0.898185 2.404739
H -2.443524 1.141333 2.428971
O -3.232347 1.731998 2.475009

H -2.962321 2.539998 2.025568

N4c
E = -1973.014889
C -3.143718 5.063066 0.137560
C -2.969590 3.682661 0.102062
C -4.052280 2.808750 0.231090
C -5.350720 3.280437 0.400269
C -5.530402 4.662068 0.436946
C -4.441972 5.540864 0.307377
C -1.791569 2.825976 -0.056821
N -2.137702 1.506744 -0.024526
C -3.480775 1.462130 0.143817
N -4.263695 0.378153 0.227592
C -3.795931 -0.841730 0.151263
C -4.596880 -2.055374 0.241560
C -3.723040 -3.146500 0.119158
C -2.377195 -2.613477 -0.046296
N -2.497105 -1.247468 -0.020855
C -5.967800 -2.241976 0.416933
C -6.435915 -3.549807 0.465072
C -5.559282 -4.644528 0.342454
C -4.192880 -4.458797 0.168625
N -1.294649 -3.336404 -0.182135
C -0.071463 -2.810558 -0.315425
C 1.102004 -3.674749 -0.451319
C 2.197999 -2.812545 -0.555957
C 1.629954 -1.460770 -0.483985
N 0.277084 -1.499828 -0.341003
C 1.253955 -5.057268 -0.487819
C 2.546856 -5.555737 -0.634131
C 3.648586 -4.692005 -0.739622
C 3.492858 -3.307162 -0.703970
N 2.401358 -0.373349 -0.568658
C 1.936883 0.861847 -0.536334
C 2.727475 2.073849 -0.680453
C 1.842833 3.161328 -0.560733
C 0.510720 2.625861 -0.359638
N 0.640399 1.259967 -0.361432
C 2.293874 4.480674 -0.653011
C 3.648244 4.684476 -0.872787
C 4.535144 3.596837 -1.009639
C 4.090727 2.284184 -0.920889
N -0.577921 3.347590 -0.213628
O 5.167888 -0.542781 0.146051
O 5.920299 1.579710 1.730155
O 6.091924 -1.206368 2.605040
H -2.292198 5.736493 0.035621
H -4.620433 6.617164 0.340614
H -6.533146 5.072579 0.568407
H -6.187779 2.588707 0.499389
H -3.503141 -5.297477 0.073370
H -5.964203 -5.656933 0.385730
H -7.502576 -3.735813 0.600807
H -6.635264 -1.385360 0.511324
H 4.353337 -2.640625 -0.774616
H 4.648763 -5.114368 -0.849513
H 2.709994 -6.634406 -0.666124
H 0.388479 -5.715206 -0.404615
H 1.592220 5.309330 -0.556697
H 4.036704 5.701275 -0.950629
H 5.593068 3.791227 -1.195335
H 4.776832 1.444817 -1.030834
H -0.131389 0.612053 -0.232393
H 4.218827 -0.577724 -0.094765
H 5.266261 -1.009608 1.008060
H 5.481805 0.974883 1.094864
H 5.525591 2.441430 1.557924
H -1.718604 -0.603299 -0.122531
H 6.941555 -1.019631 2.152167
H 5.877434 -0.331871 2.961855
H 7.345673 -0.368297 0.140938
O 7.955752 -0.106361 0.846227

H 7.550697 0.727086 1.147553

N4d
E = -1973.013429
C 4.527847 -3.738062 0.156538
C 3.859673 -2.513241 0.103973
C 4.573548 -1.310338 0.132452
C 5.962459 -1.277293 0.210023
C 6.630258 -2.497941 0.261538
C 5.919496 -3.708039 0.235691
C 2.444655 -2.113387 0.026835
N 2.314270 -0.762269 0.013656
C 3.572535 -0.249152 0.072126
N 3.944425 1.033847 0.076283
C 3.091642 2.026581 0.031112
C 3.444157 3.437939 0.030358
C 2.247161 4.169619 -0.021652
C 1.152014 3.212499 -0.052033
N 1.723016 1.967684 -0.020570
C 4.682907 4.079587 0.071299
C 4.686462 5.468658 0.058318
C 3.485252 6.202769 0.006300
C 2.251793 5.565172 -0.034043
N -0.119672 3.522907 -0.096039
C -1.097985 2.614320 -0.114639
C -2.495602 3.036428 -0.151398
C -3.249143 1.858671 -0.154978
C -2.260283 0.769159 -0.129050
N -0.992699 1.257579 -0.102158
C -3.092138 4.293103 -0.178146
C -4.484324 4.342686 -0.210861
C -5.246080 3.164450 -0.212092
C -4.640889 1.910042 -0.181570
N -2.626830 -0.522876 -0.141299
C -1.775345 -1.528777 -0.119156
C -2.124951 -2.945090 -0.140511
C -0.922100 -3.675694 -0.109373
C 0.172567 -2.714032 -0.066341
N -0.404719 -1.469166 -0.074498
C -0.924166 -5.074302 -0.118097
C -2.155780 -5.714676 -0.157964
C -3.356709 -4.986252 -0.189601
C -3.361615 -3.597363 -0.180939
N 1.453354 -3.016681 -0.022351
O -5.485204 -1.264753 -0.147537
O -6.842349 -0.332250 2.052924
O -7.610969 0.391452 -0.480323
H 3.990476 -4.687158 0.133645
H 6.469278 -4.649955 0.279525
H 7.719799 -2.517918 0.324407
H 6.494270 -0.325621 0.230963
H 1.314861 6.120892 -0.073794
H 3.529088 7.293141 -0.002014
H 5.636056 6.005543 0.089193
H 5.605102 3.499786 0.111883
H -5.251647 1.013309 -0.174443
H -6.335585 3.216522 -0.238128
H -4.991186 5.309185 -0.234161
H -2.481821 5.196713 -0.173957
H 0.006969 -5.642726 -0.099327
H -2.189764 -6.805461 -0.164873
H -4.306909 -5.522254 -0.220867
H -4.297573 -3.038300 -0.204919
H 0.118413 -0.598587 -0.052042
H -4.554739 -0.966662 -0.209229
H -5.737335 -1.080654 0.783087
H 1.195890 1.099710 -0.033424
H -7.352782 0.058195 1.313700
H -7.444612 -0.979524 2.436115
H -6.889659 -0.248103 -0.670230
H -8.337734 0.135011 -1.056153
H 1.936103 -4.889654 -0.168619
O 2.158781 -5.828440 -0.338061

H 2.251030 -5.848945 -1.298243

N4e
E = -1973.012955
C 5.382413 1.416987 0.061963
C 4.184260 0.696848 0.030662
C 4.182217 -0.708887 -0.030472
C 5.378288 -1.432493 -0.061493
C 6.565437 -0.711170 -0.030211
C 6.567471 0.692227 0.030942
C 2.792383 1.133651 0.042361
N 2.025784 -0.002875 -0.000097
C 2.789068 -1.141639 -0.042417
N 2.381345 2.382274 0.072211
C 1.092559 2.756244 0.038229
C 0.705883 4.171893 0.026094
C -0.693820 4.173886 -0.026312
C -1.084537 2.759343 -0.038335
N 0.002857 1.945491 -0.000025
C 1.431278 5.363431 0.049104
C 0.709824 6.555216 0.023342
C -0.690943 6.557215 -0.023772
C -1.415803 5.367495 -0.049426
N -2.374395 2.389065 -0.072239
C -2.789054 1.141641 -0.042322
C -4.182203 0.708883 -0.030456
C -4.184244 -0.696852 0.030655
C -2.792366 -1.133650 0.042435
N -2.025769 0.002878 0.000037
C -5.378273 1.432486 -0.061558
C -6.565422 0.711158 -0.030383
C -6.567455 -0.692241 0.030731
C -5.382396 -1.416997 0.061827
N -2.381330 -2.382273 0.072273
C -1.092541 -2.756236 0.038251
C -0.705874 -4.171883 0.026087
C 0.693825 -4.173889 -0.026407
C 1.084550 -2.759345 -0.038419
N -0.002841 -1.945486 -0.000046
C -1.431295 -5.363401 0.049142
C -0.709867 -6.555201 0.023310
C 0.690896 -6.557220 -0.023919
C 1.415780 -5.367514 -0.049603
N 2.374406 -2.389065 -0.072340
O 4.442058 4.401937 0.554504
H 2.505541 -5.378584 -0.092980
H 1.225941 -7.508351 -0.041452
H -1.247659 -7.504783 0.040727
H -2.521082 -5.371275 0.092549
H -5.381075 2.522173 -0.117888
H -7.515387 1.247684 -0.054047
H -7.518968 -1.226014 0.054463
H -5.388333 -2.506670 0.118164
H -1.009952 0.001335 0.000036
H 2.521071 5.371353 0.092397
H 1.247602 7.504807 0.040722
H -1.226005 7.508337 -0.041248
H -2.505566 5.378534 -0.092730
H 5.381097 -2.522179 -0.117830
H 7.515401 -1.247700 -0.053811
H 7.518985 1.225994 0.054772
H 5.388345 2.506659 0.118341
H 1.009968 -0.001326 -0.000198
H 3.745451 3.743857 0.354891
H 4.424188 4.443279 1.518231
H -3.734634 3.754483 -0.354938
O -4.429332 4.414588 -0.554527
H -4.411804 4.455423 -1.518281
H -3.745564 -3.743803 0.355179
O -4.442091 -4.401957 0.554800
H -4.424568 -4.442919 1.518549
H 3.734643 -3.754517 -0.354799
O 4.429341 -4.414623 -0.554387
H 4.411906 -4.455342 -1.518149

N4f
E = -1973.010197
C -5.385547 0.843092 0.170691
C -4.179485 0.137638 0.135176
C -4.165270 -1.267350 0.102371
C -5.345412 -2.009730 0.092955
C -6.543587 -1.307058 0.120416
C -6.559992 0.098931 0.162228
C -2.776973 -1.696172 0.085490
N -2.017046 -0.552510 0.106629
C -2.788298 0.578412 0.127625
N -2.378642 1.829195 0.123551
C -1.085663 2.194186 0.074593
C -0.696609 3.606637 0.059981
C 0.699755 3.606066 -0.059590
C 1.087660 2.193301 -0.074360
N 0.000662 1.382607 0.000057
C -1.416775 4.799691 0.144115
C -0.694910 5.989577 0.076671
C 0.699990 5.989002 -0.076467
C 1.420883 4.798525 -0.143898
N 2.380346 1.827214 -0.123324
C 2.788863 0.576048 -0.127382
C 4.179651 0.134015 -0.134939
C 4.164147 -1.270959 -0.102166
C 2.775451 -1.698527 -0.085291
N 2.016579 -0.554169 -0.106343
C 5.386347 0.838384 -0.170485
C 6.560108 0.093150 -0.162075
C 6.542419 -1.312823 -0.120291
C 5.343609 -2.014415 -0.092806
N 2.374764 -2.945200 -0.055435
C 1.087370 -3.300595 -0.025245
C 0.696356 -4.712591 -0.014618
C -0.700458 -4.711989 0.014555
C -1.090274 -3.299670 0.025302
N -0.001112 -2.487403 0.000078
C 1.422025 -5.899675 -0.030079
C 0.699025 -7.091695 -0.014826
C -0.705209 -7.091081 0.014515
C -1.427164 -5.898434 0.029895
N -2.377378 -2.943198 0.055541
O -4.620320 4.015623 0.680317
O -3.732983 3.556422 -1.935011
O 4.623780 4.011218 -0.681094
H -2.517208 -5.886345 0.052803
H -1.237617 -8.043776 0.025389
H 1.230602 -8.044852 -0.025805
H 2.512079 -5.888534 -0.052991
H 5.412566 1.927791 -0.228382
H 7.518853 0.613021 -0.191805
H 7.487598 -1.858169 -0.114184
H 5.309171 -3.103525 -0.066020
H 1.000909 -0.547487 -0.101602
H -2.498955 4.811234 0.282504
H -1.226088 6.940570 0.143690
H 1.231927 6.939564 -0.143563
H 2.503024 4.809188 -0.282578
H -5.311965 -3.098872 0.066148
H -7.489259 -1.851549 0.114270
H -7.518260 0.619679 0.191927
H -5.410815 1.932522 0.228597
H -1.001369 -0.547003 0.101627
H -3.949284 3.365918 0.932192
H -4.520272 4.019508 -0.290288
H 3.952278 3.362034 -0.933040
H 4.523317 4.015623 0.289476
H -3.044429 4.196916 -2.151503
H -3.254498 2.882590 -1.424184
O 3.736203 3.554245 1.933789
H 3.047838 4.194290 2.152194

H 3.257025 2.880496 1.423486

C0a
E = -1667.213111
C -0.000000 4.154569 0.698058
C -0.000000 5.333386 1.426609
C -0.000000 6.530758 0.699239
C 0.000000 6.530758 -0.699239
C 0.000000 5.333386 -1.426609
C 0.000000 4.154569 -0.698058
H -0.000000 5.324992 2.516569
H -0.000000 7.482502 1.232246
H 0.000000 7.482502 -1.232246
H 0.000000 5.324992 -2.516569
C -0.000000 2.738183 -1.087833
C -0.000000 2.738183 1.087833
N -0.000000 1.921109 0.000000
N -0.000000 2.386536 -2.376828
C -0.000000 1.137087 -2.772719
C -0.000000 0.701637 -4.165731
C -0.000000 -0.701637 -4.165731
C 0.000000 -1.427303 -5.350909
C 0.000000 -0.701262 -6.542954
C 0.000000 0.701262 -6.542954
C 0.000000 1.427303 -5.350909
C -0.000000 -1.137087 -2.772719
H 0.000000 -2.517023 -5.340610
H 0.000000 -1.233206 -7.495162
H 0.000000 1.233206 -7.495162
H 0.000000 2.517023 -5.340610
N -0.000000 0.000000 -2.005311
H -0.000000 0.000000 -0.989646
N -0.000000 -2.386536 -2.376828
C -0.000000 -2.738183 -1.087833
C -0.000000 -4.154569 -0.698058
C -0.000000 -4.154569 0.698058
C -0.000000 -5.333386 1.426609
C -0.000000 -6.530758 0.699239
C -0.000000 -6.530758 -0.699239
C -0.000000 -5.333386 -1.426609
C -0.000000 -2.738183 1.087833
H -0.000000 -5.324992 2.516569
H -0.000000 -7.482502 1.232246
H -0.000000 -7.482502 -1.232246
H -0.000000 -5.324992 -2.516569
N -0.000000 -1.921109 0.000000
N -0.000000 -2.386536 2.376828
C -0.000000 -1.137087 2.772719
C -0.000000 -0.701637 4.165731
C -0.000000 0.701637 4.165731
C 0.000000 1.427303 5.350909
C 0.000000 0.701262 6.542954
C -0.000000 -0.701262 6.542954
C -0.000000 -1.427303 5.350909
C -0.000000 1.137087 2.772719
H 0.000000 2.517023 5.340610
H 0.000000 1.233206 7.495162
H -0.000000 -1.233206 7.495162
H -0.000000 -2.517023 5.340610
N -0.000000 -0.000000 2.005311
N -0.000000 2.386536 2.376828
H -0.000000 -0.000000 0.989646

C0b
E = -1667.197693
C -0.000000 4.793111 -2.869932
C -0.000000 3.465653 -2.476113
C -0.000000 2.421854 -3.403265
C -0.000000 2.661542 -4.766211
C -0.000000 4.002222 -5.177416
C -0.000000 5.046318 -4.248776
C -0.000000 2.807121 -1.162021
N -0.000000 1.455619 -1.278943
C -0.000000 1.175063 -2.608725
N -0.000000 -0.000000 -3.221345
C -0.000000 -1.175063 -2.608725
C -0.000000 -2.421854 -3.403265
C -0.000000 -3.465653 -2.476113
C -0.000000 -2.807121 -1.162021
N -0.000000 -1.455619 -1.278943
C -0.000000 -2.661542 -4.766211
C -0.000000 -4.002222 -5.177416
C -0.000000 -5.046318 -4.248776
C -0.000000 -4.793111 -2.869932
N 0.000000 -3.498592 -0.017684
C 0.000000 -2.863568 1.134982
C 0.000000 -3.479545 2.452155
C 0.000000 -2.437217 3.392242
C 0.000000 -1.178269 2.658549
N 0.000000 -1.501436 1.321117
C 0.000000 -4.813628 2.843976
C 0.000000 -5.074891 4.214029
C 0.000000 -4.033530 5.154993
C 0.000000 -2.696283 4.759330
N 0.000000 0.000000 3.246851
C 0.000000 1.178269 2.658549
N 0.000000 1.501436 1.321117
C 0.000000 2.863568 1.134982
C 0.000000 3.479545 2.452155
C 0.000000 2.437217 3.392242
C 0.000000 4.813628 2.843976
C 0.000000 5.074891 4.214029
C 0.000000 4.033530 5.154993
C 0.000000 2.696283 4.759330
N 0.000000 3.498592 -0.017684
H -0.000000 5.601726 -2.138956
H -0.000000 6.077227 -4.605282
H -0.000000 4.236881 -6.242738
H -0.000000 1.840714 -5.483305
H -0.000000 -5.601726 -2.138956
H -0.000000 -6.077227 -4.605282
H -0.000000 -4.236881 -6.242738
H -0.000000 -1.840714 -5.483305
H 0.000000 -1.882599 5.484189
H 0.000000 -4.278311 6.217908
H 0.000000 -6.107838 4.564125
H 0.000000 -5.614269 2.104675
H 0.000000 5.614269 2.104675
H 0.000000 6.107838 4.564125
H 0.000000 4.278311 6.217908
H 0.000000 1.882599 5.484189
H -0.000000 0.939967 0.451938
H -0.000000 -0.939967 0.451938

C0c
E = -1667.185372
N 0.036577 1.963926 -0.000000
C 1.187471 2.724201 -0.000000
C 0.784451 4.117940 -0.000000
C -0.616869 4.145533 -0.000000
C -1.076377 2.767125 -0.000000
C -1.321910 5.345814 -0.000000
C -0.574654 6.521967 -0.000000
C 0.830293 6.495065 -0.000000
C 1.533502 5.292348 -0.000000
N 2.457918 2.341230 -0.000000
C 2.822359 1.077276 -0.000000
C 4.225724 0.637983 -0.000000
C 4.178922 -0.762779 0.000000
C 2.736069 -1.070718 0.000000
N 1.971015 -0.003488 0.000000
C 5.430596 1.322620 -0.000000
C 6.603946 0.560302 -0.000000
C 6.559664 -0.836943 0.000000
C 5.339907 -1.523571 0.000000
N 2.261125 -2.351585 0.000000

C 0.960396 -2.754759 0.000000
C 0.568044 -4.183619 0.000000
C -0.829539 -4.137403 0.000000
C -1.185854 -2.696392 0.000000
N -0.045743 -1.920169 0.000000
C 1.252592 -5.388352 0.000000
C 0.485951 -6.562263 0.000000
C -0.909013 -6.514148 0.000000
C -1.592763 -5.290297 0.000000
N -2.426335 -2.294751 0.000000
C -2.762266 -0.984768 0.000000
C -4.189150 -0.593731 0.000000
C -4.191000 0.803270 -0.000000
C -2.765857 1.180053 -0.000000
N -1.957155 0.081288 -0.000000
C -5.365243 -1.322226 0.000000
C -6.565714 -0.595603 0.000000
C -6.567286 0.800865 -0.000000
C -5.368581 1.530002 -0.000000
N -2.360773 2.438434 -0.000000
H -5.362366 2.620087 -0.000000
H -7.519451 1.333210 -0.000000
H -7.516944 -1.129660 0.000000
H -5.354652 -2.412201 0.000000
H 2.342882 -5.450405 0.000000
H 0.989038 -7.529855 0.000000
H -1.474226 -7.447009 0.000000
H -2.681584 -5.240221 0.000000
H 5.455758 2.412302 -0.000000
H 7.572366 1.062089 -0.000000
H 7.492217 -1.402250 0.000000
H 5.330820 -2.615636 0.000000
H -2.411597 5.353502 -0.000000
H -1.087105 7.484846 -0.000000
H 1.378770 7.437955 -0.000000
H 2.622760 5.261537 -0.000000
H 0.000000 0.934412 -0.000000
H 2.960059 -3.085654 0.000000

C0d
E = -1667.171526
N 0.355862 1.860037 0.272602
C -0.599178 2.799239 0.414858
C 0.016033 4.080698 0.625417
C 1.406076 3.857651 0.558433
C 1.617830 2.450072 0.321341
C 2.312516 4.909690 0.696581
C 1.795726 6.180771 0.912029
C 0.406857 6.405341 0.984392
C -0.500195 5.364966 0.839967
N -1.936875 2.584450 0.294683
C -2.556450 1.402077 -0.003470
C -3.970413 1.314896 -0.420680
C -4.149892 -0.045444 -0.686400
C -2.850018 -0.698731 -0.397446
N -1.922245 0.267509 0.026338
C -4.999965 2.229445 -0.586496
C -6.232360 1.732836 -1.027694
C -6.410742 0.372650 -1.292420
C -5.364495 -0.542733 -1.124200
N -2.667370 -1.966329 -0.485118
C -1.453123 -2.534061 -0.221948
C -1.367956 -3.864096 0.429247
C 0.000386 -4.118403 0.534705
C 0.656495 -2.913346 -0.020667
N -0.284672 -2.024527 -0.529528
C -2.313300 -4.763625 0.886016
C -1.835649 -5.952079 1.460260
C -0.467337 -6.205301 1.568968
C 0.482219 -5.280814 1.107662
N 1.937420 -2.726154 0.018661
C 2.512363 -1.525661 -0.156076
C 3.926174 -1.395326 -0.567683
C 4.206176 -0.027722 -0.491654
C 2.943371 0.592578 -0.055034
N 1.965693 -0.342665 0.138539
C 4.886219 -2.314654 -0.951950
C 6.156426 -1.814336 -1.273194
C 6.435612 -0.447633 -1.201103
C 5.457120 0.475411 -0.805324
N 2.798802 1.894835 0.110521
H 5.671008 1.542563 -0.743211
H 7.435611 -0.093587 -1.455493
H 6.942646 -2.504136 -1.583021
H 4.663355 -3.380766 -1.000503
H -3.380997 -4.560151 0.801783
H -2.546400 -6.692858 1.829270
H -0.131565 -7.140082 2.019956
H 1.552656 -5.470531 1.190325
H -4.880996 3.296663 -0.388517
H -7.067079 2.420327 -1.168468
H -7.383877 0.020344 -1.636677
H -5.493385 -1.605592 -1.329240
H 3.384806 4.725179 0.636159
H 2.475145 7.025943 1.028392
H 0.039201 7.417122 1.157864
H -1.572289 5.561224 0.903410
H 0.202829 0.846579 0.152799
H -2.516122 3.415540 0.319656

C0e
E = -1667.142488
N 1.402948 1.384490 -0.096730
C 2.730187 1.154798 0.117493
C 3.436314 2.409264 0.459792
C 2.452362 3.407191 0.454306
C 1.218448 2.670689 0.105378
C 2.759929 4.725477 0.751784
C 4.097560 5.024759 1.046223
C 5.077231 4.030262 1.046604
C 4.757097 2.697448 0.754096
N 3.368148 0.000624 0.000232
C 2.730572 -1.153838 -0.117223
C 3.437212 -2.407956 -0.459698
C 2.453660 -3.406291 -0.454318
C 1.219457 -2.670326 -0.105394
N 1.403471 -1.384017 0.096836
C 4.758113 -2.695580 -0.754028
C 5.078789 -4.028238 -1.046652
C 4.099522 -5.023136 -1.046376
C 2.761769 -4.724427 -0.751923
N 0.001579 -3.272007 0.002439
C -1.217965 -2.670185 0.110911
C -2.451913 -3.406986 0.456773
C -3.436547 -2.409428 0.458934
C -2.730471 -1.154913 0.118908
N -1.401695 -1.384524 -0.090841
C -2.759830 -4.725527 0.753685
C -4.097986 -5.025511 1.044086
C -5.078349 -4.031464 1.041118
C -4.758187 -2.698707 0.749305
N -3.368099 -0.000653 -0.000228
C -2.730953 1.153796 -0.119240
C -3.437502 2.408132 -0.459001
C -2.453222 3.406027 -0.456797
C -1.218978 2.669625 -0.111041
N -1.402210 1.383929 0.090610
C -4.759262 2.696985 -0.749240
C -5.079903 4.029663 -1.040895
C -4.099887 5.024047 -1.043828
C -2.761605 4.724491 -0.753544
N 0.000339 3.271943 -0.002544
H -2.010016 5.516614 -0.770534
H -4.379357 6.051813 -1.278323
H -6.111659 4.297237 -1.272401
H -5.516370 1.912668 -0.749467

H -2.007965 -5.517388 0.770725
H -4.377086 -6.053348 1.278711
H -6.109997 -4.299374 1.272714
H -5.515569 -1.914655 0.749479
H 5.514657 -1.910728 -0.756669
H 6.109993 -4.295108 -1.281373
H 4.378904 -6.050773 -1.281521
H 2.010730 -5.517106 -0.766295
H 2.008572 5.517856 0.766053
H 4.376521 6.052534 1.281266
H 6.108323 4.297572 1.281316
H 5.513953 1.912896 0.756838
H 0.001333 -4.286484 -0.000477
H -0.000292 4.286414 0.000362

C0f
E = -1667.139552
N 1.991852 -0.117352 0.063268
C 2.750980 0.935553 -0.122541
C 4.096092 0.618793 -0.671373
C 4.068803 -0.769559 -0.840156
C 2.725164 -1.193963 -0.375539
C 5.149606 -1.461106 -1.354004
C 6.284645 -0.714614 -1.701605
C 6.313233 0.670338 -1.537898
C 5.209106 1.364302 -1.020809
N 2.349903 2.203689 0.134039
C 1.061974 2.642500 0.351437
C 0.707098 3.991836 0.771174
C -0.707086 3.991839 0.771169
C -1.061964 2.642504 0.351430
N 0.000005 1.873491 0.117137
C 1.421854 5.129187 1.137978
C 0.700489 6.268818 1.495079
C -0.700473 6.268820 1.495074
C -1.421840 5.129193 1.137968
N -2.349894 2.203698 0.134024
C -2.750976 0.935563 -0.122550
C -4.096090 0.618805 -0.671378
C -4.068806 -0.769548 -0.840154
C -2.725165 -1.193952 -0.375543
N -1.991851 -0.117345 0.063263
C -5.209103 1.364316 -1.020814
C -6.313234 0.670353 -1.537895
C -6.284651 -0.714600 -1.701594
C -5.149614 -1.461094 -1.353992
N -2.336944 -2.432786 -0.312915
C -1.071163 -2.788440 -0.112416
C -0.698214 -3.957574 0.722704
C 0.698202 -3.957575 0.722703
C 1.071151 -2.788443 -0.112419
N -0.000004 -2.172791 -0.644882
C -1.426061 -4.896409 1.425259
C -0.696693 -5.865601 2.137108
C 0.696680 -5.865602 2.137108
C 1.426049 -4.896410 1.425258
N 2.336935 -2.432794 -0.312925
H 2.516278 -4.890445 1.424368
H 1.231070 -6.632392 2.699537
H -1.231083 -6.632391 2.699538
H -2.516290 -4.890442 1.424371
H -5.251347 2.449763 -0.909546
H -7.209333 1.224973 -1.818775
H -7.159819 -1.222682 -2.108406
H -5.116331 -2.543235 -1.482527
H 2.513423 5.145466 1.160369
H 1.234982 7.173667 1.786170
H -1.234964 7.173672 1.786161
H -2.513408 5.145476 1.160352
H 5.116319 -2.543246 -1.482544
H 7.159810 -1.222697 -2.108423
H 7.209333 1.224957 -1.818779
H 5.251353 2.449748 -0.909537
H -3.061888 2.922883 0.078985
H 3.061901 2.922871 0.079005

C1a
E = -1743.605991
C 2.833250 4.862362 -0.020098
C 2.562395 3.503304 -0.012449
C 3.583637 2.551451 -0.008521
C 4.920523 2.916368 -0.012054
C 5.204742 4.287951 -0.019768
C 4.181724 5.241631 -0.023750
C 1.311760 2.732531 -0.006625
N 1.548273 1.395182 -0.000315
C 2.902772 1.250363 -0.001469
N 3.602438 0.113161 0.005595
C 3.030659 -1.066701 0.003526
C 3.738056 -2.341872 0.027005
C 2.768984 -3.356816 0.019732
C 1.461257 -2.710625 -0.007646
N 1.690085 -1.359293 -0.018547
C 5.096275 -2.635644 0.053655
C 5.456054 -3.983807 0.072236
C 4.487302 -4.998301 0.064908
C 3.124338 -4.700320 0.038796
N 0.313764 -3.344645 -0.019720
C -0.866801 -2.723583 -0.020883
C -2.111762 -3.496623 -0.058280
C -3.143587 -2.553969 -0.040320
C -2.463729 -1.247448 0.009908
N -1.112935 -1.383501 0.014573
C -2.360339 -4.858129 -0.114478
C -3.701815 -5.258280 -0.156373
C -4.735136 -4.317944 -0.143929
C -4.476415 -2.941250 -0.086243
N -3.159333 -0.100134 0.038854
C -2.594185 1.087679 0.029137
N -1.253783 1.371356 0.028753
C -1.016300 2.724272 0.000960
C -2.319943 3.375137 -0.018264
C -3.300366 2.369192 0.000276
C -2.653877 4.722387 -0.059557
C -4.012364 5.041457 -0.083197
C -4.991901 4.039943 -0.068084
C -4.656530 2.684420 -0.027081
N 0.129729 3.356387 -0.009613
H 2.030458 5.599633 -0.023344
H 4.441313 6.301089 -0.029929
H 6.243457 4.621024 -0.022797
H 5.711899 2.166849 -0.008948
H 2.364136 -5.481091 0.033087
H 4.808798 -6.040436 0.079926
H 6.511745 -4.257199 0.092853
H 5.841690 -1.840735 0.059370
H 0.963458 -0.649987 -0.042625
H -5.291149 -2.214214 -0.077883
H -5.769643 -4.661686 -0.182352
H -3.945542 -6.320518 -0.202578
H -1.544048 -5.580288 -0.128438
H -1.879906 5.489366 -0.075029
H -4.318725 6.087757 -0.116478
H -6.044539 4.324700 -0.090900
H -5.424580 1.907544 -0.014299
H -0.531620 0.657409 0.056170
H -5.106871 -0.162723 0.311873
O -6.080834 -0.158490 0.352765
H -6.273406 -0.212975 1.296732

C1b
E = -1743.604406
C -5.345886 -1.423108 0.378254
C -4.170862 -0.704580 0.226356
C -4.169972 0.690475 0.141038
C -5.343535 1.424118 0.204863

C -6.536163 0.706350 0.363375
C -6.537250 -0.689401 0.448425
C -2.758204 -1.100291 0.106327
N -1.950018 -0.014736 -0.030747
C -2.756114 1.067616 -0.022337
N -2.389662 2.349638 -0.150513
C -1.135979 2.718177 -0.239112
C -0.700041 4.106238 -0.385237
C 0.700501 4.106168 -0.385283
C 1.136305 2.718063 -0.239183
N 0.000131 1.948897 -0.186110
C -1.426390 5.284489 -0.501517
C -0.700719 6.470973 -0.622217
C 0.701405 6.470902 -0.622263
C 1.426963 5.284345 -0.501609
N 2.389957 2.349394 -0.150633
C 2.756257 1.067333 -0.022467
C 4.170069 0.690040 0.141013
C 4.170801 -0.705012 0.226323
C 2.758101 -1.100567 0.106193
N 1.950052 -0.014933 -0.031014
C 5.343701 1.423558 0.204946
C 6.536240 0.705659 0.363556
C 6.537170 -0.690090 0.448597
C 5.345730 -1.423669 0.378319
N 2.390390 -2.379072 0.081051
C 1.133262 -2.760044 0.026394
N -0.000092 -2.011450 0.224626
C -1.133543 -2.759942 0.026478
C -0.701279 -4.114309 -0.296279
C 0.700851 -4.114371 -0.296336
C -1.427634 -5.261273 -0.596990
C -0.702053 -6.416607 -0.887351
C 0.701384 -6.416668 -0.887406
C 1.427086 -5.261397 -0.597102
N -2.390625 -2.378840 0.081172
H -5.339912 -2.511602 0.437981
H -7.485402 -1.215234 0.568830
H -7.483466 1.244390 0.419173
H -5.334884 2.511774 0.132655
H 2.516701 5.272917 -0.498763
H 1.233412 7.418260 -0.718273
H -1.232637 7.418385 -0.718194
H -2.516130 5.273173 -0.498602
H 0.000107 0.936589 -0.062115
H 5.339631 -2.512163 0.438045
H 7.485254 -1.216027 0.569082
H 7.483596 1.243596 0.419441
H 5.335172 2.511216 0.132750
H -2.517368 -5.248993 -0.601434
H -1.233558 -7.339794 -1.121788
H 1.232790 -7.339902 -1.121885
H 2.516820 -5.249209 -0.601631
H -0.000035 -1.070213 0.634091
H -0.763894 0.096280 2.791371
O -0.000119 0.097406 2.201895
H 0.762625 0.095958 2.792702

C2a
E = -1820.003400
C 1.536977 -5.206382 -0.876519
C 0.797573 -4.082482 -0.523716
C -0.603968 -4.079094 -0.582065
C -1.315259 -5.200063 -0.997241
C -0.576816 -6.332227 -1.340348
C 0.825655 -6.335580 -1.281064
C 1.213137 -2.757572 -0.082025
N 0.079029 -2.019140 0.117116
C -1.045588 -2.750499 -0.180646
N 2.470305 -2.390161 0.076698
C 2.842519 -1.120376 0.149110
C 4.238655 -0.729308 0.415349
C 4.251572 0.665202 0.325510
C 2.861204 1.045322 0.012994
N 2.057927 -0.028391 -0.073810
C 5.388009 -1.450826 0.691644
C 6.568525 -0.720273 0.884358
C 6.581142 0.674468 0.794732
C 5.413606 1.395450 0.508767
N 2.514935 2.328169 -0.171812
C 1.270858 2.694827 -0.349888
C 0.854926 4.076914 -0.596155
C -0.543233 4.086709 -0.661504
C -0.999050 2.710434 -0.455644
N 0.129077 1.936616 -0.311280
C 1.597992 5.240340 -0.744259
C 0.890979 6.423906 -0.966570
C -0.509029 6.433933 -1.032443
C -1.251407 5.261131 -0.878191
N -2.255212 2.358581 -0.385535
C -2.636221 1.078591 -0.238639
C -4.042631 0.725338 0.028932
C -4.060104 -0.671422 0.110977
C -2.663020 -1.086541 -0.104683
N -1.856512 -0.009983 -0.311532
C -5.194854 1.477865 0.200118
C -6.381521 0.780256 0.461309
C -6.399598 -0.616364 0.539167
C -5.231594 -1.369535 0.361682
N -2.303058 -2.363391 -0.133008
O -0.146100 0.042644 1.981657
H -5.241032 -2.458865 0.412810
H -7.343239 -1.126882 0.736928
H -7.310927 1.334030 0.601288
H -5.173977 2.565728 0.129383
H 2.686190 5.220520 -0.688454
H 1.436409 7.360252 -1.090990
H -1.026728 7.378035 -1.207012
H -2.340202 5.258473 -0.925516
H 0.118833 0.933823 -0.118496
H 5.372280 -2.539121 0.753815
H 7.496860 -1.248717 1.105607
H 7.519083 1.209983 0.948020
H 5.416282 2.482803 0.431645
H -2.403876 -5.186569 -1.047592
H -1.096951 -7.234745 -1.663967
H 1.367229 -7.240436 -1.559712
H 2.625926 -5.196372 -0.833406
H 0.056641 -1.117281 0.628241
H -1.053999 0.022461 2.352768
H 0.439923 0.014141 2.746304
O -2.717540 -0.084304 2.955056
H -3.370807 0.564214 2.662950
H -3.196836 -0.921146 2.925534

C2b
E = -1819.998553
C 5.376548 -1.106332 0.070908
C 4.177923 -0.397884 0.031544
C 4.163140 1.006524 0.067567
C 5.333937 1.750201 0.138669
C 6.536322 1.042806 0.174892
C 6.553816 -0.357821 0.141970
C 2.783070 -0.836835 -0.024801
N 2.009491 0.292929 -0.023726
C 2.772229 1.434893 0.028314
N 2.374915 -2.087598 -0.056701
C 1.085205 -2.451070 -0.029289
C 0.696447 -3.869203 -0.016466
C -0.702627 -3.867968 0.016254
C -1.088931 -2.449178 0.029329
N -0.001187 -1.632659 0.000084
C 1.423715 -5.051559 -0.023126
C 0.692423 -6.247349 -0.009600
C -0.702974 -6.246097 0.008815
C -1.432068 -5.048973 0.022623

N -2.378078 -2.083604 0.056795
C -2.784230 -0.832215 0.024906
C -4.178431 -0.391147 -0.031494
C -4.161570 1.013231 -0.067546
C -2.770010 1.439506 -0.028247
N -2.008975 0.296385 0.023842
C -5.378068 -1.097847 -0.070923
C -6.554233 -0.347603 -0.142062
C -6.534680 1.052992 -0.175006
C -5.331260 1.758631 -0.138726
N -2.371948 2.686842 -0.040556
C -1.083898 3.038948 -0.018174
C -0.694266 4.454810 -0.012762
C 0.701433 4.453639 0.012727
C 1.088697 3.037120 0.018286
N 0.001701 2.220253 0.000016
C -1.422080 5.634119 -0.026129
C -0.693630 6.830512 -0.012964
C 0.704800 6.829336 0.012723
C 1.431233 5.631718 0.025982
N 2.376132 2.682877 0.040627
O 4.480626 -4.109756 -0.472983
H 2.520984 5.622549 0.046076
H 1.238114 7.780823 0.022578
H -1.225342 7.782895 -0.022898
H -2.511845 5.626789 -0.046219
H -5.400013 -2.189596 -0.039061
H -7.512641 -0.867292 -0.174265
H -7.477094 1.599180 -0.231774
H -5.298817 2.847509 -0.167162
H -0.994202 0.289398 0.069831
H 2.515306 -5.059108 -0.041895
H 1.227777 -7.197724 -0.014654
H -1.240044 -7.195504 0.013639
H -2.523670 -5.054437 0.041435
H 5.303089 2.839126 0.167084
H 7.479540 1.587612 0.231599
H 7.511459 -0.878921 0.174124
H 5.396910 -2.198113 0.039114
H 0.994704 0.287220 -0.069632
H 3.763440 -3.451432 -0.430542
H 4.604808 -4.261762 -1.417738
H -3.768924 -3.445379 0.431829
O -4.487374 -4.102323 0.473986
H -4.611801 -4.254626 1.418660

C2c
E = -1819.997836
C 3.205282 4.792546 0.062384
C 2.896664 3.441469 0.049147
C 3.889115 2.461193 0.105969
C 5.233790 2.789210 0.178617
C 5.555902 4.152085 0.192748
C 4.561521 5.134187 0.135731
C 1.626604 2.706575 -0.018776
N 1.824610 1.363732 -0.005124
C 3.172981 1.179738 0.068720
N 3.838649 0.023571 0.109621
C 3.239023 -1.141836 0.069061
C 3.913090 -2.433452 0.130780
C 2.922549 -3.425352 0.064076
C 1.634604 -2.748487 -0.038191
N 1.895229 -1.403379 -0.033523
C 5.260220 -2.759378 0.236933
C 5.586727 -4.115345 0.273378
C 4.596475 -5.106930 0.206589
C 3.245013 -4.777010 0.101075
N 0.473763 -3.355529 -0.117279
C -0.686701 -2.701940 -0.179032
C -1.952205 -3.435783 -0.290445
C -2.952402 -2.460350 -0.319796
C -2.236044 -1.177057 -0.223489
N -0.892862 -1.354619 -0.146663
C -2.243600 -4.787273 -0.372901
C -3.594373 -5.139327 -0.491717
C -4.594564 -4.164183 -0.526032
C -4.293767 -2.797094 -0.440899
N -2.900278 -0.013128 -0.228792
C -2.306679 1.158096 -0.202985
C -2.982640 2.453224 -0.278286
C -1.983222 3.439482 -0.242726
C -0.696014 2.761283 -0.142100
N -0.963369 1.413484 -0.122210
C -2.290053 4.791826 -0.312441
C -3.639469 5.133083 -0.421048
C -4.636292 4.149311 -0.459312
C -4.329390 2.787808 -0.389728
N 0.463590 3.364534 -0.087793
O -5.870059 0.009296 -0.051793
O -6.637262 -0.126679 2.661076
H 2.424423 5.551675 0.017188
H 4.850318 6.186022 0.148493
H 6.602037 4.455939 0.249079
H 6.002775 2.017975 0.222520
H 2.468334 -5.539632 0.048830
H 4.892045 -6.156363 0.237935
H 6.632700 -4.413432 0.355436
H 6.022222 -1.982052 0.288583
H -5.079504 -2.037248 -0.466133
H -5.636168 -4.472897 -0.624998
H -3.871141 -6.192176 -0.562135
H -1.453675 -5.537977 -0.349744
H -1.503942 5.546063 -0.285822
H -3.924330 6.184397 -0.479941
H -5.680006 4.453408 -0.548629
H -5.107674 2.019151 -0.415188
H -0.256028 0.688477 -0.047407
H -4.901681 -0.022069 0.010219
H -6.161165 -0.032711 0.878903
H -7.139055 -0.922057 2.876518
H -7.214688 0.592794 2.943518
H 1.184582 -0.680780 -0.100979

C3a
E = -1896.399573
C 3.543524 -3.567709 0.210313
C 2.278894 -2.986811 0.156780
C 1.120778 -3.780931 0.115999
C 1.179087 -5.168108 0.129128
C 2.445201 -5.751828 0.184906
C 3.602113 -4.963446 0.225004
C -0.029459 -2.888632 0.063862
N 0.468065 -1.608033 0.071802
C 1.837668 -1.589641 0.128440
N 2.618926 -0.530742 0.151391
C 2.146484 0.726633 0.122795
C 3.054692 1.886114 0.128207
C 2.218086 3.005175 0.118916
C 0.852216 2.476758 0.095809
N 0.843899 1.112781 0.097075
C 4.433153 2.023436 0.139474
C 4.950689 3.325225 0.153840
C 4.112003 4.441512 0.148388
C 2.717976 4.296824 0.127615
N -0.190243 3.308705 0.076238
C -1.437143 2.904954 0.043935
C -2.596752 3.790441 0.014735
C -3.741939 2.980422 -0.022588
C -3.291919 1.593365 -0.016032
N -1.920071 1.623516 0.026129
C -2.688449 5.177365 0.017254
C -3.968645 5.731140 -0.018184
C -5.113514 4.921215 -0.055491
C -5.018448 3.529169 -0.058630
N -4.077046 0.544229 -0.047914
C -3.603450 -0.704416 -0.043119

C -4.514813 -1.854944 -0.082740
C -3.689482 -2.980748 -0.063633
C -2.317829 -2.457268 -0.013088
N -2.300462 -1.100146 -0.003245
C -5.895749 -1.964652 -0.129810
C -6.429912 -3.258927 -0.157835
C -5.602912 -4.386936 -0.138644
C -4.208539 -4.265544 -0.090640
N -1.273714 -3.291025 0.020982
O 5.564146 -1.131053 0.261521
O 6.530797 0.097799 -1.958243
O 7.445762 0.868290 0.492875
H -3.557222 -5.139378 -0.075112
H -6.055095 -5.379357 -0.161507
H -7.511902 -3.392420 -0.195287
H -6.533487 -1.080823 -0.144231
H -1.793007 5.797789 0.045831
H -4.084557 6.815675 -0.017159
H -6.097002 5.392049 -0.082785
H -5.901805 2.891688 -0.087895
H 5.106904 1.173119 0.131138
H 6.033802 3.450511 0.172118
H 4.548647 5.441119 0.158949
H 2.050854 5.158787 0.119781
H 0.267666 -5.764610 0.097120
H 2.537308 -6.838614 0.197848
H 4.576740 -5.451262 0.268720
H 4.453159 -2.964238 0.242474
H -0.095399 -0.762975 0.045411
H 4.630548 -0.868810 0.344720
H 5.781947 -0.902973 -0.670613
H -1.345424 0.785968 0.043819
H 7.107534 0.519797 -1.288855
H 7.135076 -0.286956 -2.602130
H 6.900561 0.097227 0.751850
H 8.271373 0.778173 0.979577

C3b
E = -1896.398729
C -0.439055 -5.321010 -1.044486
C -0.706484 -3.994200 -0.729383
C -2.008188 -3.477699 -0.771125
C -3.090417 -4.269915 -1.134602
C -2.827962 -5.605442 -1.443060
C -1.524760 -6.122731 -1.398775
C 0.178027 -2.901662 -0.335829
N -0.592977 -1.789419 -0.175800
C -1.920474 -2.066241 -0.413022
N 1.479418 -3.032423 -0.172071
C 2.294178 -1.989387 -0.068878
C 3.726894 -2.155475 0.242810
C 4.257259 -0.863776 0.222537
C 3.116656 0.016126 -0.098106
N 1.978902 -0.683944 -0.269829
C 4.514251 -3.261308 0.511969
C 5.873457 -3.028803 0.767125
C 6.403813 -1.736581 0.746906
C 5.597087 -0.623107 0.470899
N 3.270319 1.340017 -0.203194
C 2.248546 2.150768 -0.345275
C 2.374617 3.598670 -0.507916
C 1.079228 4.127394 -0.559773
C 0.145474 3.008735 -0.430023
N 0.910377 1.865368 -0.332433
C 3.497063 4.412102 -0.595615
C 3.280332 5.783072 -0.743468
C 1.983277 6.312923 -0.796350
C 0.858499 5.491214 -0.703000
N -1.153596 3.140622 -0.381274
C -1.984665 2.089605 -0.306095
C -3.431078 2.263522 -0.098323
C -3.958179 0.965915 -0.089338
C -2.802364 0.076953 -0.289292
N -1.653331 0.788134 -0.409224
C -4.241409 3.376873 0.069948
C -5.610034 3.150551 0.256256
C -6.137902 1.853730 0.268060
C -5.316166 0.734322 0.091720
N -2.941873 -1.247246 -0.366400
O -1.504119 -2.098453 2.708968
H -5.720364 -0.278536 0.088336
H -7.210783 1.716092 0.410073
H -6.281584 3.999691 0.391157
H -3.824688 4.384188 0.054939
H 4.499983 3.988132 -0.550067
H 4.134379 6.457346 -0.818660
H 1.853126 7.389746 -0.912120
H -0.153466 5.893901 -0.741126
H 0.536981 0.922556 -0.216255
H 4.093666 -4.266987 0.522411
H 6.530986 -3.871856 0.983751
H 7.466257 -1.593012 0.948471
H 6.003630 0.388097 0.450984
H -4.098801 -3.858282 -1.173008
H -3.651084 -6.262542 -1.727076
H -1.359771 -7.171521 -1.648937
H 0.578491 -5.710096 -1.012127
H -0.276297 -0.943992 0.328291
H -2.164307 -1.398213 2.854528
H -1.643586 -2.729501 3.423192
O -2.724619 0.449483 2.827101
H -3.483872 0.721896 2.291504
H -2.813084 0.948770 3.648432
H -0.825924 0.480664 2.060818
O 0.016312 0.019034 1.916849
H -0.148281 -0.805628 2.411745

C3c
E = -1896.392676
C -3.419331 -3.604806 -0.181669
C -2.139629 -3.064129 -0.146525
C -0.984773 -3.861824 -0.112304
C -1.069762 -5.247814 -0.110990
C -2.351570 -5.804317 -0.145940
C -3.499948 -5.000887 -0.180145
C 0.165395 -2.959154 -0.084707
N -0.335745 -1.681765 -0.108381
C -1.706180 -1.672724 -0.143136
N -2.513545 -0.646784 -0.174003
C -2.092696 0.618780 -0.172931
C -3.070625 1.707243 -0.251979
C -2.325708 2.886182 -0.216871
C -0.921174 2.450031 -0.124080
N -0.823484 1.088824 -0.101436
C -4.450590 1.697451 -0.368155
C -5.083140 2.946247 -0.435997
C -4.343101 4.134149 -0.392598
C -2.944705 4.125533 -0.284101
N 0.078103 3.330592 -0.077310
C 1.338512 2.958524 -0.020287
C 2.478494 3.865476 0.022712
C 3.638948 3.076468 0.066596
C 3.218508 1.680206 0.050668
N 1.847460 1.685394 0.002267
C 2.544900 5.254502 0.021365
C 3.813519 5.832188 0.067040
C 4.973491 5.043533 0.111268
C 4.904613 3.650451 0.111114
N 4.034375 0.651890 0.075239
C 3.620754 -0.615118 0.050923
C 4.588439 -1.720453 0.075784
C 3.821369 -2.886347 0.039528
C 2.424158 -2.433915 -0.004988
N 2.339004 -1.078665 0.002280
C 5.973042 -1.761099 0.123449
C 6.572268 -3.026797 0.133614

C 5.803464 -4.194877 0.097210
C 4.404776 -4.143471 0.049288
N 1.422340 -3.323495 -0.042133
O -5.877468 -1.487238 0.028304
O -6.386819 0.585480 2.089151
H 3.797786 -5.048381 0.020605
H 6.305325 -5.163391 0.106270
H 7.659646 -3.105782 0.170436
H 6.564953 -0.846232 0.151367
H 1.638097 5.857874 -0.014606
H 3.909123 6.918725 0.067791
H 5.947734 5.532832 0.145735
H 5.799600 3.029550 0.143938
H -5.005041 0.757686 -0.400591
H -6.169193 2.970480 -0.539368
H -4.866594 5.089596 -0.453832
H -2.368379 5.050578 -0.259509
H -0.173809 -5.867709 -0.085249
H -2.462053 -6.889662 -0.147394
H -4.480600 -5.477973 -0.207605
H -4.304528 -2.959216 -0.204900
H 0.252867 -0.854251 -0.097210
H -6.526939 -0.939024 -0.443336
H -5.874153 -1.064131 0.898841
H -5.681990 1.246110 2.114906
H -6.709137 0.529918 2.996739
H 1.263832 0.853768 -0.013690
H -8.468480 0.791522 -0.616230
O -7.518292 0.778317 -0.459414
H -7.417296 0.817283 0.507993

C3d
E = -1896.391255
C 4.896313 -2.859518 -0.014484
C 3.538047 -2.550593 -0.002477
C 2.562721 -3.560715 -0.057254
C 2.903643 -4.905740 -0.120637
C 4.263614 -5.217902 -0.129726
C 5.238170 -4.212308 -0.078082
C 2.824976 -1.274696 0.038719
N 1.486691 -1.563392 0.011846
C 1.257833 -2.916044 -0.042948
N 3.382812 -0.083056 0.079501
C 2.687103 1.060674 0.041677
C 3.367091 2.363429 0.028165
C 2.341413 3.314165 -0.023110
C 1.093012 2.536381 -0.041536
N 1.332855 1.199948 0.000199
C 4.704747 2.733527 0.048007
C 4.983512 4.107079 0.028288
C 3.960710 5.055720 -0.009133
C 2.611601 4.675711 -0.035833
N -0.100021 3.147015 -0.083108
C -1.253014 2.516984 -0.038405
C -2.564429 3.159750 0.004627
C -3.528942 2.137135 0.065693
C -2.811564 0.865601 0.052581
N -1.473025 1.164003 -0.007818
C -2.929295 4.502798 0.005500
C -4.295566 4.789300 0.060401
C -5.254915 3.772034 0.112537
C -4.889886 2.423525 0.116012
N -3.367825 -0.324688 0.078040
C -2.669643 -1.468998 0.020659
C -3.345978 -2.776379 -0.040515
C -2.311051 -3.714350 -0.091447
C -1.069785 -2.936684 -0.055249
N -1.318881 -1.598503 0.004538
C -4.677998 -3.168071 -0.074173
C -4.932008 -4.544535 -0.154227
C -3.895390 -5.480267 -0.200123
C -2.554956 -5.075678 -0.169815
N 0.113215 -3.551858 -0.076966
O 6.294790 -0.046966 0.521931
H -1.736370 -5.794253 -0.209533
H -4.135803 -6.542373 -0.263344
H -5.965610 -4.891874 -0.183445
H -5.495229 -2.444850 -0.036017
H -2.182522 5.298159 -0.047493
H -4.619751 5.830757 0.061208
H -6.312598 4.035712 0.151239
H -5.639725 1.630312 0.160750
H -0.741568 0.459457 -0.033532
H 5.508597 1.995374 0.081061
H 6.022470 4.439001 0.043298
H 4.213880 6.116695 -0.019123
H 1.816665 5.423080 -0.073217
H 2.133863 -5.675838 -0.163931
H 4.575645 -6.261836 -0.179874
H 6.292314 -4.492141 -0.089453
H 5.660378 -2.079924 0.030595
H 0.759400 -0.854753 0.039230
H 5.322023 -0.034820 0.466190
H 6.476286 -0.012638 1.469021
H -0.165052 5.082455 -0.568530
O -0.231608 6.053111 -0.614701
H -0.210417 6.249259 -1.559180
H -5.304647 -0.419284 0.466527
O -6.277120 -0.428404 0.527629
H -6.451998 -0.511916 1.472907

C4a
E = -1972.808315
C 1.625787 5.340982 -0.676730
C 1.679609 3.978859 -0.395414
C 2.901357 3.290000 -0.339721
C 4.111342 3.940119 -0.564496
C 4.059940 5.306411 -0.835110
C 2.837337 5.995781 -0.890301
C 0.626076 3.008219 -0.145171
N 1.230232 1.798573 0.061855
C 2.595867 1.896809 -0.056681
N -0.663999 3.294645 -0.149265
C -1.603525 2.370979 -0.231143
C -3.033679 2.716532 -0.195731
C -3.707397 1.511342 -0.396368
C -2.648549 0.495855 -0.530125
N -1.424218 1.027051 -0.434165
C -3.713347 3.909909 -0.025121
C -5.113501 3.856189 -0.055168
C -5.789020 2.649195 -0.256144
C -5.090202 1.447739 -0.434768
N -2.962589 -0.801866 -0.705414
C -2.049685 -1.736525 -0.755771
C -2.357251 -3.163397 -0.859647
C -1.146844 -3.856125 -0.770730
C -0.085193 -2.860812 -0.613290
N -0.686335 -1.623217 -0.681750
C -3.567554 -3.828323 -0.996492
C -3.525788 -5.223587 -1.047579
C -2.312148 -5.917952 -0.958195
C -1.098071 -5.241893 -0.814616
N 1.167878 -3.161639 -0.422666
C 2.112674 -2.223406 -0.223319
C 3.511538 -2.604611 0.031418
C 4.198931 -1.397922 0.185349
C 3.181851 -0.349327 0.014416
N 1.947355 -0.891769 -0.221202
C 4.157737 -3.827271 0.119485
C 5.534790 -3.803460 0.373750
C 6.223319 -2.595691 0.527633
C 5.562282 -1.364689 0.433366
N 3.495383 0.938107 0.038075
O -2.123402 1.428636 2.560575
H 6.093851 -0.419693 0.546855
H 7.296624 -2.615330 0.721801

H 6.083417 -4.743170 0.451114
H 3.614266 -4.763782 -0.006565
H -4.506561 -3.279101 -1.064304
H -4.454037 -5.785342 -1.159358
H -2.318251 -7.007874 -1.001783
H -0.148625 -5.771771 -0.740752
H -0.189974 -0.739012 -0.568902
H -3.179687 4.848459 0.125479
H -5.689272 4.773411 0.076487
H -6.879618 2.645120 -0.278407
H -5.611904 0.504959 -0.602409
H 5.054714 3.395749 -0.526816
H 4.986735 5.854245 -1.010493
H 2.839568 7.064717 -1.107304
H 0.671406 5.865057 -0.724100
H 0.750449 0.972659 0.439773
H -2.771100 0.683204 2.464430
H -2.416171 1.920616 3.334987
O -3.554357 -0.808681 2.273440
H -2.809350 -1.448977 2.267227
H -3.978961 -0.947510 1.417169
H 0.766580 0.281628 2.850399
O 0.132658 0.133882 2.137981
H -0.659244 0.691378 2.376864
H -0.614113 -1.489395 2.158114
O -1.225058 -2.257158 2.106676
H -0.967508 -2.842443 2.827336

C4b
E = -1972.802904
C 4.713545 -3.330435 0.159087
C 3.425878 -2.839796 -0.027660
C 2.316541 -3.698723 -0.091178
C 2.458352 -5.076889 0.029205
C 3.749327 -5.570979 0.214222
C 4.857822 -4.712536 0.278074
C 2.928933 -1.479839 -0.179837
N 1.570104 -1.572490 -0.339012
C 1.134409 -2.870896 -0.285789
N 3.667462 -0.393603 -0.156613
C 3.153578 0.832107 -0.227979
C 4.023205 2.014259 -0.177414
C 3.161740 3.111024 -0.228035
C 1.809743 2.542372 -0.310758
N 1.835924 1.187585 -0.323778
C 5.397255 2.170755 -0.086749
C 5.886622 3.482592 -0.052933
C 5.023115 4.581786 -0.103712
C 3.635471 4.412820 -0.190478
N 0.738748 3.348855 -0.338977
C -0.483334 2.898333 -0.430341
C -1.687739 3.723669 -0.391878
C -2.786948 2.860384 -0.482749
C -2.263236 1.500168 -0.573621
N -0.899565 1.600092 -0.584018
C -1.850390 5.096728 -0.274393
C -3.159331 5.583895 -0.251301
C -4.259041 4.720437 -0.341445
C -4.089163 3.338998 -0.458636
N -3.000707 0.420172 -0.632776
C -2.481297 -0.815574 -0.633852
C -3.353013 -1.989513 -0.746287
C -2.502400 -3.092429 -0.668915
C -1.154097 -2.537240 -0.517299
N -1.179088 -1.167405 -0.518057
C -4.723568 -2.134661 -0.885714
C -5.222224 -3.442081 -0.944346
C -4.369181 -4.547507 -0.866009
C -2.984513 -4.389317 -0.726163
N -0.094571 -3.327697 -0.391127
O -2.523269 -1.949665 2.364468
H -2.313216 -5.245627 -0.662584
H -4.792831 -5.551515 -0.915744

H -6.295422 -3.603325 -1.054164
H -5.384186 -1.269682 -0.948653
H -0.988297 5.759478 -0.202482
H -3.331478 6.657162 -0.161051
H -5.266686 5.137235 -0.319530
H -4.940829 2.662549 -0.528714
H -0.264632 0.809429 -0.591984
H 6.063242 1.308995 -0.043818
H 6.961915 3.652801 0.015717
H 5.440672 5.589070 -0.073618
H 2.955108 5.263592 -0.226937
H 1.591262 -5.735090 -0.020098
H 3.903709 -6.646255 0.312207
H 5.851455 -5.137857 0.424469
H 5.567984 -2.655961 0.209228
H 0.973084 -0.762562 -0.470566
H -3.172519 -1.204766 2.334879
H -2.887034 -2.574542 3.000382
O -4.009373 0.298271 2.177266
H -4.264161 0.410562 1.252205
H -3.278018 0.946546 2.285322
H -0.320968 -0.575396 1.326685
O -0.303272 -0.459295 2.286771
H -1.016185 -1.088776 2.546804
H -1.106131 1.051286 2.396272
O -1.716134 1.824051 2.283387
H -1.573969 2.375703 3.060443

C4c
E = -1972.802495
C -3.070511 -3.819154 -0.057183
C -1.844049 -3.159094 -0.036827
C -0.637623 -3.878613 -0.019762
C -0.608891 -5.266879 -0.021319
C -1.836482 -5.929445 -0.040900
C -3.041565 -5.215735 -0.059332
C 0.455147 -2.916102 -0.004270
N -0.122491 -1.668660 -0.008810
C -1.490764 -1.736864 -0.027549
N -2.338507 -0.728711 -0.031500
C -1.946008 0.555503 -0.019547
C -2.922929 1.658405 -0.001110
C -2.153255 2.825135 0.000446
C -0.758992 2.379808 -0.014674
N -0.667810 1.019597 -0.023509
C -4.306873 1.717211 0.021785
C -4.901171 2.987014 0.043870
C -4.127030 4.148431 0.041696
C -2.726791 4.085320 0.020120
N 0.230971 3.274634 -0.018081
C 1.500518 2.949011 -0.019671
C 2.602182 3.906603 -0.023112
C 3.797273 3.171787 -0.017531
C 3.436390 1.758354 -0.010595
N 2.063663 1.701506 -0.014149
C 2.603820 5.296363 -0.029281
C 3.846061 5.931725 -0.030260
C 5.041118 5.197145 -0.024705
C 5.036175 3.801771 -0.017968
N 4.285521 0.761314 -0.000390
C 3.889661 -0.515394 0.007690
C 4.870707 -1.607030 0.023647
C 4.116291 -2.781905 0.027638
C 2.714340 -2.344089 0.013256
N 2.613575 -0.990028 0.002583
C 6.256694 -1.630500 0.033842
C 6.870941 -2.888695 0.048594
C 6.115197 -4.066027 0.052563
C 4.715346 -4.031886 0.041912
N 1.722595 -3.239855 0.008970
O -5.220081 -1.513615 -0.198425
O -6.375969 -0.312320 1.905884
O -6.744764 0.007123 -1.846491

O -7.656447 1.400310 0.249332
H 4.119686 -4.944719 0.044692
H 6.628746 -5.028363 0.064093
H 7.959744 -2.954708 0.057105
H 6.838112 -0.708525 0.030460
H 1.669560 5.857349 -0.033217
H 3.891849 7.021460 -0.035317
H 5.992658 5.730330 -0.025553
H 5.959341 3.222717 -0.013290
H -4.924871 0.824651 0.022516
H -5.990925 3.034787 0.067699
H -4.620899 5.120976 0.059128
H -2.110042 4.984117 0.020636
H 0.338855 -5.804588 -0.007991
H -1.860149 -7.019966 -0.043044
H -3.984222 -5.764258 -0.076352
H -4.016207 -3.273493 -0.077740
H 0.385760 -0.788821 -0.002659
H -4.300823 -1.193814 -0.148368
H -5.628405 -1.210711 0.650057
H -8.584703 1.649855 0.197372
H -7.455286 0.934759 -0.600619
H 1.544253 0.828420 -0.013734
H -6.951732 -0.748077 2.542550
H -6.950003 0.340663 1.442616
H -6.175964 -0.641719 -1.369612
H -7.278439 -0.521929 -2.448408

C4d
E = -1972.790194
C 4.425029 4.352689 0.066939
C 3.932331 3.057172 0.061201
C 4.778124 1.949776 0.147514
C 6.153908 2.089629 0.243018
C 6.661416 3.394731 0.248737
C 5.813872 4.504329 0.162372
C 2.573938 2.504332 -0.020931
N 2.583188 1.148210 0.008313
C 3.891927 0.779863 0.111216
N 4.388763 -0.457155 0.178178
C 3.630827 -1.526183 0.133801
C 4.111810 -2.900717 0.205348
C 2.990881 -3.740252 0.113965
C 1.815794 -2.885756 -0.013779
N 2.265088 -1.592585 0.008216
C 5.396059 -3.416705 0.335536
C 5.523954 -4.805469 0.372567
C 4.403316 -5.644733 0.281221
C 3.115632 -5.124328 0.149652
N 0.583657 -3.321322 -0.135471
C -0.470549 -2.512458 -0.237437
C -1.817224 -3.064038 -0.414888
C -2.668621 -1.958358 -0.480523
C -1.792763 -0.786840 -0.324220
N -0.490913 -1.147867 -0.196252
C -2.291337 -4.360671 -0.526329
C -3.671563 -4.521450 -0.712452
C -4.522566 -3.416703 -0.787946
C -4.031031 -2.108757 -0.677673
N -2.299057 0.456743 -0.314532
C -1.545744 1.532634 -0.256865
C -2.026527 2.914934 -0.285914
C -0.893743 3.743955 -0.243908
C 0.281883 2.884706 -0.172702
N -0.178432 1.589559 -0.187438
C -0.998708 5.128034 -0.270058
C -2.284333 5.667330 -0.340000
C -3.415142 4.841580 -0.377810
C -3.310007 3.448563 -0.352465
N 1.514016 3.317069 -0.104595
O -5.205791 1.069402 0.399824
O -9.081187 0.329591 0.883364
O -6.894699 -0.970414 -0.439279

H 3.757686 5.211892 -0.000041
H 6.245461 5.506110 0.170009
H 7.738416 3.551654 0.322197
H 6.807605 1.220033 0.310619
H 2.238902 -5.767528 0.077459
H 4.544719 -6.725748 0.313824
H 6.514470 -5.250668 0.474397
H 6.260461 -2.756767 0.405181
H -4.721955 -1.272166 -0.745615
H -5.594284 -3.555925 -0.934400
H -4.087821 -5.525767 -0.802390
H -1.616034 -5.214594 -0.472261
H -0.109118 5.756581 -0.237235
H -2.412348 6.750363 -0.362600
H -4.404791 5.297662 -0.425776
H -4.194666 2.807905 -0.354631
H 0.412653 0.764833 -0.138607
H -4.313319 0.732131 0.214643
H -5.796607 0.492411 -0.128488
H -9.358615 1.248537 0.785090
H -9.891096 -0.137969 1.123106
H 1.668431 -0.773086 -0.057447
H -6.787724 -1.118647 0.528259
H -7.783084 -0.583994 -0.460115
H -6.030476 0.150586 1.929355
O -6.655792 -0.526609 2.247428
H -7.516336 -0.097173 2.116694

C4e
E = -1972.783832
C 5.363708 -1.431605 -0.086045
C 4.177015 -0.705326 -0.045557
C 4.178067 0.698998 0.045721
C 5.365845 1.423495 0.086256
C 6.556092 0.693528 0.041539
C 6.555047 -0.703423 -0.041278
C 2.782226 -1.135672 -0.067313
N 2.014149 -0.001540 0.000040
C 2.783924 1.131445 0.067413
N 2.370495 -2.383151 -0.123053
C 1.082535 -2.747325 -0.062238
C 0.695388 -4.165185 -0.040658
C -0.701710 -4.164098 0.040525
C -1.086655 -2.745647 0.062262
N -0.001440 -1.928342 0.000027
C 1.422425 -5.347282 -0.072655
C 0.691887 -6.543100 -0.034100
C -0.701998 -6.542006 0.033652
C -1.430633 -5.345034 0.072361
N -2.374071 -2.379524 0.123124
C -2.783904 -1.131434 0.067377
C -4.178052 -0.699000 0.045624
C -4.177012 0.705319 -0.045691
C -2.782219 1.135679 -0.067404
N -2.014134 0.001547 0.000002
C -5.365813 -1.423521 0.086114
C -6.556071 -0.693571 0.041326
C -6.555040 0.703378 -0.041522
C -5.363709 1.431581 -0.086250
N -2.370492 2.383144 -0.123130
C -1.082518 2.747318 -0.062273
C -0.695399 4.165178 -0.040619
C 0.701699 4.164118 0.040587
C 1.086671 2.745671 0.062261
N 0.001452 1.928351 -0.000022
C -1.422476 5.347253 -0.072555
C -0.691981 6.543092 -0.033911
C 0.701903 6.542033 0.033870
C 1.430575 5.345083 0.072518
N 2.374080 2.379541 0.123120
O 4.439460 -4.411638 -0.700286
H 2.520682 5.350702 0.133010
H 1.238291 7.491519 0.058310

H -1.226926 7.493392 -0.058353
H -2.512573 5.354548 -0.133040
H -5.368453 -2.513375 0.160452
H -7.507023 -1.226906 0.072245
H -7.505205 1.238111 -0.072494
H -5.364756 2.521439 -0.160590
H -0.998274 0.000806 0.000032
H 2.512523 -5.354617 -0.133106
H 1.226803 -7.493415 -0.058590
H -1.238415 -7.491478 0.058020
H -2.520742 -5.350606 0.132848
H 5.368507 2.513351 0.160561
H 7.507050 1.226852 0.072493
H 7.505205 -1.238171 -0.072192
H 5.364748 -2.521466 -0.160350
H 0.998288 -0.000795 0.000064
H 3.728827 -3.748923 -0.633453
H 4.533736 -4.565368 -1.648142
H -3.734431 -3.743228 0.634242
O -4.446071 -4.404874 0.700871
H -4.540701 -4.558670 1.648681
H -3.728771 3.748888 -0.633792
O -4.439423 4.411582 -0.700614
H -4.533635 4.565413 -1.648461
H 3.734521 3.743320 0.633575
O 4.446152 4.404960 0.700404
H 4.540608 4.558607 1.648257

D0a
E = -1667.814768
N 0.032478 2.015306 0.000000
C -1.087247 2.798341 0.000000
C -0.628925 4.186267 0.000000
C 0.773280 4.165748 0.000000
C 1.189870 2.766394 0.000000
C 1.513738 5.343478 0.000000
C 0.803950 6.543026 0.000000
C -0.599979 6.562698 0.000000
C -1.339767 5.381491 0.000000
N -2.340919 2.425379 0.000000
C -2.713590 1.135411 -0.000000
C -4.142539 0.785705 -0.000000
C -4.186283 -0.608332 -0.000000
C -2.782296 -1.042578 -0.000000
N -1.929554 0.039385 -0.000000
C -5.298336 1.553030 -0.000000
C -6.516375 0.866373 -0.000000
C -6.560934 -0.533596 -0.000000
C -5.390214 -1.298191 -0.000000
N -2.461235 -2.321241 -0.000000
C -1.216500 -2.768146 -0.000000
C -0.819100 -4.153647 -0.000000
C 0.592832 -4.200797 -0.000000
C 1.031714 -2.832902 -0.000000
N -0.052403 -2.028765 -0.000000
C -1.579464 -5.327514 -0.000000
C -0.899928 -6.535333 -0.000000
C 0.510388 -6.583903 -0.000000
C 1.273092 -5.426960 -0.000000
N 2.300512 -2.381689 -0.000000
C 2.756226 -1.098569 0.000000
C 4.170342 -0.755783 0.000000
C 4.174213 0.647250 0.000000
C 2.773793 1.057027 0.000000
N 1.947215 -0.034675 0.000000
C 5.360928 -1.482542 0.000000
C 6.552824 -0.761459 0.000000
C 6.555241 0.642722 0.000000
C 5.365517 1.367131 0.000000
N 2.424911 2.348113 0.000000
H 5.356788 2.457024 0.000000
H 7.509189 1.171515 0.000000
H 7.502997 -1.296756 0.000000
H 5.385181 -2.574605 0.000000
H -2.668027 -5.277765 -0.000000
H -1.461073 -7.470481 -0.000000
H 1.008578 -7.553959 -0.000000
H 2.362988 -5.488609 -0.000000
H -0.029929 -1.010972 -0.000000
H -5.252327 2.642039 -0.000000
H -7.451208 1.428640 -0.000000
H -7.529964 -1.034799 -0.000000
H -5.417472 -2.387878 -0.000000
H 2.603234 5.319765 0.000000
H 1.348011 7.488525 0.000000
H -1.118309 7.522396 0.000000
H -2.429540 5.383021 0.000000
H 0.000000 1.001629 0.000000
H(Iso=2) 3.015013 -3.102479 -0.000000

D0b
E = -1667.812997
6 0.000000 4.833157 -2.847806
6 0.000000 3.498890 -2.460562
6 0.000000 2.463533 -3.406058
6 0.000000 2.733175 -4.771719
6 0.000000 4.072724 -5.163383
6 0.000000 5.105871 -4.216258
6 0.000000 2.843228 -1.152171
7 0.000000 1.469453 -1.300611
6 0.000000 1.236627 -2.600180
7 0.000000 0.000000 -3.167530
6 -0.000000 -1.236627 -2.600180
6 -0.000000 -2.463533 -3.406058
6 -0.000000 -3.498890 -2.460562
6 -0.000000 -2.843228 -1.152171
7 -0.000000 -1.469453 -1.300611
6 -0.000000 -2.733175 -4.771719
6 -0.000000 -4.072724 -5.163383
6 -0.000000 -5.105871 -4.216258
6 -0.000000 -4.833157 -2.847806
7 0.000000 -3.500216 -0.006440
6 0.000000 -2.870798 1.157937
6 0.000000 -3.482592 2.467017
6 0.000000 -2.437623 3.408704
6 0.000000 -1.181015 2.679142
7 0.000000 -1.505381 1.347582
6 0.000000 -4.820088 2.868434
6 0.000000 -5.075548 4.233883
6 0.000000 -4.029023 5.176871
6 0.000000 -2.697465 4.779754
7 0.000000 -0.000000 3.264819
6 0.000000 1.181015 2.679142
7 0.000000 1.505381 1.347582
6 0.000000 2.870798 1.157937
6 0.000000 3.482592 2.467017
6 0.000000 2.437623 3.408704
6 0.000000 4.820088 2.868434
6 0.000000 5.075548 4.233883
6 0.000000 4.029023 5.176871
6 0.000000 2.697465 4.779754
7 0.000000 3.500216 -0.006440
1 0.000000 5.630173 -2.104325
1 0.000000 6.141221 -4.559457
1 0.000000 4.320644 -6.225360
1 0.000000 1.945767 -5.528829
1 -0.000000 -5.630173 -2.104325
1 -0.000000 -6.141221 -4.559457
1 -0.000000 -4.320644 -6.225360
1 -0.000000 -1.945767 -5.528829
1 0.000000 -1.881190 5.501742
1 0.000000 -4.273333 6.239940
1 0.000000 -6.107407 4.587714
1 0.000000 -5.623845 2.132586
1 0.000000 5.623845 2.132586
1 0.000000 6.107407 4.587714

1 0.000000 4.273333 6.239940
1 0.000000 1.881190 5.501742
1 0.000000 0.926814 0.501124
1 -0.000000 -0.926814 0.501124
1(Iso=2) 0.000000 0.000000 -4.181939

D0c
E = -1667.806845
7 -0.025378 -0.001814 2.026746
6 -0.195278 -1.149297 2.794533
6 -0.389881 -0.689198 4.158817
6 -0.309039 0.715007 4.153874
6 -0.060096 1.131021 2.787650
6 -0.457657 1.457313 5.325903
6 -0.687896 0.752805 6.501195
6 -0.769857 -0.652624 6.506442
6 -0.625388 -1.393917 5.339062
7 -0.158224 -2.394412 2.405637
6 -0.060075 -2.795197 1.143882
6 0.291772 -4.109374 0.707103
6 0.291772 -4.109374 -0.707103
6 -0.060075 -2.795197 -1.143882
7 -0.240586 -2.022695 0.000000
6 0.631449 -5.265234 1.431295
6 0.945005 -6.399289 0.709185
6 0.945005 -6.399289 -0.709185
6 0.631449 -5.265234 -1.431295
7 -0.158224 -2.394412 -2.405637
6 -0.195278 -1.149297 -2.794533
6 -0.389881 -0.689198 -4.158817
6 -0.309039 0.715007 -4.153874
6 -0.060096 1.131021 -2.787650
7 -0.025378 -0.001814 -2.026746
6 -0.625388 -1.393917 -5.339062
6 -0.769857 -0.652624 -6.506442
6 -0.687896 0.752805 -6.501195
6 -0.457657 1.457313 -5.325903
7 0.109557 2.372244 -2.374962
6 0.231291 2.699790 -1.093231
6 0.398411 4.104947 -0.697169
6 0.398411 4.104947 0.697169
6 0.231291 2.699790 1.093231
7 0.170008 1.880720 -0.000000
6 0.519398 5.278774 -1.426668
6 0.651422 6.466226 -0.700349
6 0.651422 6.466226 0.700349
6 0.519398 5.278774 1.426668
7 0.109557 2.372244 2.374962
1 0.511957 5.269852 2.516560
1 0.754374 7.412953 1.232398
1 0.754374 7.412953 -1.232398
1 0.511957 5.269852 -2.516560
1 -0.689705 -2.481776 -5.337649
1 -0.950993 -1.168882 -7.450179
1 -0.808292 1.295073 -7.439768
1 -0.393098 2.544993 -5.308343
1 0.261339 0.074421 -1.056933
1 0.637735 -5.255297 2.520900
1 1.202276 -7.319249 1.235790
1 1.202276 -7.319249 -1.235790
1 0.637735 -5.255297 -2.520900
1 -0.393098 2.544993 5.308343
1 -0.808292 1.295073 7.439768
1 -0.950993 -1.168882 7.450179
1 -0.689705 -2.481776 5.337649
1 0.261339 0.074421 1.056933
1(Iso=2) -0.883504 -1.235653 0.000000

D0d
E = -1667.801777
C -4.725111 2.992552 0.000000
C -3.774780 1.984639 0.000000
C -4.133339 0.635766 0.000000
C -5.460023 0.236654 0.000000
C -6.429378 1.247282 0.000000
C -6.069044 2.598756 0.000000
C -2.302973 1.990786 0.000000
N -1.803674 0.716478 0.000000
C -2.862084 -0.116771 0.000000
N -2.883902 -1.451182 0.000000
C -1.823084 -2.228037 0.000000
C -1.976351 -3.698681 0.000000
C -0.683757 -4.234280 0.000000
C 0.178167 -3.048807 0.000000
N -0.497693 -1.895100 0.000000
C -3.096730 -4.514236 0.000000
C -2.885958 -5.896353 0.000000
C -1.593487 -6.432813 0.000000
C -0.465922 -5.606653 0.000000
N 1.520720 -3.079283 0.000000
C 2.362516 -2.001970 0.000000
C 3.782060 -1.981155 0.000000
C 4.139278 -0.608349 0.000000
C 2.937455 0.168760 0.000000
N 1.891354 -0.735435 0.000000
C 4.771532 -2.985471 0.000000
C 6.091135 -2.585677 0.000000
C 6.448073 -1.213034 0.000000
C 5.490297 -0.220205 0.000000
N 2.899497 1.498092 0.000000
C 1.862940 2.278084 0.000000
N 0.507636 1.956023 0.000000
C -0.268712 3.070069 0.000000
C 0.609687 4.222377 0.000000
C 1.926782 3.735043 0.000000
C 0.334959 5.590267 0.000000
C 1.421086 6.456933 0.000000
C 2.741334 5.969568 0.000000
C 3.014713 4.605506 0.000000
N -1.598282 3.110333 0.000000
H -4.435438 4.043407 0.000000
H -6.851434 3.358934 0.000000
H -7.486520 0.977975 0.000000
H -5.731708 -0.818916 0.000000
H 0.533601 -6.046097 0.000000
H -1.463290 -7.515543 0.000000
H -3.742074 -6.572128 0.000000
H -4.098111 -4.084118 0.000000
H 5.756386 0.836536 0.000000
H 7.505062 -0.944077 0.000000
H 6.880666 -3.337965 0.000000
H 4.517542 -4.047145 0.000000
H -0.692972 5.952244 0.000000
H 1.252091 7.534369 0.000000
H 3.568025 6.681504 0.000000
H 4.035695 4.224562 0.000000
H 0.000000 1.059311 0.000000
H 0.869597 -0.614423 0.000000
H(Iso=2) 1.965600 -3.991212 0.000000

D0e
E = -1667.797243
N 0.000667 1.978292 0.000000
C -1.130850 2.759950 -0.000000
C -0.699206 4.143877 -0.000000
C 0.703389 4.142936 0.000000
C 1.133240 2.758435 0.000000
C 1.430711 5.333119 0.000000
C 0.707600 6.520694 0.000000
C -0.700270 6.521631 -0.000000
C -1.424956 5.335018 -0.000000
N -2.406210 2.399698 -0.000000
C -2.795234 1.139390 -0.000000
C -4.212300 0.741908 -0.000000
C -4.208014 -0.658822 -0.000000
C -2.776391 -1.010183 -0.000000

N -1.977990 0.047031 -0.000000
C -5.396745 1.463329 -0.000000
C -6.591861 0.738882 -0.000000
C -6.590298 -0.660537 -0.000000
C -5.393716 -1.383360 -0.000000
N -2.343322 -2.289389 -0.000000
C -1.062914 -2.763630 -0.000000
C -0.708320 -4.176613 -0.000000
C 0.704026 -4.177574 0.000000
C 1.060456 -2.765071 0.000000
N -0.000679 -1.958216 -0.000000
C -1.424547 -5.372943 -0.000000
C -0.705167 -6.565589 -0.000000
C 0.697675 -6.566537 0.000000
C 1.418656 -5.374859 0.000000
N 2.341414 -2.292397 0.000000
C 2.775675 -1.013635 0.000000
C 4.207562 -0.663428 0.000000
C 4.212918 0.737309 0.000000
C 2.796165 1.135951 0.000000
N 1.978109 0.044196 0.000000
C 5.392697 -1.388897 0.000000
C 6.589835 -0.667001 0.000000
C 6.592479 0.732418 0.000000
C 5.397930 1.457800 0.000000
N 2.408203 2.396626 0.000000
H 5.389232 2.547783 0.000000
H 7.545524 1.262901 0.000000
H 7.538977 -1.204163 0.000000
H 5.416424 -2.480952 0.000000
H -2.516362 -5.396488 -0.000000
H -1.239808 -7.515982 -0.000000
H 1.231036 -7.517649 0.000000
H 2.510440 -5.399872 0.000000
H -5.387192 2.553305 -0.000000
H -7.544495 1.270104 -0.000000
H -7.539856 -1.196962 -0.000000
H -5.418286 -2.475396 -0.000000
H 2.520377 5.321010 0.000000
H 1.238319 7.473732 0.000000
H -1.229722 7.475374 -0.000000
H -2.514636 5.324349 -0.000000
H -0.000000 0.950002 0.000000
H 3.068498 -2.999789 0.000000
H(Iso=2) -3.071165 -2.995998 -0.000000

D0f
E = -1667.789234
C -2.908904 4.794730 0.000000
C -2.492997 3.472325 0.000000
C -3.403905 2.415617 0.000000
C -4.772055 2.635053 0.000000
C -5.203427 3.966706 0.000000
C -4.289220 5.026164 0.000000
C -1.169112 2.833678 0.000000
N -1.272208 1.472174 0.000000
C -2.594225 1.178078 0.000000
N -3.201146 0.000004 0.000000
C -2.594206 -1.178089 0.000000
C -3.403901 -2.415616 0.000000
C -2.493004 -3.472334 0.000000
C -1.169112 -2.833703 0.000000
N -1.272216 -1.472173 0.000000
C -4.772055 -2.635037 0.000000
C -5.203441 -3.966686 0.000000
C -4.289244 -5.026153 0.000000
C -2.908926 -4.794734 0.000000
N -0.039669 -3.526853 0.000000
C 1.126355 -2.897628 -0.000000
C 2.432621 -3.505696 -0.000000
C 3.384846 -2.463960 -0.000000
C 2.635026 -1.236697 -0.000000
N 1.309789 -1.533886 -0.000000
C 2.824341 -4.847553 -0.000000
C 4.183063 -5.121860 -0.000000
C 5.137281 -4.083110 -0.000000
C 4.757897 -2.749727 -0.000000
N 3.170145 0.000002 -0.000000
C 2.635027 1.236693 -0.000000
N 1.309789 1.533882 -0.000000
C 1.126350 2.897627 -0.000000
C 2.432627 3.505697 -0.000000
C 3.384850 2.463963 -0.000000
C 2.824342 4.847550 -0.000000
C 4.183068 5.121859 -0.000000
C 5.137283 4.083113 -0.000000
C 4.757898 2.749726 -0.000000
N -0.039659 3.526848 0.000000
H -2.190561 5.614619 0.000000
H -4.661974 6.051443 0.000000
H -6.272142 4.185750 0.000000
H -5.475270 1.802313 0.000000
H -2.190592 -5.614632 0.000000
H -4.662010 -6.051428 0.000000
H -6.272157 -4.185719 0.000000
H -5.475260 -1.802289 0.000000
H 5.519276 -1.966777 -0.000000
H 6.198043 -4.336475 -0.000000
H 4.526352 -6.156858 -0.000000
H 2.075410 -5.639193 -0.000000
H 2.075411 5.639190 -0.000000
H 4.526354 6.156858 -0.000000
H 6.198046 4.336476 -0.000000
H 5.519276 1.966777 -0.000000
H 0.425286 0.978392 -0.000000
H 0.425292 -0.978393 -0.000000
H(Iso=2) 4.186592 -0.000000 -0.000000

D0g
E = -1667.785920
N 0.066909 1.978194 0.000000
C 1.205458 2.717713 0.000000
C 0.863148 4.115568 0.000000
C -0.543291 4.167303 -0.000000
C -1.037210 2.812037 -0.000000
C -1.223793 5.388249 -0.000000
C -0.464122 6.547831 -0.000000
C 0.945321 6.498531 0.000000
C 1.626526 5.290773 0.000000
N 2.460093 2.233907 0.000000
C 2.884387 0.943855 0.000000
C 4.263123 0.499226 0.000000
C 4.163260 -0.910058 0.000000
C 2.731467 -1.178592 0.000000
N 2.000943 -0.067349 0.000000
C 5.508268 1.130140 0.000000
C 6.645011 0.327797 0.000000
C 6.546875 -1.072793 0.000000
C 5.308239 -1.707279 0.000000
N 2.197081 -2.439491 0.000000
C 0.890813 -2.795397 0.000000
C 0.449563 -4.208505 0.000000
C -0.945211 -4.113397 -0.000000
C -1.251815 -2.661232 -0.000000
N -0.091377 -1.924434 -0.000000
C 1.091185 -5.438003 0.000000
C 0.285051 -6.583312 -0.000000
C -1.108569 -6.486141 -0.000000
C -1.748018 -5.240306 -0.000000
N -2.481696 -2.224436 -0.000000
C -2.790138 -0.909078 -0.000000
C -4.208580 -0.492190 -0.000000
C -4.187697 0.904738 -0.000000
C -2.758709 1.256813 -0.000000
N -1.966374 0.141805 -0.000000
C -5.398676 -1.201200 -0.000000

C -6.585310 -0.457262 -0.000000
C -6.564243 0.941136 -0.000000
C -5.356259 1.649585 -0.000000
N -2.325563 2.502404 -0.000000
H -5.333415 2.739525 -0.000000
H -7.507985 1.488475 -0.000000
H -7.545203 -0.975757 -0.000000
H -5.404712 -2.291263 -0.000000
H 2.178855 -5.537764 0.000000
H 0.753837 -7.568036 0.000000
H -1.706029 -7.398764 -0.000000
H -2.834414 -5.151563 -0.000000
H 5.608332 2.217460 0.000000
H 7.631162 0.793220 0.000000
H 7.458144 -1.671568 0.000000
H 5.256448 -2.798084 0.000000
H -2.313279 5.409735 -0.000000
H -0.959825 7.519230 -0.000000
H 1.509984 7.431526 0.000000
H 2.718477 5.279813 0.000000
H 0.000000 0.947894 0.000000
H 2.866631 -3.200433 0.000000
H(Iso=2) 3.186553 2.942872 0.000000

D0h
E = -1667.778049
N 0.000008 1.961657 0.157041
C -1.109136 2.747833 0.236784
C -0.713155 4.090992 0.388242
C 0.713176 4.090990 0.388243
C 1.109153 2.747831 0.236786
C 1.427226 5.307099 0.517566
C 0.711959 6.473053 0.639106
C -0.711933 6.473055 0.639105
C -1.427203 5.307102 0.517564
N -2.402120 2.289464 0.110570
C -2.821638 1.032296 -0.037376
C -4.219327 0.645956 -0.302654
C -4.153430 -0.746292 -0.412767
C -2.731467 -1.109731 -0.199591
N -1.979436 0.003491 0.021312
C -5.417938 1.328770 -0.447898
C -6.562559 0.565187 -0.707241
C -6.495608 -0.826417 -0.815205
C -5.281119 -1.507130 -0.667321
N -2.354993 -2.370490 -0.166489
C -1.076625 -2.732526 -0.091286
C -0.697585 -4.106814 0.316385
C 0.697565 -4.106814 0.316382
C 1.076605 -2.732527 -0.091292
N -0.000009 -1.968895 -0.361578
C -1.425756 -5.228577 0.665187
C -0.697778 -6.377175 1.014947
C 0.697758 -6.377175 1.014944
C 1.425735 -5.228578 0.665182
N 2.354975 -2.370495 -0.166500
C 2.731458 -1.109739 -0.199596
C 4.153424 -0.746309 -0.412770
C 4.219332 0.645938 -0.302652
C 2.821645 1.032286 -0.037374
N 1.979435 0.003490 0.021311
C 5.281108 -1.507155 -0.667325
C 6.495602 -0.826450 -0.815207
C 6.562563 0.565153 -0.707238
C 5.417948 1.328743 -0.447893
N 2.402135 2.289458 0.110574
H 5.492640 2.415145 -0.367441
H 7.523890 1.066107 -0.826751
H 7.407713 -1.389330 -1.017492
H 5.216219 -2.592304 -0.747405
H -2.515882 -5.219797 0.665276
H -1.231474 -7.287438 1.291897
H 1.231454 -7.287439 1.291892
H 2.515861 -5.219800 0.665267
H -5.492622 2.415173 -0.367449
H -7.523881 1.066149 -0.826757
H -7.407723 -1.389290 -1.017489
H -5.216238 -2.592279 -0.747397
H 2.518864 5.331071 0.529328
H 1.239543 7.421702 0.742637
H -1.239515 7.421704 0.742635
H -2.518840 5.331077 0.529325
H 0.000007 0.934125 0.067986
H(Iso=2) -3.107499 3.017212 0.072914
H 3.107519 3.017201 0.072920

D0i
E = -1667.766641
N -0.000400 -1.981887 -0.000188
C -1.142028 -2.752139 -0.000080
C -0.700728 -4.149234 -0.000033
C 0.699327 -4.149424 0.000055
C 1.141019 -2.752452 0.000065
C 1.425496 -5.333525 0.000133
C 0.700140 -6.525820 0.000139
C -0.702192 -6.525629 0.000050
C -1.427223 -5.333136 -0.000049
N -2.383163 -2.368240 -0.000199
C -2.751417 -1.068118 -0.000268
C -4.169317 -0.702370 -0.000220
C -4.199369 0.696219 -0.000246
C -2.800297 1.107377 -0.000180
N -1.955013 0.018846 0.000043
C -5.336449 -1.456325 -0.000228
C -6.545827 -0.758369 -0.000302
C -6.576527 0.643855 -0.000343
C -5.400793 1.396594 -0.000312
N -2.430294 2.385328 -0.000175
C -1.201915 2.805219 -0.000070
C -0.709021 4.135706 -0.000024
C 0.710422 4.135514 0.000060
C 1.202944 2.804892 0.000077
N 0.000396 1.918199 0.000108
C -1.429455 5.345879 -0.000073
C -0.709028 6.522675 -0.000026
C 0.711079 6.522481 0.000060
C 1.431187 5.345490 0.000101
N 2.431192 2.384641 0.000128
C 2.800727 1.106564 0.000173
C 4.199638 0.694872 0.000191
C 4.169039 -0.703704 0.000203
C 2.750996 -1.068914 0.000116
N 1.955016 0.018363 0.000184
C 5.401336 1.394779 0.000207
C 6.576778 0.641584 0.000254
C 6.545531 -0.760627 0.000277
C 5.335879 -1.458112 0.000253
N 2.382272 -2.368910 0.000117
H 5.301156 -2.547558 0.000251
H 7.485863 -1.313528 0.000304
H 7.541186 1.151539 0.000276
H 5.422010 2.484707 0.000200
H -2.518832 5.334800 -0.000148
H -1.236682 7.477228 -0.000066
H 1.238994 7.476891 0.000085
H 2.520561 5.334117 0.000160
H 0.000250 1.264689 0.797744
H -5.302152 -2.545784 -0.000230
H -7.486376 -1.310902 -0.000342
H -7.540735 1.154187 -0.000397
H -5.421044 2.486529 -0.000331
H 2.515210 -5.321914 0.000208
H 1.232120 -7.478068 0.000216
H -1.234433 -7.477731 0.000060
H -2.516934 -5.321223 -0.000112
H -0.000243 -0.967651 -0.000398

H(Iso=2) 0.000349 1.264481 -0.797359

D1a
E = -1744.220132
C 4.934092 -2.213815 0.000000
C 3.868699 -1.318396 0.000000
C 4.071569 0.069490 0.000000
C 5.362555 0.600713 0.000000
C 6.429266 -0.295224 0.000000
C 6.221081 -1.683271 0.000000
C 2.426440 -1.521131 0.000000
N 1.766034 -0.324573 0.000000
C 2.719405 0.620747 0.000000
N 2.444610 1.955272 0.000000
C 1.238018 2.554524 0.000000
C 0.945580 3.966742 0.000000
C -0.463009 4.069059 0.000000
C -1.009265 2.737127 0.000000
N 0.066840 1.875635 0.000000
C 1.742173 5.121619 0.000000
C 1.097354 6.348715 0.000000
C -0.309414 6.446179 0.000000
C -1.104064 5.311574 0.000000
N -2.299627 2.450213 0.000000
C -2.793050 1.228814 0.000000
C -4.243154 0.992205 0.000000
C -4.392004 -0.394793 0.000000
C -3.024981 -0.936196 0.000000
N -2.097769 0.041387 0.000000
C -5.341329 1.840747 0.000000
C -6.605832 1.244917 0.000000
C -6.754584 -0.148154 0.000000
C -5.642868 -0.995431 0.000000
N -2.826753 -2.264956 0.000000
C -1.628229 -2.787932 0.000000
N -0.423758 -2.141240 0.000000
C 0.635861 -3.022393 0.000000
C 0.057688 -4.363486 0.000000
C -1.337146 -4.219619 0.000000
C 0.654906 -5.620057 0.000000
C -0.190013 -6.728224 0.000000
C -1.586781 -6.583228 0.000000
C -2.182886 -5.323734 0.000000
N 1.911077 -2.756075 0.000000
H 4.753976 -3.288774 0.000000
H 7.082725 -2.352081 0.000000
H 7.450051 0.089568 0.000000
H 5.537356 1.675442 0.000000
H -2.192398 5.366504 0.000000
H -0.774211 7.432831 0.000000
H 1.691561 7.263458 0.000000
H 2.829148 5.058106 0.000000
H 0.000000 0.859992 0.000000
H -5.747056 -2.080465 0.000000
H -7.757867 -0.576518 0.000000
H -7.496632 1.874813 0.000000
H -5.218387 2.923853 0.000000
H 1.739744 -5.723700 0.000000
H 0.239668 -7.730897 0.000000
H -2.214100 -7.475540 0.000000
H -3.265379 -5.197668 0.000000
H -0.349419 -1.129110 0.000000
H(Iso=2) 3.255954 2.589445 0.000000
O 4.688428 3.794874 0.000000
H 5.183394 4.111792 0.766179
H 5.183394 4.111792 -0.766179

D1b
E = -1744.207801
C -4.674662 2.680298 -0.025963
C -3.316501 2.368035 -0.008815
C -2.339098 3.376220 -0.027185
C -2.679415 4.723605 -0.058411
C -4.037073 5.038119 -0.073023
C -5.014837 4.032889 -0.057867
C -2.607028 1.085474 0.011300
N -1.271763 1.369581 0.003059
C -1.033024 2.730038 -0.016296
N -3.169062 -0.101661 0.024844
C -2.463544 -1.251121 0.002041
C -3.151742 -2.556140 -0.042910
C -2.128237 -3.506312 -0.051703
C -0.876779 -2.743704 -0.015322
N -1.124502 -1.386958 0.011717
C -4.488822 -2.934439 -0.090187
C -4.758582 -4.306613 -0.139717
C -3.731211 -5.256420 -0.143134
C -2.389280 -4.867462 -0.099988
N 0.289779 -3.355465 -0.010742
C 1.458266 -2.737762 0.006199
C 2.747094 -3.382913 0.020705
C 3.737922 -2.375721 0.030705
C 3.027575 -1.128076 0.020744
N 1.701297 -1.380751 0.008094
C 3.095711 -4.737160 0.025003
C 4.444568 -5.055166 0.039618
C 5.435132 -4.050388 0.049899
C 5.100553 -2.705597 0.045581
N 3.547875 0.113751 0.020197
C 2.897936 1.309934 0.008190
C 3.584141 2.592226 -0.000035
C 2.539951 3.529477 -0.014294
C 1.301992 2.757090 -0.012565
N 1.564339 1.414611 0.001076
C 4.919131 2.996167 0.002437
C 5.174927 4.365586 -0.009583
C 4.129157 5.302334 -0.023837
C 2.796229 4.897268 -0.026385
N 0.108268 3.358794 -0.022495
O -6.083742 -0.156565 0.357326
H 1.977567 5.616729 -0.037609
H 4.369844 6.366091 -0.033167
H 6.207066 4.717782 -0.008038
H 5.749875 2.287042 0.013237
H 2.321976 -5.504431 0.016819
H 4.752534 -6.101344 0.043223
H 6.486239 -4.340614 0.061421
H 5.883914 -1.945443 0.053829
H 0.977947 -0.664652 -0.001810
H -5.298163 -2.201777 -0.087720
H -5.795651 -4.642648 -0.178823
H -3.983849 -6.316968 -0.183191
H -1.578853 -5.596360 -0.106992
H -1.908239 5.493407 -0.073211
H -4.347008 6.083720 -0.098789
H -6.068422 4.314707 -0.073028
H -5.440126 1.901132 -0.011591
H -0.561134 0.645201 0.017810
H -5.110058 -0.162660 0.295299
H -6.253551 -0.218622 1.305099
H(Iso=2) 4.561714 0.160313 0.027162

D1c
E = -1744.206389
C 0.872976 -5.431643 -0.460882
C 0.285264 -4.188153 -0.191887
C -1.117228 -4.032860 -0.191309
C -1.962691 -5.114358 -0.454593
C -1.375300 -6.345638 -0.702490
C 0.026490 -6.501711 -0.706588
C 0.820919 -2.883475 0.086403
N -0.193649 -2.007331 0.239594
C -1.407025 -2.644305 0.074445
N 2.125136 -2.543633 0.136968
C 2.688205 -1.302418 0.113838
C 4.125656 -1.067488 0.126923

C 4.238190 0.329449 0.025419
C 2.870778 0.838224 -0.031801
N 1.968372 -0.183492 0.032155
C 5.256601 -1.879483 0.205338
C 6.501095 -1.253291 0.175872
C 6.612075 0.142073 0.071186
C 5.481648 0.953155 -0.005693
N 2.596752 2.142999 -0.136678
C 1.386440 2.630760 -0.183181
C 1.075602 4.053038 -0.275256
C -0.319525 4.181148 -0.267707
C -0.884355 2.837602 -0.172576
N 0.173181 1.964186 -0.132528
C 1.904410 5.168519 -0.354208
C 1.289005 6.416128 -0.428024
C -0.109931 6.543754 -0.420533
C -0.937167 5.425852 -0.338908
N -2.167941 2.585587 -0.119984
C -2.654189 1.336725 -0.021020
C -4.104075 1.091268 0.038879
C -4.241283 -0.296502 0.122420
C -2.869115 -0.826199 0.105041
N -1.950683 0.194764 0.033164
C -5.206281 1.933369 0.018096
C -6.467281 1.333325 0.089431
C -6.605529 -0.057739 0.173853
C -5.489073 -0.899536 0.189672
N -2.622279 -2.120517 0.104784
O -0.010423 -0.135619 2.298060
H -5.590124 -1.983172 0.251882
H -7.605499 -0.490741 0.226660
H -7.361776 1.957674 0.078043
H -5.088659 3.014847 -0.051542
H 2.988778 5.060172 -0.359145
H 1.903643 7.314994 -0.493391
H -0.553496 7.538448 -0.480295
H -2.023650 5.510652 -0.331888
H 0.062305 0.951592 -0.096108
H 5.195361 -2.966551 0.292486
H 7.406758 -1.857860 0.236537
H 7.603846 0.595551 0.050738
H 5.558644 2.037355 -0.087723
H -3.044025 -4.979405 -0.458699
H -2.004620 -7.213460 -0.903137
H 0.451372 -7.484893 -0.911611
H 1.955293 -5.573019 -0.479968
H -0.088378 -1.084175 0.690042
H -0.798220 0.417423 2.370011
H 0.726625 0.476237 2.409543
H(Iso=2) 2.775147 -3.322201 0.099798

D2a
E = -1820.620322
C 4.930566 3.343676 0.063210
C 3.629687 2.851562 0.036607
C 2.521034 3.709630 0.010587
C 2.677002 5.092364 0.010066
C 3.978812 5.588010 0.036971
C 5.088771 4.727811 0.063163
C 3.121580 1.482056 0.028708
N 1.757371 1.568374 -0.001174
C 1.322436 2.875748 -0.014285
N 3.862704 0.404842 0.047237
C 3.328129 -0.827521 0.037108
C 4.199817 -2.010841 0.061846
C 3.338513 -3.108188 0.045244
C 1.984127 -2.540276 0.010563
N 2.023920 -1.165808 0.006254
C 5.578691 -2.164813 0.095451
C 6.072059 -3.472200 0.111900
C 5.207584 -4.574862 0.095240
C 3.820212 -4.409945 0.061505
N 0.918072 -3.315474 -0.010729
C -0.327441 -2.877402 -0.045016
C -1.494922 -3.720829 -0.060733
C -2.637092 -2.891583 -0.098297
C -2.139696 -1.535331 -0.106687
N -0.786599 -1.577545 -0.072763
C -1.604816 -5.113987 -0.040234
C -2.878743 -5.658861 -0.056651
C -4.021481 -4.834318 -0.091186
C -3.923540 -3.450961 -0.111818
N -2.847748 -0.392110 -0.138063
C -2.373559 0.884506 -0.123599
C -3.227846 2.070053 -0.140696
C -2.320752 3.139693 -0.106699
C -0.990717 2.547647 -0.074840
N -1.064482 1.183797 -0.084029
C -4.603659 2.307042 -0.171161
C -5.032261 3.632202 -0.166211
C -4.120250 4.698775 -0.133893
C -2.748594 4.464344 -0.104034
N 0.099431 3.323393 -0.044116
O -5.603975 -0.809003 -0.376838
H -2.025033 5.279154 -0.078292
H -4.494976 5.723242 -0.132762
H -6.102475 3.842958 -0.189181
H -5.328610 1.497656 -0.199089
H -0.709234 -5.734156 -0.011443
H -3.004069 -6.742278 -0.041546
H -5.008981 -5.297628 -0.101281
H -4.812680 -2.822059 -0.138163
H -0.195551 -0.748974 -0.072555
H 6.241946 -1.299888 0.108178
H 7.149572 -3.640787 0.138134
H 5.628955 -5.581133 0.108940
H 3.141910 -5.263161 0.048365
H 1.810218 5.752569 -0.010783
H 4.142897 6.666490 0.037586
H 6.092247 5.154870 0.083663
H 5.783337 2.665386 0.083037
H 1.161587 0.746558 -0.009167
H(Iso=2) -3.880808 -0.504396 -0.193837
H -6.274350 -0.400487 0.216507
H -5.975149 -0.715346 -1.262928
O -7.365106 0.619323 1.105813
H -7.130655 0.920590 1.992198
H -8.318038 0.472792 1.141504

D2b
E = -1820.613500
C 2.608538 4.847578 -0.026016
C 2.364182 3.477620 -0.015539
C 3.409491 2.542250 -0.005027
C 4.741156 2.960779 -0.004968
C 4.986091 4.332187 -0.015460
C 3.936887 5.264406 -0.025875
C 1.127284 2.708304 -0.012569
N 1.387081 1.367218 -0.001736
C 2.724588 1.253116 0.002802
N 3.366473 0.051109 0.012731
C 2.813563 -1.176960 0.014142
C 3.479907 -2.455525 0.024512
C 2.452915 -3.424721 0.016487
C 1.187459 -2.737549 0.002653
N 1.476985 -1.389836 0.002976
C 4.827211 -2.845602 0.039002
C 5.103768 -4.204060 0.044710
C 4.075380 -5.168757 0.036272
C 2.742060 -4.792529 0.022142
N 0.008170 -3.332824 -0.011466
C -1.153262 -2.712829 -0.015438
C -2.410853 -3.466055 -0.046364
C -3.426959 -2.507770 -0.039253
C -2.727797 -1.209238 -0.000872
N -1.390110 -1.356311 0.007777

C -2.684010 -4.825466 -0.087291
C -4.028779 -5.203482 -0.124226
C -5.048809 -4.245150 -0.121819
C -4.767564 -2.875595 -0.079605
N -3.418642 -0.050751 0.018454
C -2.830339 1.123492 0.008928
C -3.506796 2.423027 -0.006618
C -2.503397 3.405012 -0.021136
C -1.214043 2.724670 -0.012979
N -1.487973 1.372069 0.002728
C -2.809529 4.761016 -0.046172
C -4.158322 5.110370 -0.058117
C -5.162220 4.130644 -0.046297
C -4.856798 2.770156 -0.020491
N -0.059038 3.325410 -0.019095
O 6.258200 0.061515 0.029655
H 1.781954 5.557982 -0.034237
H 4.169990 6.329924 -0.034094
H 6.016359 4.690779 -0.015739
H 5.563773 2.247285 0.002995
H 1.934113 -5.523728 0.015422
H 4.339014 -6.227062 0.040943
H 6.142394 -4.537092 0.055953
H 5.628650 -2.108461 0.045318
H 0.792030 -0.636517 -0.007248
H -5.570553 -2.135982 -0.079223
H -6.088679 -4.573077 -0.155766
H -4.290338 -6.262118 -0.158371
H -1.879640 -5.561088 -0.093050
H -2.018780 5.510774 -0.058046
H -4.441218 6.163758 -0.079018
H -6.208134 4.439875 -0.059178
H -5.641527 2.010394 -0.010248
H -0.804902 0.620300 0.014345
H(Iso=2) 4.396195 0.078635 0.018642
H 6.845752 0.121727 -0.734331
H 6.837984 0.139523 0.797936
H -5.359344 -0.078776 0.226659
O -6.331904 -0.056244 0.305324
H -6.482076 -0.111827 1.256818

D2c
E = -1820.606241
C 4.833789 2.561142 0.237918
C 3.835074 1.619485 0.034328
C 4.126521 0.256689 -0.070382
C 5.427068 -0.219321 0.019772
C 6.439219 0.724072 0.226006
C 6.147064 2.089462 0.333034
C 2.371820 1.714036 -0.107272
N 1.816053 0.474704 -0.292044
C 2.822317 -0.404248 -0.269592
N 2.742732 -1.741164 -0.381603
C 1.594940 -2.375814 -0.423600
C 1.471700 -3.823398 -0.581475
C 0.108334 -4.137960 -0.511587
C -0.618014 -2.888299 -0.310702
N 0.327818 -1.879710 -0.292050
C 2.437248 -4.807369 -0.769080
C 1.993295 -6.122941 -0.884016
C 0.626571 -6.439046 -0.812901
C -0.337939 -5.451216 -0.625603
N -1.913879 -2.805990 -0.163770
C -2.570527 -1.653544 -0.002635
C -4.020054 -1.596446 0.177360
C -4.341888 -0.232426 0.280654
C -3.053814 0.437613 0.145611
N -2.032546 -0.400349 -0.005781
C -5.004266 -2.576863 0.247254
C -6.321385 -2.157823 0.428085
C -6.643514 -0.796314 0.535347
C -5.658458 0.187468 0.463409
N -2.899672 1.792227 0.129128
C -1.764705 2.511185 -0.001397
C -1.676178 3.907783 -0.332897
C -0.297507 4.201783 -0.385183
C 0.421091 2.983104 -0.103210
N -0.522152 2.001487 0.125573
C 0.155015 5.485660 -0.702662
C -0.798210 6.461194 -0.952177
C -2.176967 6.167654 -0.905344
C -2.634245 4.893838 -0.604545
N 1.739963 2.869334 -0.082120
O 0.132125 0.098414 2.011555
O 2.660222 -0.789208 2.712672
H 4.600214 3.623048 0.318023
H 6.962186 2.797229 0.490872
H 7.476187 0.393469 0.300794
H 5.646641 -1.283473 -0.073170
H -1.400498 -5.687004 -0.570481
H 0.316999 -7.480757 -0.907845
H 2.717659 -6.924692 -1.033319
H 3.494644 -4.549071 -0.825344
H 0.122790 -0.896562 -0.112305
H -5.932044 1.240940 0.555129
H -7.684137 -0.503271 0.679159
H -7.119697 -2.898612 0.488756
H -4.745319 -3.632312 0.162527
H 1.222981 5.698156 -0.746497
H -0.481431 7.476231 -1.194685
H -2.896619 6.959728 -1.115110
H -3.705837 4.685991 -0.586670
H -0.302059 1.102466 0.594152
H 1.033710 -0.191761 2.263409
H -0.459230 -0.366506 2.612174
H 3.013633 -1.526770 2.198416
H 3.369037 -0.133629 2.685866
H(Iso=2) -3.757180 2.334910 0.125881

D2d
E = -1820.600392
C 0.397112 5.838681 0.038758
C -0.091470 4.532591 0.019351
C -1.469485 4.271272 -0.016093
C -2.401562 5.304451 -0.032906
C -1.915113 6.609788 -0.013471
C -0.536461 6.872787 0.021959
C 0.511401 3.208642 0.024913
N -0.378877 2.212131 -0.001451
C -1.605318 2.818788 -0.026391
N -2.805783 2.231971 -0.054627
C -2.987126 0.941740 -0.045103
C -4.284660 0.281392 -0.076110
C -4.060595 -1.104513 -0.037947
C -2.608635 -1.301744 0.011035
N -2.038597 -0.062512 0.000653
C -5.567130 0.813674 -0.139794
C -6.630308 -0.087040 -0.166986
C -6.409231 -1.471377 -0.131703
C -5.122555 -2.006807 -0.067360
N -1.995350 -2.463233 0.047383
C -0.654660 -2.598306 0.026772
C -0.042046 -3.938313 0.012713
C 1.338098 -3.715840 -0.009292
C 1.496117 -2.253007 -0.016201
N 0.272001 -1.620156 0.008972
C -0.573403 -5.222251 0.011482
C 0.335372 -6.285866 0.001124
C 1.714953 -6.063692 -0.006064
C 2.244708 -4.768927 -0.011646
N 2.689442 -1.685041 -0.039025
C 2.908236 -0.375119 -0.009982
C 4.195799 0.280649 0.033790
C 3.960414 1.675272 0.069895
C 2.536996 1.840441 0.041712
N 1.957052 0.621507 -0.001304

C 5.018773 2.591800 0.128368
C 6.307096 2.082817 0.151316
C 6.543265 0.693704 0.116649
C 5.503892 -0.222874 0.058205
N 1.855449 3.001224 0.050286
O 5.106523 -3.314120 -0.470559
O -3.731133 -4.799254 0.482918
H 1.464501 6.068917 0.066228
H -0.190634 7.906965 0.036520
H -2.614602 7.446535 -0.025975
H -3.469858 5.090195 -0.060467
H -4.956891 -3.085858 -0.032353
H -7.264424 -2.147906 -0.156390
H -7.653038 0.288988 -0.218186
H -5.722820 1.891809 -0.169548
H -1.035719 0.085801 0.036563
H 3.323363 -4.603018 -0.020306
H 2.394987 -6.916716 -0.008000
H -0.042771 -7.309078 0.000301
H -1.649689 -5.403217 0.023741
H 4.853473 3.670538 0.158443
H 7.154381 2.767759 0.198211
H 7.571730 0.331425 0.137284
H 5.693657 -1.298269 0.022269
H 0.954284 0.448379 -0.030567
H 4.276131 -2.807198 -0.408887
H 5.214026 -3.471956 -1.416322
H -3.131197 -4.032864 0.419130
H -3.804793 -4.959578 1.431464
H(Iso=2) 2.430581 3.837227 0.075815

D2e
E = -1820.599183
C -4.346407 2.796758 -0.386168
C -2.997718 2.460452 -0.288822
C -1.998966 3.446693 -0.250626
C -2.309229 4.800513 -0.304452
C -3.657525 5.141370 -0.400511
C -4.655174 4.156443 -0.440998
C -0.710411 2.770182 -0.161770
N -0.980741 1.416117 -0.155184
C -2.320692 1.162524 -0.226865
N -2.912898 -0.007425 -0.249849
C -2.242520 -1.175412 -0.234555
C -2.969918 -2.456042 -0.321011
C -1.980338 -3.440263 -0.279975
C -0.707363 -2.719290 -0.172488
N -0.910839 -1.355384 -0.152803
C -4.315760 -2.783139 -0.440232
C -4.629487 -4.145219 -0.512128
C -3.637277 -5.130909 -0.466661
C -2.286176 -4.791021 -0.349463
N 0.437905 -3.366927 -0.107453
C 1.619980 -2.779290 -0.023895
C 2.889187 -3.456307 0.060756
C 3.902424 -2.473894 0.129913
C 3.225417 -1.208863 0.084276
N 1.895816 -1.429193 -0.004157
C 3.203966 -4.818922 0.080221
C 4.541400 -5.170255 0.169620
C 5.554359 -4.190134 0.239087
C 5.253663 -2.837709 0.220532
N 3.777719 0.018810 0.118430
C 3.165650 1.233588 0.076759
C 3.890176 2.494181 0.113492
C 2.877763 3.463362 0.045521
C 1.618443 2.729901 -0.026091
N 1.838126 1.380232 -0.004196
C 5.234741 2.856882 0.192409
C 5.532866 4.217638 0.201296
C 4.518729 5.186378 0.133179
C 3.176373 4.822657 0.054281
N 0.445638 3.368735 -0.101974
O -5.877066 0.022127 -0.063826
O -6.552463 -0.131754 2.679829
H 2.381876 5.566876 0.000633
H 4.792159 6.242218 0.142275
H 6.573639 4.537727 0.262047
H 6.041365 2.122263 0.246051
H 2.413116 -5.566630 0.025840
H 4.822945 -6.223719 0.186912
H 6.595694 -4.506300 0.308715
H 6.053736 -2.097091 0.275486
H -5.095166 -2.017614 -0.472895
H -5.673865 -4.445264 -0.609342
H -3.924654 -6.181686 -0.527001
H -1.503343 -5.548852 -0.317370
H -1.523678 5.555239 -0.275084
H -3.943881 6.193024 -0.447256
H -5.699721 4.460724 -0.519616
H -5.124775 2.028630 -0.411972
H -0.285207 0.679834 -0.097317
H -4.906093 -0.009895 -0.041214
H -6.127029 -0.024266 0.878173
H -7.028716 -0.943862 2.890810
H -7.156137 0.567328 2.958619
H 1.187373 -0.699951 -0.054173
H(Iso=2) 4.790464 0.036392 0.182174

D3a
E = -1897.018594
C 4.412536 4.107403 -0.076932
C 3.818104 2.853132 -0.050866
C 4.579867 1.684957 -0.006904
C 5.967227 1.717900 0.013229
C 6.573653 2.976601 -0.013982
C 5.809240 4.150241 -0.058293
C 2.419251 2.406096 -0.056348
N 2.337663 1.034491 -0.023667
C 3.607691 0.582803 0.011475
N 4.031924 -0.690384 0.062884
C 3.202478 -1.701475 0.073111
C 3.594972 -3.107780 0.119907
C 2.419426 -3.872091 0.100555
C 1.294413 -2.943098 0.039496
N 1.836097 -1.675753 0.034933
C 4.850541 -3.704997 0.169898
C 4.893821 -5.096963 0.202215
C 3.716788 -5.863253 0.182619
C 2.460832 -5.262758 0.130216
N 0.039285 -3.287536 -0.018086
C -0.976866 -2.419332 -0.097524
C -2.350158 -2.888554 -0.222644
C -3.151351 -1.739746 -0.302368
C -2.201493 -0.637124 -0.185355
N -0.928946 -1.054731 -0.086435
C -2.895797 -4.168307 -0.298969
C -4.272412 -4.274783 -0.471802
C -5.074781 -3.126440 -0.584666
C -4.529545 -1.846352 -0.508064
N -2.568026 0.673645 -0.154396
C -1.767410 1.748596 -0.100782
C -2.154608 3.139917 -0.041075
C -0.950717 3.876610 -0.041670
C 0.146856 2.942090 -0.081814
N -0.415148 1.683127 -0.117312
C -0.951600 5.272839 0.002067
C -2.180691 5.913005 0.047585
C -3.383702 5.179532 0.057596
C -3.393950 3.792505 0.015663
N 1.423932 3.273170 -0.074981
O -5.205016 1.230706 0.312306
O -7.314060 -0.115601 -0.699129
O -6.436659 -0.788144 1.873023
H 3.811664 5.016305 -0.110612
H 6.317570 5.115418 -0.078337

H 7.662022 3.050304 -0.000416
H 6.552028 0.798677 0.048404
H 1.542745 -5.849676 0.112623
H 3.791229 -6.951317 0.208170
H 5.858379 -5.604714 0.242637
H 5.756255 -3.098990 0.182701
H -5.173405 -0.978834 -0.628243
H -6.146954 -3.231862 -0.760818
H -4.735028 -5.260274 -0.542121
H -2.254206 -5.047242 -0.234801
H -0.011170 5.823361 0.000511
H -2.221452 7.002505 0.081101
H -4.331831 5.716907 0.103287
H -4.326930 3.230474 0.047755
H 0.113479 0.814242 -0.145548
H(Iso=2) -3.597168 0.869837 -0.096650
H -5.953395 1.012316 -0.284119
H 1.306223 -0.810394 0.013247
H -7.439052 -0.440955 0.208906
H -8.194373 0.081476 -1.036442
H -5.465125 0.741860 1.114051
H -6.791426 -0.859139 2.767945
H -5.917328 -1.595238 1.743148

D3b
E = -1897.005538
C -4.910170 2.429375 -0.107159
C -3.548471 2.142950 -0.042581
C -2.581709 3.163444 -0.033548
C -2.948599 4.506228 -0.091773
C -4.312271 4.791928 -0.154024
C -5.274774 3.774227 -0.161497
C -1.268043 2.519682 0.009317
N -1.498050 1.159712 0.015695
C -2.833697 0.867403 -0.004115
N -3.394364 -0.318645 0.002056
C -2.684946 -1.466436 -0.006638
C -3.364805 -2.775579 -0.050657
C -2.334238 -3.718084 -0.041481
C -1.089775 -2.945461 0.000916
N -1.346693 -1.591377 0.017638
C -4.698555 -3.165272 -0.110226
C -4.956936 -4.539488 -0.152843
C -3.922191 -5.481571 -0.138078
C -2.584311 -5.081787 -0.083187
N 0.080833 -3.546629 0.017141
C 1.246445 -2.925375 0.028975
C 2.526590 -3.582312 0.055653
C 3.530148 -2.589137 0.049078
C 2.833217 -1.326417 0.017323
N 1.502378 -1.571013 0.009599
C 2.846028 -4.943008 0.084185
C 4.187374 -5.288734 0.107144
C 5.192933 -4.300493 0.102143
C 4.885816 -2.948815 0.073503
N 3.356959 -0.086552 -0.006117
C 2.696143 1.105631 -0.017953
C 3.369079 2.396448 -0.065315
C 2.321620 3.332134 -0.050243
C 1.088313 2.548950 0.003354
N 1.357258 1.210461 0.015600
C 4.701405 2.805721 -0.129913
C 4.955031 4.173916 -0.180498
C 3.908933 5.106935 -0.168606
C 2.577325 4.702599 -0.104608
N -0.118725 3.141129 0.024803
O 6.265618 -0.051428 -0.042804
O -6.314709 -0.407089 0.290821
H 1.768146 5.435200 -0.088705
H 4.141707 6.171656 -0.212677
H 5.986078 4.526418 -0.233114
H 5.518541 2.086562 -0.143191
H 2.054570 -5.692021 0.088046
H 4.475107 -6.340500 0.129704
H 6.238948 -4.609138 0.121492
H 5.670838 -2.194568 0.068994
H 0.792233 -0.842178 -0.014695
H -5.513767 -2.439340 -0.122276
H -5.990918 -4.883824 -0.201160
H -4.166305 -6.544318 -0.173283
H -1.767993 -5.804106 -0.076380
H -2.203020 5.303374 -0.081910
H -4.634663 5.833015 -0.201612
H -6.332010 4.037268 -0.214781
H -5.660528 1.635879 -0.112636
H -0.784097 0.438875 0.055481
H(Iso=2) 4.385797 -0.039423 -0.016806
H 6.832638 -0.002152 -0.823060
H 6.858434 0.092548 0.705856
H -5.339959 -0.399340 0.245996
H -6.500384 -0.445924 1.236819
H -0.195032 5.087475 0.424660
O -0.236046 6.060703 0.468355
H -0.247901 6.253869 1.413463

D3c
E = -1897.001283
C -5.236431 -1.020914 0.028861
C -4.005299 -0.401187 -0.184488
C -3.910377 0.995489 -0.328571
C -5.044120 1.805655 -0.277517
C -6.274370 1.183534 -0.073858
C -6.367915 -0.209595 0.079446
C -2.627703 -0.856906 -0.290894
N -1.755244 0.139726 -0.447206
C -2.486137 1.283829 -0.475558
N -2.019678 2.536307 -0.578709
C -0.749061 2.828916 -0.559583
C -0.218253 4.187992 -0.629202
C 1.172601 4.102608 -0.496715
C 1.515090 2.688483 -0.350159
N 0.339187 1.985714 -0.415500
C -0.856586 5.415108 -0.780673
C -0.053957 6.554044 -0.799087
C 1.341274 6.467374 -0.664812
C 1.976832 5.237232 -0.508828
N 2.732797 2.251244 -0.165886
C 3.012580 0.942975 -0.028069
C 4.389922 0.493620 0.242697
C 4.317829 -0.899524 0.295362
C 2.901637 -1.223696 0.056052
N 2.158407 -0.082901 -0.123944
C 5.587381 1.169012 0.419715
C 6.726854 0.392922 0.658772
C 6.655078 -1.003836 0.712432
C 5.442099 -1.676652 0.529148
N 2.478072 -2.468909 -0.018851
C 1.205849 -2.796737 -0.183193
C 0.731794 -4.113675 -0.539508
C -0.675422 -4.054876 -0.620926
C -1.026028 -2.697792 -0.299081
N 0.098101 -1.989332 -0.057713
C -1.423385 -5.181132 -0.987887
C -0.731668 -6.355564 -1.244095
C 0.674475 -6.414335 -1.155913
C 1.421965 -5.297595 -0.812619
N -2.262365 -2.166832 -0.285244
O -0.218271 -0.322240 2.063258
O -2.541868 -1.748741 2.671577
O -2.800538 1.023023 2.663486
H -5.330228 -2.099924 0.166862
H -7.346074 -0.663344 0.243704
H -7.183494 1.785007 -0.032645
H -4.960997 2.886289 -0.398996
H 3.058257 5.154767 -0.401903
H 1.935400 7.381972 -0.683834

H -0.515763 7.534893 -0.920848
H -1.939982 5.473017 -0.884291
H 0.295333 0.966597 -0.383951
H 5.379362 -2.764341 0.567085
H 7.564470 -1.576831 0.899531
H 7.690520 0.882858 0.805458
H 5.633573 2.257136 0.373579
H -2.510967 -5.152812 -1.076773
H -1.284504 -7.252574 -1.525449
H 1.179127 -7.357613 -1.368208
H 2.509682 -5.327280 -0.751967
H 0.093896 -1.097726 0.482202
H -2.866430 -0.834274 2.780883
H -2.688654 -2.164416 3.529112
H -3.454488 1.392775 2.052936
H -2.824280 1.604346 3.433505
H -0.779819 0.465217 2.017381
H -0.849951 -0.976144 2.423038
H(Iso=2) -3.022817 -2.837694 -0.269087

D4a
E = -1973.421047
C -4.828559 -3.929225 -0.114311
C -4.179984 -2.702171 -0.080442
C -4.889957 -1.501180 -0.058779
C -6.277630 -1.473141 -0.069773
C -6.938253 -2.703982 -0.105235
C -6.225748 -3.910532 -0.127064
C -2.763050 -2.317625 -0.056423
N -2.621173 -0.951218 -0.028097
C -3.870470 -0.443286 -0.022649
N -4.238119 0.847739 0.016279
C -3.364607 1.820674 0.043143
C -3.694830 3.243115 0.080712
C -2.486336 3.954273 0.086778
C -1.402738 2.976061 0.049942
N -2.000145 1.734028 0.033960
C -4.923486 3.895618 0.103171
C -4.905768 5.288072 0.134087
C -3.695620 6.001265 0.139889
C -2.466808 5.345524 0.115033
N -0.132697 3.263927 0.018974
C 0.844995 2.350676 -0.042990
C 2.239686 2.758737 -0.138078
C 2.991071 1.575852 -0.213133
C 1.990986 0.515219 -0.115140
N 0.736389 0.990368 -0.037766
C 2.842046 4.014289 -0.187294
C 4.226167 4.063158 -0.320041
C 4.982440 2.883672 -0.428768
C 4.378047 1.628179 -0.387790
N 2.298178 -0.809813 -0.069041
C 1.449161 -1.845873 -0.021092
C 1.772444 -3.253475 0.060700
C 0.537212 -3.935810 0.042328
C -0.516770 -2.953790 -0.029989
N 0.101163 -1.721373 -0.065776
C 0.474324 -5.329933 0.100549
C 1.672402 -6.023901 0.178794
C 2.906383 -5.344785 0.206639
C 2.979836 -3.959883 0.150599
N -1.806708 -3.228244 -0.047953
O 4.882085 -1.481600 0.311476
O 6.685415 -0.864670 -1.556601
O 6.037155 0.505943 2.029509
O 7.750084 1.168499 -0.106939
H -4.267630 -4.863777 -0.130292
H -6.775682 -4.852433 -0.154220
H -8.028859 -2.729537 -0.115982
H -6.822085 -0.528973 -0.051677
H -1.523455 5.891198 0.117170
H -3.722184 7.091605 0.163469
H -5.847462 5.838205 0.153275
H -5.855185 3.330268 0.096452
H 4.979482 0.731879 -0.522131
H 6.065839 2.931900 -0.552625
H 4.732971 5.028433 -0.360242
H 2.236721 4.919010 -0.128003
H -0.489694 -5.837815 0.084786
H 1.663382 -7.113710 0.224357
H 3.828702 -5.922835 0.277330
H 3.936267 -3.439194 0.194174
H -0.387659 -0.830361 -0.113254
H(Iso=2) 3.324204 -1.054037 0.005999
H 5.526763 -1.371961 -0.433756
H 8.688502 1.315019 0.057432
H 7.358044 0.965535 0.762657
H -1.508576 0.846083 0.025469
H 6.431939 -0.527554 -2.423107
H 7.199411 -0.135718 -1.143953
H 5.284399 -0.971905 1.037425
H 6.228450 0.433483 2.973610
H 5.407774 1.239157 1.951788

D4b
E = -1973.411158
C -5.352008 -2.080367 0.112574
C -4.282498 -1.202909 0.003391
C -4.467519 0.181210 -0.013892
C -5.732313 0.745562 0.078132
C -6.815175 -0.131300 0.190397
C -6.628979 -1.519699 0.207402
C -2.829812 -1.407339 -0.118138
N -2.168820 -0.238950 -0.201406
C -3.119088 0.752134 -0.143717
N -2.915580 2.054680 -0.191632
C -1.720287 2.610690 -0.304719
C -1.466085 4.028764 -0.375685
C -0.070437 4.213889 -0.476943
C 0.502970 2.895784 -0.463311
N -0.490464 1.989295 -0.371459
C -2.338836 5.121206 -0.360732
C -1.786130 6.389336 -0.447514
C -0.391424 6.575764 -0.549936
C 0.481214 5.499076 -0.567356
N 1.807515 2.583808 -0.539932
C 2.397159 1.360901 -0.518093
C 3.839754 1.163819 -0.535018
C 3.985805 -0.232761 -0.543153
C 2.631205 -0.780562 -0.515443
N 1.705734 0.220933 -0.505326
C 4.951433 2.005465 -0.527024
C 6.211159 1.409116 -0.529264
C 6.355939 0.012619 -0.539727
C 5.244051 -0.828399 -0.547190
N 2.408888 -2.096914 -0.516662
C 1.219456 -2.625611 -0.430007
C 0.937938 -4.054699 -0.509564
C -0.450249 -4.213524 -0.406100
C -1.033069 -2.880899 -0.260408
N 0.003777 -1.991521 -0.285318
C -1.045628 -5.469191 -0.461361
C -0.202810 -6.567931 -0.617784
C 1.188626 -6.409457 -0.722210
C 1.780939 -5.148947 -0.673703
N -2.317099 -2.647427 -0.150775
O -0.138500 0.186693 1.995131
O 1.387396 -1.921487 2.484526
O 3.396633 -0.157799 2.601172
O 1.767102 2.054887 2.341546
H -5.196350 -3.159250 0.121812
H -7.498579 -2.172651 0.294531
H -7.826662 0.270777 0.264962
H -5.870663 1.826761 0.061699
H 1.556793 5.665904 -0.651286
H 0.005980 7.588997 -0.618682

H -2.437500 7.264048 -0.438774
H -3.414540 4.964527 -0.283844
H -0.355377 0.982247 -0.281614
H 5.344304 -1.913935 -0.553789
H 7.358293 -0.417565 -0.542664
H 7.102093 2.038107 -0.521768
H 4.863803 3.093998 -0.514676
H -2.127318 -5.577876 -0.384438
H -0.628800 -7.571101 -0.662771
H 1.815878 -7.293406 -0.845932
H 2.859214 -5.015847 -0.758741
H -0.101762 -1.003231 -0.070643
H 2.225461 -1.396078 2.509659
H 1.397975 -2.438123 3.297806
H 2.943042 0.707922 2.504894
H 4.144452 -0.119136 1.994406
H -1.065585 0.072600 2.229738
H 0.316827 -0.651460 2.267643
H 0.969876 1.478626 2.278470
H 1.669593 2.511969 3.184813
H(Iso=2) 2.439046 3.377038 -0.525669

D4c
E = -1973.398024
C 5.552479 -0.445710 -0.060148
C 4.252896 0.054452 -0.026150
C 3.998352 1.434424 0.059369
C 5.038157 2.360440 0.101079
C 6.338995 1.858433 0.064273
C 6.591888 0.482954 -0.013319
C 2.546248 1.608604 0.071123
N 1.997430 0.344682 0.003173
C 2.957787 -0.625256 -0.053855
N 2.782767 -1.924406 -0.106520
C 1.569993 -2.511234 -0.056105
C 1.453315 -3.979100 -0.032989
C 0.081402 -4.240245 0.032871
C -0.564811 -2.918246 0.046524
N 0.368824 -1.906697 -0.008894
C 2.391180 -5.004592 -0.055886
C 1.901131 -6.314159 -0.023257
C 0.529253 -6.575908 0.028902
C -0.410172 -5.540108 0.057527
N -1.878120 -2.787961 0.094333
C -2.534418 -1.634292 0.050276
C -3.969104 -1.481500 0.013629
C -4.251035 -0.097440 -0.057418
C -2.976202 0.572584 -0.052359
N -2.001060 -0.364561 0.007402
C -5.001544 -2.429251 0.020698
C -6.304456 -1.959804 -0.041844
C -6.586727 -0.582046 -0.111345
C -5.573567 0.363641 -0.119628
N -2.741714 1.898208 -0.089743
C -1.545163 2.549294 -0.055351
C -1.417552 4.001141 -0.080397
C -0.033320 4.225778 -0.002915
C 0.582437 2.902359 0.053872
N -0.362680 1.917376 0.017012
C -2.315489 5.065577 -0.166895
C -1.790029 6.355261 -0.175287
C -0.408152 6.577543 -0.100519
C 0.492135 5.517957 -0.014788
N 1.917046 2.752250 0.114306
O -5.161889 3.534124 -0.277351
O 5.183397 -3.534415 -0.666095
H 1.566938 5.697569 0.050754
H -0.029490 7.600388 -0.111909
H -2.466256 7.208547 -0.243800
H -3.389563 4.901566 -0.230222
H -4.784940 -3.497729 0.086293
H -7.129415 -2.673244 -0.037928
H -7.625532 -0.252880 -0.160544
H -5.798632 1.426987 -0.176677
H -1.004364 -0.159665 0.022583
H 3.463481 -4.807296 -0.105684
H 2.605626 -7.146872 -0.040668
H 0.181593 -7.609831 0.048052
H -1.479537 -5.752552 0.103494
H 4.845780 3.432912 0.168874
H 7.177769 2.555202 0.096518
H 7.623212 0.128701 -0.039011
H 5.750064 -1.517358 -0.132275
H 1.003505 0.139503 -0.002469
H(Iso=2) -3.580735 2.492778 -0.144081
H -5.649184 3.958106 0.440596
H -5.567714 3.869922 -1.086878
H 4.363163 -3.016131 -0.566071
H 5.269939 -3.653594 -1.619539
H 3.014251 4.334090 0.604608
O 3.576261 5.125678 0.693870
H 3.639562 5.263444 1.646627
H -2.959037 -4.388286 0.563888
O -3.549802 -5.158368 0.655119
H -3.577123 -5.321966 1.605503

H2O
E = -76.378020
O 0.000000 0.000000 0.119919
H -0.000000 0.754106 -0.479676
H -0.000000 -0.754106 -0.479676
